# Role of Diet in Colorectal Cancer Incidence: Umbrella Review of Meta-analyses of Prospective Observational Studies

**DOI:** 10.1001/jamanetworkopen.2020.37341

**Published:** 2021-02-16

**Authors:** Sajesh K. Veettil, Tse Yee Wong, Yee Shen Loo, Mary C. Playdon, Nai Ming Lai, Edward L. Giovannucci, Nathorn Chaiyakunapruk

**Affiliations:** 1 Department of Pharmacotherapy, College of Pharmacy, University of Utah, Salt Lake City; 2 School of Pharmacy, International Medical University, Bukit Jalil, Kuala Lumpur, Malaysia; 3 Department of Nutrition and Integrative Physiology, University of Utah, Salt Lake City; 4 Huntsman Cancer Institute, Cancer Control and Population Sciences Program, University of Utah, Salt Lake City; 5 School of Medicine, Faculty of Health and Medical Sciences, Taylor’s University, Subang Jaya, Selangor, Malaysia; 6 School of Pharmacy, Monash University Malaysia, Bandar Sunway, Subang Jaya, Selangor, Malaysia; 7 Department of Epidemiology, Harvard T.H. Chan School of Public Health, Boston, Massachusetts; 8 Department of Nutrition, Harvard T.H. Chan School of Public Health, Boston, Massachusetts

## Abstract

**Importance:**

Several meta-analyses have summarized evidence for the association between dietary factors and the incidence of colorectal cancer (CRC). However, to date, there has been little synthesis of the strength, precision, and quality of this evidence in aggregate.

**Objective:**

To grade the evidence from published meta-analyses of prospective observational studies that assessed the association of dietary patterns, specific foods, food groups, beverages (including alcohol), macronutrients, and micronutrients with the incidence of CRC.

**Data Sources:**

MEDLINE, Embase, and the Cochrane Library were searched from database inception to September 2019.

**Evidence Review:**

Only meta-analyses of prospective observational studies with a cohort study design were eligible. Evidence of association was graded according to established criteria as follows: convincing, highly suggestive, suggestive, weak, or not significant.

**Results:**

From 9954 publications, 222 full-text articles (2.2%) were evaluated for eligibility, and 45 meta-analyses (20.3%) that described 109 associations between dietary factors and CRC incidence were selected. Overall, 35 of the 109 associations (32.1%) were nominally statistically significant using random-effects meta-analysis models; 17 associations (15.6%) demonstrated large heterogeneity between studies (*I*^2^ > 50%), whereas small-study effects were found for 11 associations (10.1%). Excess significance bias was not detected for any association between diet and CRC. The primary analysis identified 5 (4.6%) convincing, 2 (1.8%) highly suggestive, 10 (9.2%) suggestive, and 18 (16.5%) weak associations between diet and CRC, while there was no evidence for 74 (67.9%) associations. There was convincing evidence of an association of intake of red meat (high vs low) and alcohol (≥4 drinks/d vs 0 or occasional drinks) with the incidence of CRC and an inverse association of higher vs lower intakes of dietary fiber, calcium, and yogurt with CRC risk. The evidence for convincing associations remained robust following sensitivity analyses.

**Conclusions and Relevance:**

This umbrella review found convincing evidence of an association between lower CRC risk and higher intakes of dietary fiber, dietary calcium, and yogurt and lower intakes of alcohol and red meat. More research is needed on specific foods for which evidence remains suggestive, including other dairy products, whole grains, processed meat, and specific dietary patterns.

## Key Points

**Question:**

How credible is the evidence behind the association of dietary factors with colorectal cancer (CRC) risk in published meta-analyses of prospective observational studies?

**Findings:**

This umbrella review of 45 meta-analyses describing 109 associations found convincing evidence for an association between lower CRC risk and higher intakes of dietary fiber, dietary calcium, and yogurt and lower intakes of alcohol and red meat.

**Meaning:**

This study suggests that dietary factors may have a role in the development and prevention of CRC, but more research is needed on specific foods for which the evidence remains suggestive.

## Teaser

This umbrella review grades the evidence from published meta-analyses of prospective observational studies that assessed the association of dietary patterns, specific foods, food groups, beverages (including alcohol), macronutrients, and micronutrients with the incidence of colorectal cancer (CRC).

## Introduction

Colorectal cancer (CRC) is the third most commonly diagnosed cancer among men and the second most common cancer among women worldwide.^1^ The etiology of CRC is multifactorial, with both genetic and environmental factors playing a role.^2^ Evidence suggests that modifiable lifestyle factors, including excess adiposity, poor diet, and physical inactivity, play an important role in the occurrence and progression of this disease.^2,3^

Several systematic reviews with meta-analysis of prospective observational studies have summarized evidence for the associations between dietary factors (eg, foods and food groups, beverages, alcohol, macronutrients, and micronutrients) and the incidence of CRC. However, to date, there has been little synthesis of the strength, precision, and quality of this evidence in aggregate. Umbrella reviews provide a structured and critical summary of the evidence and enable the grading of evidence according to specific criteria: sample size, strength and precision of the association, and assessment of the presence of biases.^4,5,6^ The review from the World Cancer Research Fund (WCRF) in partnership with the American Institute for Cancer Research (AICR) provided a summary report based on systematic reviews with dose-response meta-analysis of the association between dietary exposures and CRC. The WCRF/AICR report applied many, but not all, umbrella review criteria for evidence grading and was based on studies published until April 2015.^7^ To complement the WCRF/AICR report, we conducted an umbrella review of meta-analyses to provide an updated and unified systematic summary of epidemiological data evaluating the strength of the overall body of evidence investigating the associations between dietary factors and CRC incidence. Furthermore, we expand the review of dietary exposures to some not previously evaluated, such as certain dietary patterns.

## Methods

The protocol of this umbrella review has been registered in PROSPERO (CRD42020173636). This study adhered to the Meta-analysis of Observational Studies in Epidemiology (MOOSE) reporting guideline.

### Search Strategy and Eligibility Criteria

We searched MEDLINE, Embase, and the Cochrane Library from database inception to September 2019 to identify meta-analyses of prospective, observational studies. The search strategy can be found in eTable 1 in the Supplement.

Two authors (Y.S.L. and T.Y.W.) independently screened titles or abstracts and examined the full text of potentially eligible articles. Discrepancies were resolved by a third reviewer (S.K.V.).

Studies were included that met the following criteria: (1) meta-analysis of prospective observational studies (ie, cohort design) among adults with multivariable-adjusted summary risk estimates and corresponding 95% CIs that (2) investigated the association of dietary factor(s) with the incidence of CRC. Eligible dietary factors included dietary patterns, prespecified diet quality indices, specific foods, food groups, beverages (including alcohol), macronutrients (ie, carbohydrates, fat, protein), and micronutrients (ie, vitamins, minerals, antioxidants, polyphenols). When more than 1 meta-analysis on the same research question was available, we assessed only the study that included the largest data set, as previously described (eAppendix in the Supplement).^4,6^ We excluded meta-analyses of studies with other study designs and those with insufficient or inadequate data for quantitative synthesis.

### Data Extraction

Data were extracted, and the methodological quality of included meta-analyses was assessed using A Measurement Tool to Assess Systematic Reviews (AMSTAR-2)^8^ by 2 authors (Y.S.L. and T.Y.W.) independently and checked by a third author (S.K.V.). For each eligible meta-analysis, we abstracted data at the meta-analysis level (eAppendix in the Supplement). Disagreements were resolved by consensus.

### Statistical Analysis

For each association of diet with CRC, we recalculated the adjusted summary estimates and corresponding 95% CIs with *P* values using the DerSimonian and Laird random-effects model.^9^ Heterogeneity was assessed with the *I*^2^ statistic.^10^ We estimated the 95% prediction interval (PrI), which evaluates the uncertainty for the effect size that would be anticipated in a new study addressing the identical association.^11^ The evidence for small-study effects was assessed by Egger regression asymmetry test.^12^ *P* < .10 was taken as statistical evidence of the presence of small-study effects. We also applied the excess significance test, which evaluates whether the observed number of studies with statistically significant results (positive studies, *P* ≤.05) differs from the expected number of positive studies, by using a χ^2^ test.^13^ Excess significance bias was set at *P* ≤ .10. The statistical analysis and power calculations were conducted using Stata version 15.0 (StataCorp).

#### Evaluation of the Quality of Evidence

We graded the quality of the evidence per association generated by a meta-analysis by applying several criteria in concordance with previously published umbrella reviews.^4,5,6^ In brief, associations that presented nominally significant random-effects summary effect sizes (ie, *P* ≤ .05) were graded as convincing (class I), highly suggestive (class II), suggestive (class III), or weak (class IV) evidence (Table 1).

**Table 1.** Criteria for Quality of Evidence Classification in Observational Studies^a^

Category	Criteria
Convincing, class 1	No. of cases >1000 *P* < 1 × 10^−6^ *I*^2^ < 50% 95% prediction interval excluding the null No small-study effects No excess significance bias
Highly suggestive, class II	No. of cases >1,000 *P* < 1 × 10^−6^ Largest study with a statistically significant effect
Suggestive, class III	No. of cases >1,000 *P* < 1 × 10^−3^
Weak, class IV	*P* < .05
Nonsignificant	*P* > .05

^a^ Criteria in concordance with previously published umbrella reviews.^4,5,6^

#### Sensitivity Analyses

For each meta-analysis initially graded as showing convincing, highly suggestive, or suggestive evidence, we reexamined the list of adjusted confounding factors at the component study level. We performed a sensitivity analysis by including only adjusted estimates to assess the robustness of the main analysis. Based on the literature, the most consistent potential confounders considered for sensitivity analysis are age, body mass index (BMI), race, sex, and other dietary factors as observed in our umbrella review. Other sensitivity analyses included the omission of small-sized studies (ie, <25th percentile)^14^ from those meta-analyses with evidence of small-study effects, as detailed under the heading of statistical analyses and low-quality studies (as defined in each meta-analysis).

## Results

In total, we identified 9954 publications, evaluated 222 full-text articles (2.2%), and included 45 meta-analyses (20.3%)^15,16,17,18,19,20,21,22,23,24,25,26,27,28,29,30,31,32,33,34,35,36,37,38,39,40,41,42,43,44,45,46,47,48,49,50,51,52,53,54,55,56,57,58,59^ describing 109 associations in this umbrella review (eFigure in the Supplement). The 177 articles (79.7%) that were excluded and the reasons for their exclusion are provided in eTable 2 in the Supplement. The descriptive characteristics of the 45 eligible meta-analyses^15,16,17,18,19,20,21,22,23,24,25,26,27,28,29,30,31,32,33,34,35,36,37,38,39,40,41,42,43,44,45,46,47,48,49,50,51,52,53,54,55,56,57,58,59^ are provided in eTable 3 in the Supplement. The included meta-analyses were published between 2006 and 2019. The median (interquartile range [IQR]) number of studies per meta-analysis was 6 (3-9), and the median (IQR) duration of follow-up was 10.2 (9.3-12.9) years. The median (IQR) meta-analysis sample size was 598 744 (229 046-991 476). The median (IQR) number of cases (ie, incidence of CRC) was 5076 (2673-9355) cases, and the number of cases was greater than 1000 for 99 associations (90.8%).

The evaluation of methodological quality using AMSTAR-2 (Table 2 and Table 3; eTable 3 in the Supplement) revealed that 2 meta-analyses (4.4%) were of high quality and 15 meta-analyses (33.3%) were of moderate quality. Twenty meta-analyses (44.4%) were of low quality, with the remaining 8 (17.8%) rated as having critically low quality.

**Table 2.** Quality of Evidence of Associations Between the Role of Diet and Increase in Colorectal Cancer Incidence

Classification	Exposure	Source	Comparison	Summary metric	Credibility assessment									AMSTAR-2
					Random effect size (95% CI)	*P* value	*I*^2^, %	Largest study 95% CI	Prediction interval 95% CI	Egger *P* value	Excess significance test		Quality of evidence class	
											O/E	*P* value		
Food	Red meat	Schwingshackl et al,^56^ 2018	High vs low	RR	1.13 (1.08-1.19)	<1 × 10^−6^	20.5	1.15-1.19	1.02-1.26	.18	3/6.0	NP	I	High
Alcoholic beverage	Alcohol	Fedirko et al,^31^ 2011	≥4 drinks/d vs non-/occasional drinkers	RR	1.58 (1.38-1.80)	<1 × 10^−6^	0.0	1.27-2.16	1.33-1.87	.80	5/5.8	NP	I	Moderate
Alcoholic beverage	Alcohol	Fedirko et al,^31^ 2011	>1-3 drinks/d vs non-/occasional drinkers	RR	1.24 (1.14-1.34)	<1 × 10^−6^	49.3	1.01-1.13	0.95-1.61	<.001	9/2.8	.77	II	Moderate
Food	Processed meat	Schwingshackl et al,^56^ 2018	High vs low	RR	1.14 (1.07-1.23)	.0001	25.9	1.09-1.32	0.97-1.35	.98	4/6.9	NP	III	High
Dietary behavior	Adherence to Western diet	Feng et al,^16^ 2017	High vs low	OR	1.28 (1.13-1.45)	.0001	72.2	1.09-1.44	0.79-2.07	.17	8/6.5	>.99	III	Moderate
Food	Eggs	Schwingshackl et al,^56^ 2018	High vs low	RR	1.36 (1.10-1.68)	.004	0.0	1.10-1.78	0.35-5.31	.28	1/1.2	NP	IV	High
Dietary behavior	Adherence to alcohol drinking	Feng et al,^16^ 2017	High vs low	OR	1.53 (1.04-2.25)	.03	93.5	1.54-2.10	0.37-6.34	.56	3/8.3	NP	IV	Moderate
Alcoholic beverage	Beer	Zhang et al,^32^ 2015	Drinkers vs non-/occasional drinkers	RR	1.08 (1.02-1.15)	.01	0.0	1.04-1.28	1.01-1.16	.52	1/1.5	NP	IV	Moderate
Dietary behavior	Adherence to unhealthy diet^a^	Grosso et al,^27^ 2017	High vs low	RR	1.13 (1.03-1.23)	.007	30.5	1.02-1.36	0.89-1.43	.76	2/3.7	NP	IV	Low
Food	Pork	Carr et al,^57^ 2016	High vs low	RR	1.17 (1.04-1.31)	.01	0.0	0.93-1.38	0.90-1.51	.86	0/0.7	NP	IV	Low
Micronutrient	Heme iron	Qiao et al,^51^ 2013	High vs low	RR	1.12 (1.01-1.24)	.03	22.6	0.99-1.29	0.90-1.39	.81	1/NA	NA	IV	Critically low

Abbreviations: AMSTAR-2, A Measurement Tool to Assess Systematic Reviews; NA, not applicable because of nonsignificant effect estimate; NP, not pertinent because estimated number is larger than observed and there is no evidence of excess significance based on assumption made for plausible effect size; O/E, observed/expected number of studies with significant results; OR, odds ratio; RR, risk ratio.

^a^ Unhealthy diet was defined by authors as characterized by, but not limited to, red and processed meat, sugary drinks and salty snacks, starchy foods, and refined carbohydrates.

**Table 3.** Quality of Evidence of Associations Between the Role of Diet and Reduction in Colorectal Cancer Incidence

Classification	Exposure	Source	Comparison	Summary metric	Credibility assessment									AMSTAR-2
					Random effect size (95% CI)	*P* value	*I*^2^, %	Largest study 95% CI	Prediction interval 95% CI	Egger *P* value	Excess significance test		Quality of evidence class	
											O/E	*P* value		
Macronutrient	Total dietary fiber	Reynolds et al,^49^ 2019	High vs low	RR	0.84 (0.78-0.89)	<1 × 10^−6^	18.1	0.65-0.85	0.72-0.97	.53	6/12.1	NP	I	High
Micronutrient	Dietary calcium	Meng et al,^50^ 2019	High vs low	HR	0.77 (0.73-0.82)	<1 × 10^−6^	0.0	0.75-0.94	0.72-0.83	.60	5/3.9	>.99	I	Moderate
Food	Yogurt	Zhang et al,^24^ 2019	High vs low	OR	0.81 (0.76-0.86)	<1 × 10^−6^	0.0	0.75-0.87	0.72-0.90	.84	2/1.8	>.99	I	Moderate
Food	Dairy products	Schwingshackl et al,^56^ 2018	High vs low	RR	0.83 (0.76-0.89)	<1 × 10^−6^	60.3	0.83-0.95	0.65-1.04	.17	8/4.0	.99	II	High
Food	Whole grains	Schwingshackl et al,^56^ 2018	High vs low	RR	0.88 (0.83-0.94)	.00006	34.9	0.88-0.99	0.77-1.01	.07	4/1.0	.26	III	High
Dietary behavior	Adherence to healthy diet^a^	Feng et al,^16^ 2017	High vs low	OR	0.84 (0.76-0.92)	.0003	56.2	0.69-0.90	0.60-1.17	.60	5/7.5	NP	III	Moderate
Beverage	Nonfermented milk	Ralston et al,^30^ 2014	High vs low	RR	0.85 (0.78-0.93)	.0005	0.0	0.78-1.18	0.77-0.94	.96	3/0.9	.96	III	Moderate
Dietary behavior	Pesco-vegetarian diet	Godos et al,^38^ 2016	Yes vs no	RR	0.67 (0.53-0.83)	.0004	0.0	0.48-0.94	0.15-2.89	.44	2/1.7	.94	III	Low
Dietary behavior	Semivegetarian diet	Godos et al,^38^ 2016	Yes vs no	RR	0.86 (0.79-0.94)	.0007	0.0	0.76-0.95	0.72-1.04	.96	1/1.2	NP	III	Low
Micronutrient	Supplemental calcium	Heine-Bröring et al,^47^ 2015	Yes vs no	RR	0.88 (0.82-0.94)	.00009	51.7	0.88-1.05	0.70-1.09	.07	3/3.4	NP	III	Low
Micronutrient	Supplemental calcium	Heine-Bröring et al,^47^ 2015	High vs low	RR	0.80 (0.72-0.89)	.00002	30.9	0.72-1.02	0.63-1.01	.88	4/2.7	.95	III	Low
Dietary behavior	Adherence to Mediterranean diet	Schwingshackl et al,^15^ 2017	High vs low	RR	0.86 (0.80-0.92)	8.4 × 10^−6^	29.7	0.80-0.99	0.74-1.00	.84	3/5.0	NP	III	Critically low
Food	Vegetables	Schwingshackl et al,^56^ 2018	High vs low	RR	0.96 (0.92-1.00)	.04	25.0	0.96-1.04	0.87-1.05	.26	1/0.9	>.99	IV	High
Food	Fruits	Schwingshackl et al,^56^ 2018	High vs low	RR	0.93 (0.87-0.99)	.01	51.8	0.96-1.06	0.78-1.10	.16	3/0.9	.99	IV	High
Micronutrient	Zinc	Li et al,^53^ 2014	High vs low	RR	0.82 (0.69-0.96)	.02	67.0	0.68-0.78	0.48-1.38	.43	2/3.8	NP	IV	Moderate
Food	Legumes	Zhu et al,^21^ 2015	High vs low	RR	0.90 (0.83-0.98)	.01	46.1	0.83-1.09	0.66-1.23	.13	5/1.0	.16	IV	Low
Macronutrient	Cereal fiber	Aune et al,^40^ 2011	High vs low	RR	0.90 (0.83-0.96)	.003	0.0	0.76-0.98	0.82-0.98	.36	1/2.9	NP	IV	Low
Micronutrient	Multivitamin	Heine-Bröring et al,^47^ 2015	Yes vs no	RR	0.91 (0.86-0.97)	.002	13.7	0.88-1.11	0.81-1.02	.55	2/0.8	.91	IV	Low
Micronutrient	Vitamin A	Heine-Bröring et al,^47^ 2015	Yes vs no	RR	0.76 (0.62-0.94)	.01	0.0	0.50-1.10	0.48-1.21	.78	1/1.7	NP	IV	Low
Micronutrient	Vitamin B_6_	Liu et al,^48^ 2015	High vs low	RR	0.88 (0.79-0.99)	.03	47.2	0.66-0.99	0.62-1.26	.70	5/7.1	NP	IV	Low
Micronutrient	Folic acid	Liu et al,^48^ 2015	High vs low	RR	0.88 (0.81-0.95)	.002	42.9	0.58-0.84	0.68-1.15	.08	7/13.7	NP	IV	Low
Micronutrient	Vitamin D	Liu et al,^48^ 2015	High vs low	RR	0.85 (0.75-0.97)	.01	44.1	0.83-1.33	0.57-1.26	.46	2/1.5	>.99	IV	Low
Micronutrient	Vitamin E	Heine-Bröring et al,^47^ 2015	Yes vs no	RR	0.87 (0.75-1.00)	.048	0.0	0.64-1.36	0.73-1.03	.75	1/NA	NA	IV	Low
Micronutrient	Magnesium	Chen et al,^52^ 2012	High vs low	RR	0.87 (0.77-0.99)	.03	0.5	0.62-1.03	0.75-1.02	.68	0/3.5	NP	IV	Critically low

Abbreviations: AMSTAR-2, A Measurement Tool to Assess Systematic Reviews; HR, hazard ratio; NA, not applicable because of nonsignificant effect estimate; NP, not pertinent because estimated number is larger than observed and there is no evidence of excess significance based on assumption made for plausible effect size; O/E, observed/expected number of studies with significant results; OR, odds ratio; RR, risk ratio.

^a^ Healthy diet was defined by the authors as high intakes of vegetables, fruits, whole grains, olive oil, fish, soy, poultry, and low-fat dairy.

### Description and Summary of Associations

Overall, the 45 meta-analyses described 109 associations, including 794 individual study estimates of CRC incidence associated with dietary exposures. The included meta-analyses provided adjusted summary estimates on the associations between dietary patterns^15,16,27,38,49,55^ (13 associations), food groups^17,18,19,20,21,22,23,24,56,57,58,59^ (23 associations), beverages including alcohol^25,26,28,29,30,31,32,33^ (12 associations), macronutrients^34,35,36,37,39,40,49,59^ (18 associations), and micronutrients^41,42,43,44,45,46,47,48,50,51,52,53,54^ (43 associations) and the incidence of CRC. Definitions of dietary patterns is provided in the eAppendix in the Supplement.

A total of 35 of the 109 associations (32.1%) were nominally statistically significant at *P* ≤ .05 (Table 2 and Table 3). Of these, only 7 associations (20.0%) reached statistical significance at *P* ≤ 1 × 10^−6^. Overall, 24 significant associations (68.6%) suggested potential protective effects of dietary factors or dietary patterns associated with CRC risk, including adherence to a healthy diet, Mediterranean diet, pesco-vegetarian diet, or semivegetarian diet and higher intakes of dietary fiber, whole grains, legumes, dairy products including yogurt and nonfermented milk, fruits and vegetables, and micronutrients (ie, supplemental and dietary calcium, zinc, magnesium, vitamin A, vitamin B_6_, folic acid, vitamin D, vitamin E). The remaining significant associations (31.4%) suggested higher risk of CRC with adherence to an unhealthy diet or Western diet and increased intake of alcohol, red meat, processed meat, pork, eggs, and haem iron.

Seventeen associations (15.6%) had large heterogeneity (*I*^2 ^> 50%). The 95% PrIs excluded the null value for 8 associations (7.3%). The effect sizes of the largest study were statistically significant at *P* ≤ .05 for 29 associations (26.6%). Small-study effects were found for 11 associations (10.1%), and excess significance bias was not identified. Seventy-four (67.9%) associations without statistical significance at *P* ≤ .05 using random-effects models are presented in eTable 4 in the Supplement.

### Main Analysis Grading

#### Convincing Evidence

Among the 109 associations, 5 (4.6%) were supported by convincing evidence (Table 2 and Table 3). Two of these associations (40.0%), ie, higher vs lower red meat intake (AMSTAR-2, high quality) and heavy alcohol intake (defined as >4 drinks per day compared with those who did not drink or occasionally drank) (AMSTAR-2, moderate quality), were associated with increased risk of CRC. In contrast, convincing evidence was found for 3 inverse associations: higher vs lower intake of total dietary fiber (AMSTAR-2, high quality), calcium (AMSTAR-2, moderate quality), and yogurt (AMSTAR-2, moderate quality) were associated with reduced CRC incidence.

#### Highly Suggestive Evidence

Two associations (1.8%) had highly suggestive evidence of an association between diet and incidence of CRC (Table 2 and Table 3). Higher intake of total dairy products (eg, milk, cheese, yogurt) (AMSTAR-2, high quality) was associated with significant CRC risk reduction compared with lower intake. However, a moderate intake of alcohol (defined as >1-3 drinks but not more than 4 per day) (AMSTAR-2, moderate quality) was associated with an increase in the incidence of CRC compared with 0 drinks or occasionally drinking.

#### Suggestive, Weak, and No Evidence

Suggestive evidence for the association of diet or dietary patterns with reduced risk of CRC was found for 8 associations (7.3%), including adherence to the Mediterranean diet, adherence to a healthy diet, adherence to a pesco-vegetarian diet, adherence to a semivegetarian diet, and intake of whole grains, nonfermented milk, and supplemental calcium (Table 3). However, there was suggestive evidence that 2 exposures (adherence to the Western diet and intake of processed meat) were associated with increased risk of CRC in adults (Table 2). The remaining associations, with either weak evidence (18 [16.5%]) or no evidence (74 [67.9%]) are provided in Table 2, Table 3, and eTable 4 in the Supplement.

### Sensitivity Analyses

Results from sensitivity analyses are reported in eTable 5 in the Supplement. Among convincing associations for increased risk of CRC, only alcohol exposure showed evidence for small-study effects. Removal of small studies from the alcohol analysis did not modify the evidence rating. When excluding low-quality studies and those that did not adjust for important potential confounders, both the association of alcohol intake and red meat intake with CRC retained their evidence ratings. For inverse associations, all retained the class I rank following sensitivity analyses. A summary of all sensitivity analyses is presented in Table 4.

**Table 4.** Summary of Sensitivity Analyses

Exposure	Comparison	Quality of evidence, class				CES/AMSTAR-2
		Primary analysis	Sensitivity analyses			
			Including studies adjusted for potential confounding variables	Omission of small-sized studies	Omission of low-quality studies	
Dietary behaviors						
Adherence to Mediterranean diet in Schwingshackl et al,^15^ 2017	High vs low	III	III	NA	III^a^	III/Critically low
Adherence to Western diet in Feng et al,^16^ 2017	High vs low	III	III	NA	III	IV/Moderate
Adherence to healthy diet in Feng et al,^16^ 2017	High vs low	III	III	NA	III	IV/Moderate
Pesco-vegetarian diet in Godos et al,^38^ 2016	Yes vs no	III	III	NA	III^b^	III/Low
Semivegetarian diet in Godos et al,^38^ 2016	Yes vs no	III	III	NA	III^b^	III/Low
Food						
Red meat in Schwingshackl et al,^56^ 2018	High vs low	I	I	NA	I^a^	I/High
Processed meat in Schwingshackl et al,^56^ 2018	High vs low	III	III	NA	III^a^	III/High
Whole grains in Schwingshackl et al,^56^ 2018	High vs low	III	III	III	III^a^	III/High
Dairy products in Schwingshackl et al,^56^ 2018	High vs low	II	II	NA	II^a^	III/High
Yogurt in Zhang et al,^24^ 2019	High vs low	I	I	NA	I^a^	I/Moderate
Beverages						
Moderate alcohol in Fedirko et al,^31^ 2011	>1-3 drinks/d vs non-/occasional drinkers	II	II	III	III	III/Moderate
Heavy alcohol in Fedirko et al,^31^ 2011	≥4 drinks/d vs non-/occasional drinkers	I	I	NA	I	I/Moderate
Nonfermented milk in Ralston et al,^30^ 2014	High vs low	III	III	NA	III	IV/Moderate
Micronutrients						
Total dietary fiber in Reynolds et al,^49^ 2019	High vs low	I	I	NA	I	I/High
Dietary calcium in Meng et al,^50^ 2019	High vs low	I	I	NA	I^b^	I/Moderate
Supplemental calcium in Heine-Bröring et al,^47^ 2015	Yes vs no	III	III	III	III^a^	III/Low
Supplemental calcium in Heine-Bröring et al,^47^ 2015	High vs low	III	III	NA	III^a^	III/Low

Abbreviations: AMSTAR-2, A Measurement Tool to Assess Systematic Reviews; CES, class of evidence after sensitivity analyses; NA, not applicable because sensitivity analysis was not performed because of no evidence of small-study effects.

^a^ No information on quality assessment of primary studies.

^b^ Meta-analysis reported all good-quality studies.

## Discussion

We included 45 published meta-analyses, which comprised 109 adjusted summary risk estimates for the associations of dietary factors with CRC incidence. We found that few of the 35 statistically significant associations (ie, positive associations of 4 drinks/d and red meat with the incidence of CRC and inverse associations of higher intake of dietary fiber, calcium, and yogurt with the incidence of CRC) were supported by convincing evidence in the main and sensitivity analyses. Suggestive evidence exists for an inverse association of a healthy dietary pattern, Mediterranean diet, pesco-vegetarian diet, and semivegetarian diet and intake of whole grains, total dairy products, and supplemental calcium with CRC incidence. Suggestive evidence also exists for positive associations between higher intakes of processed meat and a moderate intake of alcohol (>1-3 drinks/d) and the incidence of CRC.

The continuous update project (CUP) report by the WCRF in partnership with the AICR conducted a comprehensive review including meta-analyses to draw conclusions about the roles of diet, nutrition, and physical activity in cancer prevention and survival.^60^ Although the WCRF/AICR report provides rigorous estimates for the association of diet with CRC incidence, their criteria for study inclusion and grading of evidence differed from the current umbrella review (eTable 6 in the Supplement).^7^ The current umbrella review widened the scope of dietary exposure evaluation to include dietary patterns, in particular, providing evidence to support following a Mediterranean diet or overall dietary patterns that exclude red meat (ie, semivegetarian and pesco-vegetarian diets). In addition, yogurt, which was not evaluated as a discrete exposure in the WCRF/AICR CUP report, was identified as being associated with reduced risk of CRC.

Our findings largely support existing cancer prevention dietary guidance regarding increasing consumption of dietary fiber and dairy products and limiting intake of red meat and alcoholic beverages.^7,61,62,63^ However, we did not find convincing evidence to support limiting consumption of processed meat for CRC prevention. Our results confirmed the positive association of processed meat products with CRC; however, its credibility was suggestive. We did not measure dose-response associations owing to data limitations. However, we examined the summary effect size estimate for concordance regarding the direction of association and level of statistical significance to other eligible meta-analyses reporting the same association (eTable 7 in the Supplement). Findings were concordant with our primary analysis regarding magnitude and direction of the association. Nonetheless, proposed biological mechanisms are highly plausible and the associations have been consistent across studies, with coherence between findings in humans and preclinical studies,^64^ providing support for a causal relationship. It has to be noted that the criteria in this article did not explicitly account for biologic plausibility as part of the determination for association.

Although we found convincing evidence that a higher intake of dietary fiber was associated with reduced risk of CRC, the evidence for different sources of fiber remains low. We observed a suggestive inverse association between whole grains and CRC incidence. Fiber derived from whole grains is accompanied by micronutrients, such as folate, that have demonstrated associations with CRC, but less evidence supports a role in carcinogenesis for other fiber sources (eg, vegetables, fruit, and legumes). We observed a weak inverse association between fruit and vegetable consumption and CRC incidence. Fruit and vegetables are abundant sources not only of fiber, but also of micro- and macronutrients with antitumor properties; thus, they are plausible targets for dietary prevention.^65^ Although plausible and consistent, the associations of fruit and vegetable intake with CRC were also reported as weak in the WCRF/AICR reports,^7,60^ which noted differences in association according to sex as well as nonlinear associations that may attenuate summary estimates.

Our findings convincingly highlight dietary calcium and yogurt as associated with reduced risk of CRC incidence. Findings for cheese and milk were null and weak, respectively. We also found suggestive evidence for lower CRC risk with higher intakes of supplemental calcium. These findings are complementary given that all dietary factors are sources of calcium, which binds to unconjugated bile acids and free fatty acids in the colonic lumen to minimize their toxic effects.^66^ Whether dietary calcium is a causal factor in CRC prevention is difficult to determine given that other correlated constituents (eg, fortified vitamin D) could account for the observations.^67^ The high fat content of cheese and cream may hinder their protective associations, possibly by increasing bile acid and fatty acid excretion in the colonic lumen. Other nutrients or bioactive compounds in dairy products, such as lactoferrin or generation of butyrate, may also play a role.^67^ The biologic plausibility of yogurt’s associations with reduced risk of CRC stems from the presence of lactic acid–producing bacteria (*Lactobacillus bulgaricus* and *Streptococcus thermophilus*) that are purported to reduce levels of carcinogens such as nitroreductase, fecal-activated bacterial enzymes, and soluble fecal bile acids.^68,69,70^ Yogurt intake has been shown to reduce the risk of colorectal adenomas with high malignant potential independent of calcium and nonyogurt dairy intake.^71^ Its protective association could also be mediated through its calcium content and modulated by the gut microbiome.

Selected meta-analyses provide suggestive evidence that supports the adoption of an overall healthy dietary pattern, Mediterranean diet, pesco-vegetarian diet, and semivegetarian diet to prevent CRC, in contrast with Western dietary patterns that are associated with increased CRC risk. Overall dietary pattern accounts for the totality of dietary components in combination and their synergistic or antagonistic influence on human metabolism and disease. Prudent dietary patterns, characterized by higher intakes of vegetables, fruit, whole grains, and low-fat dairy products and lower intakes of alcohol and meat products, are often accompanied by a healthier lifestyle; the converse is true for Western-type dietary patterns. Hence, the suggestive association observed for these dietary behaviors could be influenced by the totality of a healthy or unhealthy lifestyle, in addition to combination(s) of protective or harmful dietary factors.

### Limitations

This study has limitations. A possible limitation of our review was exclusion of dose-response meta-analyses because the data needed for predictive interval estimation and assessment of small study and excess significant bias effects were not available in those articles. Randomized clinical trials, which account for confounding by design, are scarce in research on associations between diet and cancer owing to cost, the long follow-up time required for cancer end points, and ethical concerns. Consequently, we restricted the current umbrella review to meta-analyses of prospective observational studies. Some forms of bias, such as recall bias owing to self-reported diet, are possible but likely to be nondifferential, which would attenuate the observed associations. We generated estimates of publication bias by assessing small-study effects using the Egger test. However, the Egger test is not recommended with the inclusion of less than 10 studies.^72^ Although the small-study effect was indicated for only 11 meta-analyses (10.1%), among these 4 (36.4%) included 5 to 10 studies and 2 (18.2%) included less than 5 studies. However, we were unable to perform alternate tests, such as the Peters test, because this required cases and noncases for each level of exposure, and this information was largely lacking in the meta-analyses.^73^ Thus, more research is needed to investigate the associations based on small numbers of included studies. A further limitation is that we did not conduct subgroup analysis (eg, by sex, age group, or location of cancer, such as colon or rectum) because of the lack of data for grading the quality of evidence for most of the exposures. Dietary associations with CRC may differ according to sex and tumor location, as was highlighted by the WCRF/AICR reports.^7,60^

## Conclusions

The findings of this study support existing recommendations for diet in the primary prevention of CRC, emphasizing higher intakes of dietary fiber, calcium, and yogurt and lower intakes of red meat and alcohol. Emerging evidence supports a possible role for overall dietary patterns that, in totality, emphasize habitually consuming fruits, vegetables, grains, and low-fat dairy and reducing red meat and alcohol intake. More research is needed on specific foods for which evidence remains suggestive, including other dairy products, whole grains, processed meat, and specific dietary patterns.

## Notes

Supplement. **eAppendix. **Supplementary Methods **eTable 1. **Search Strategy **eFigure. **Study Flow Diagram **eTable 2. **Excluded Studies **eTable 3. **Descriptive Characteristics of Included Meta-analyses **eTable 4. **Associations With Nonsignificant Evidence **eTable 5. **Sensitivity Analyses for Associations With Class I, II, or III Evidence **eTable 6. **Evidence Criteria: Difference Between and Comparison of WCRF and Present Review **eTable 7. **Summary Estimates for Concordance in Meta-analyses: Red Meat Intake and Incidence of CRC **eReferences. **
