# Supplementary material for: Role of Diet in Colorectal Cancer Incidence: Umbrella Review of Meta-analyses of Prospective Observational Studies
Source: JAMA Netw Open. 2021 Feb 16;4(2):e2037341. doi: 10.1001/jamanetworkopen.2020.37341 (PMC7887658; doi:10.1001/jamanetworkopen.2020.37341)
Supplement: Supplement. — eAppendix. Supplementary Methods eTable 1. Search Strategy eFigure. Study Flow Diagram eTable 2. Excluded Studies eTable 3. Descriptive Characteristics of Included Meta-analyses eTable 4. Associations With Nonsignificant Evidence eTable 5. Sensitivity Analyses for Associations With Class I, II, or III Evidence eTable 6. Evidence Criteria: Difference Between and Comparison of WCRF and Present Review eTable 7. Summary Estimates for Concordance in Meta-analyses: Red Meat Intake and Incidence of CRC eReferences. [file jamanetwopen-e2037341-s001.pdf]

Supplemental Online Content

Veettil SK, Wong TY, Loo YS, et al. Role of diet in colorectal cancer incidence: umbrella review of meta-analyses of prospective observational studies. *JAMA Netw Open*. 2021;4(2):e2037341. doi:10.1001/jamanetworkopen.2020.37341

eAppendix. Supplementary Methods
eTable 1. Search Strategy
eFigure. Study Flow Diagram
eTable 2. Excluded Studies
eTable 3. Descriptive Characteristics of Included Meta-analyses
eTable 4. Associations With Nonsignificant Evidence
eTable 5. Sensitivity Analyses for Associations With Class I, II, or III Evidence
eTable 6. Evidence Criteria: Difference Between and Comparison of WCRF and Present Review
eTable 7. Summary Estimates for Concordance in Meta-analyses: Red Meat Intake and Incidence of CRC
eReferences.

This supplemental material has been provided by the authors to give readers additional information about their work.

© 2021 Veettil SK et al. *JAMA Network Open.*

eAppendix. Supplementary Methods

We followed relevant sections of the Meta-analysis of Observational Studies in Epidemiology (MOOSE) guidelines.

Umbrella reviews are useful tools that provide a comprehensive overview of evidence of published systematic reviews and meta-analyses on a specific topic. They can elucidate the strength of evidence and the precision of the estimates, and evaluate risk of bias of the published reports. Our objective in this study is to grade the evidence from published meta-analyses of prospective observational studies that assessed the association between dietary patterns, specific foods, food groups, beverages (including alcohol), macronutrients, and micronutrients and incidence of colorectal cancer (CRC). Definition of different dietary patterns is provided below:

Research questions: 1) Which dietary factors are associated with the incidence of colorectal cancer in the general adult population? 2) How credible is the evidence behind these associations in published meta-analyses of prospective observational studies?

Eligibility criteria: PICO characteristics: population-adults of any age; exposure-any dietary patterns, pre-specified diet quality indices, specific foods, food groups, beverages (including alcohol), macronutrients (i.e., carbohydrate, fat, protein), and micronutrients (vitamins, minerals, antioxidants, polyphenols); comparison of this study-1) exposed group to any of the aforementioned factors versus the non-exposed group and 2) high intake of any of the aforementioned diet groups versus a low intake group; and primary outcome-incidence of colorectal cancer.

Studies were included that met the following criteria: 1) meta-analysis of prospective observational studies (i.e., cohort design) among adults with multivariable-adjusted summary risk estimates and corresponding 95% confidence intervals; and 2) investigated the association of dietary factor(s) with the incidence of CRC. Studies were excluded if they were primary studies, or if no summary estimate was reported (e.g., systematic reviews without meta-analysis). We also excluded (1) meta-analyses of studies with other study designs; and (2) meta-analyses that provided insufficient or inadequate data for quantitative synthesis. We also excluded meta-analyses published in languages other than English When more than one meta-analysis on the same research question was eligible, only one meta-analysis was selected for each exposure to avoid the inclusion of duplicate studies. In that case, the meta-analysis with the largest number of primary studies was selected. If more than one published meta-analysis on the same exposure included an equal number of studies, the one with the largest number of CRC cases was chosen. If more than one published meta-analysis fulfilled both criteria, the one with more comprehensive information on primary studies was selected.

Search strategy: We searched Medline, Embase and the Cochrane Library from database inception to September 2019. We also manually searched the cited references of the retrieved articles and reviews.

Data extraction: Data were extracted by two authors (Y.S. and T.Y.) and double-checked by a third author (S.V.). From each eligible article, we recorded the following: name of the first author, publication year, diet exposure, number of included studies, the total number of CRC cases and participants, type of comparison (e.g., high versus low), study-specific summary risk estimates (i.e., risk ratio (RR), odds ratio (OR), hazard ratio (HR), or incident rate ratio (IRR)) together with the corresponding confidence intervals, and estimates of publication bias. For each primary study included in the published meta-analysis, we noted whether relevant confounders were accounted for in adjusted summary estimates and reported. We communicated with authors to obtain data for evidence synthesis if it was not clearly reported in the published meta-analysis.

Definition of important dietary patterns:

Dietary pattern	Study	Exposure definition
Dietary calcium	Meng 2019[218]	The comparison of elemental intake of dietary calcium from each study included in the meta-analysis classified as highest categories (Q3, Q4, and Q5 [up to 2057 mg/day]) and the lowest categories (Q1 and Q2 [<228 mg/day]).
Dietary glycemic load	Reynolds 2019[182]	Based on WHO Nutrition Guidance Expert Advisory Group
Dietary glycemic load	Reynolds 2019[182]	Based on WHO Nutrition Guidance Expert Advisory Group
Healthy diet	Feng 2017[179]	High intakes of vegetables, fruits, whole grains, olive oil, fish, soy, poultry, and low-fat dairy
Heavy alcohol drinking	Fedirko 2011[201]	Consumption of ≥4 drinks/day (≥50 g/day of ethanol)
Light alcohol drinking	Fedirko 2011[201]	Consumption of ≤1 drink/day (≤12.5 g/day of ethanol)
Mediterranean diet	Schwingshackl 2017[178]	High consumption of plant-based foods, especially whole grain products, vegetables, fruits, nuts, and legumes with regular intake of fish and seafood. Eggs, red and processed meat as well as high-fat dairy products are consumed in low amounts
Moderate alcohol drinking	Fedirko 2011[201]	Consumption of 2–3 drinks/day (12.6–49.9 g/day of ethanol)
Western diet	Feng 2017	High consumption of red and/or processed meat, refined grains, sweets, high-fat dairy products, butter, potatoes and high-fat gravy, and low intake of fruits and vegetables
Non-vegetarian diet	Godos 2016[181]	Eating meat more than once per week
Pesco-vegetarian diet	Godos 2016[181]	Consumption of fish more than once per month in those following vegetarian diet
Semi-vegetarian diet	Godos 2016[181]	Low consumption of meat (more than once per month but less than once per week)
Supplemental calcium	Heine-Bröring 2015[216]	Use of calcium in supplement form. Mean level of intake:145 mg/day to 1,130 mg/day
Unhealthy diet	Grosso 2017[180]	High intakes of red and processed meat, sugary drinks and salty snacks, starchy foods, and refined carbohydrates
Vegetarian diet	Godos 2016[181]	Eating meat less than once per month

© 2021 Veettil SK et al. *JAMA Network Open.*

eTable 1. Search Strategy

No.	Search term	Search results		
		Embase 1974	CDSR	MEDLINE
1	exp Systematic Review/<br>*CDSR: systematic review.mp.	219179	7127	112213
2	systematic review.ti,ab.	170483	794	130108
3	exp Meta Analysis/<br>*CDSR: meta analysis.mp.	171779	8406	104669
4	meta-analysis.ti,ab.	174319	1843	127415
5	exp Colorectal Neoplasms/<br>*CDSR: Colorectal Neoplasms.mp.	27509	60	192528
6	exp Colonic Neoplasms/<br>*CDSR: Colonic Neoplasms.mp.	304714	12	72783
7	exp Rectal Neoplasms/<br>*CDSR: Rectal Neoplasms.mp.	240553	27	45779
8	exp Adenomatous Polyps/<br>*CDSR: Adenomatous Polyps.mp.	8868	19	7822
9	exp Adenocarcinoma/<br>*CDSR: Adenocarcinoma.mp.	208037	286	365492
10	exp Intestinal Polyps/<br>*CDSR: Intestinal Polyps.mp.	30058	3	14332
11	exp Colonic Polyps/<br>*CDSR: Colonic Polyps.mp.	19381	9	8126
12	colorectal cancer$.tw.	143433	280	92873
13	colorectal tumo$.tw.	9331	18	6648
14	colorectal neoplas$.tw.	5617	64	3574
15	colon cancer$.tw.	65894	103	44261
16	colon tumo$.tw.	6699	11	4907
17	colon neoplas$.tw.	599	5	380
18	colonic cancer$.tw.	3567	10	2808
19	colonic tumo$.tw.	2450	1	1759
20	colonic neoplas$.tw.	1679	19	1183
21	rectal cancer$.tw.	34633	70	21678
22	rectal tumo$.tw.	3399	11	2160
23	rectal neoplas$.tw.	560	31	370
24	rectum cancer$.tw.	929	18	520
25	rectum tumo$.tw.	171	11	92
26	rectum neoplas$.tw	23	0	13
27	polyps$.tw.	39414	110	268025
28	adenoma$.tw.	106700	129	80009
29	adenomatous$.tw.	19048	40	13997
30	exp Adenoma/<br>*CDSR: Adenoma.mp.	110273	79	98362
31	or/1-4	377143	9104	236427
32	or/5-30	667370	689	898844
33	31 and 32	12909	663	8899
34	Limit “33” to humans	1661<br>(exclude MEDLINE journals)	-	7954

© 2021 Veettil SK et al. *JAMA Network Open.*

eFigure. Study Flow Diagram

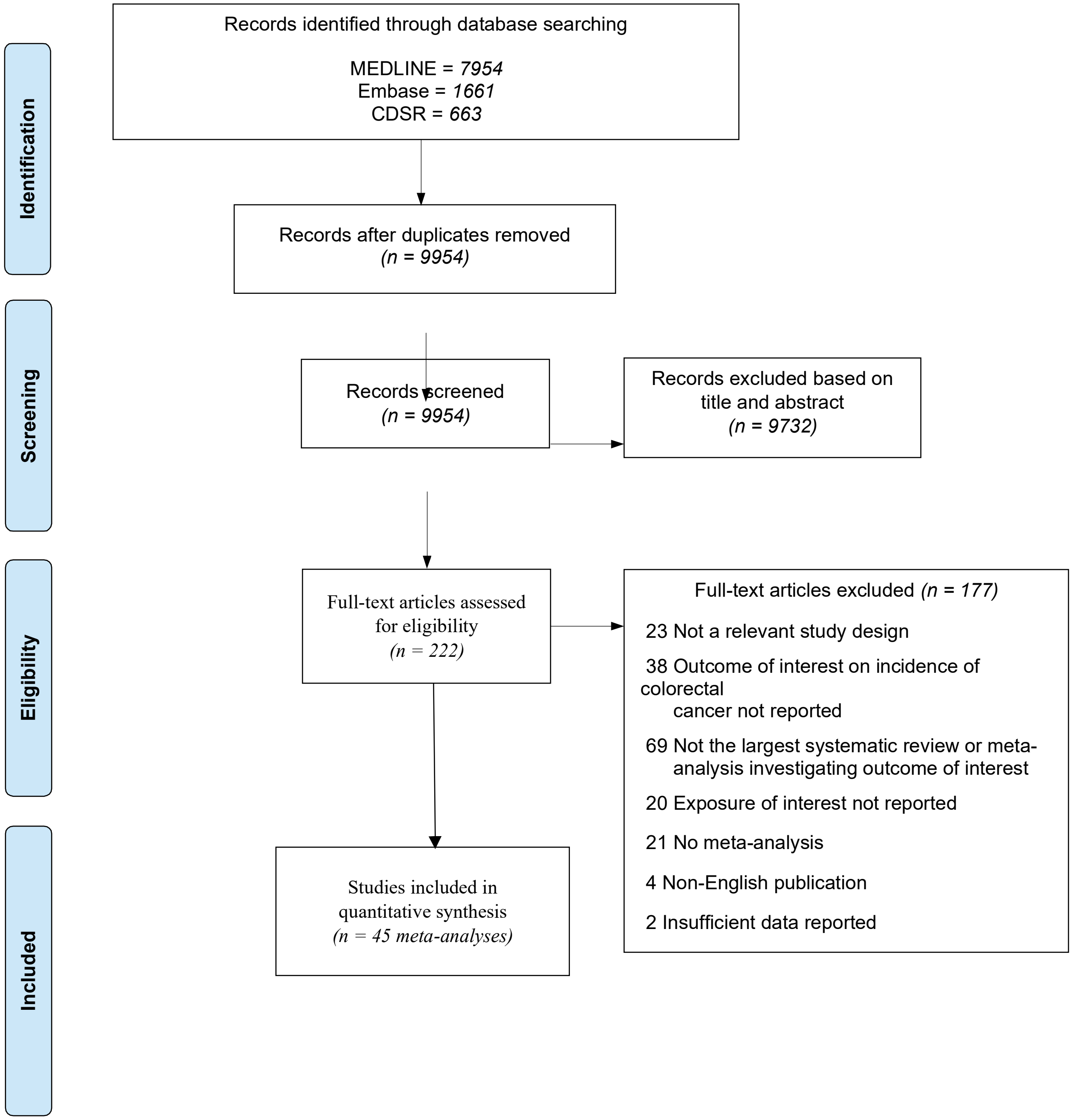

© 2021 Veettil SK et al. *JAMA Network Open.*

eTable 2. Excluded Studies

Reason	References
Outcome of interest on incidence of colorectal cancer not reported	*(1)(2)(3)(4)(5)(6)(7)(8)(9)(10)(11)(12)(13)(14)(15)(16)(17)(18)(19)(20)(21)(22)(23)(24)(25)(26)(27)(28)(29)(30)(31)(32)(33)(34)(35)(36)(37)(38)*
Not a relevant study design	*(39)(40)(41)(42)(43)(44)(45)(46)(47)(48)(49)(50)(51)(52)(53)(54)(55)(56)(57)(58)(59)(60)(61)*
No meta-analysis	*(62)(63)(64)(65)(66)(67)(68)(69)(70)(71)(72)(73)(74)(75)(76)(77)(78)(79)(80)(81)(82)*
Exposure of interest not reported	*(83)(84)(85)(86)(87)(88)(89)(90)(91)(92)(93)(94)(95)(96)(97)(98)(99)(100)(101)(102)*
Non-English publication	*(103)(104)(105)(106)*
Not the largest systematic review or meta-analysis investigating outcome of interest	*(107)(108)(109)(110)(111)(112)(113)(114)(115)(116)(117)(118)(119)(120)(121)(122)(123)(124)(125)(126)(127)(128)(129)(130)(131)(132)(133)(134)(135)(136)(137)(138)(139)(140)(141)(142)(143)(144)(145)(146)(147)(148)(149)(150)(151)(152)(153)(154)(155)(156)(157)(158)(159)(160)(161)(162)(163)(164)(165)(166)(167)(168)(169)(170)(171)(172)(173)(174)(175)*
Insufficient data reported	*(176)(177)*

© 2021 Veettil SK et al. *JAMA Network Open.*

eTable 3. Descriptive Characteristics of Included Meta-analyses

Exposure	Author; publication year	No. of primary studies	No. of participants	No. of cases	Duration of follow-up (range in years; mean in years)	Adjustment for confounding variables
**Dietary behaviours or diet quality indices**						
Adherence to Mediterranean diet	Schwingshackl 2017[178]	6	1410030	16102	5 - 26; 15.5	Age, sex, race/ethnicity, BMI, physical activity, educational level, socioeconomic status, smoking status, alcohol intake, family history of CRC, use of aspirin or other NSAIDs, colonoscopy, history of polyps, multivitamin use, energy intake, menopausal status, HRT use
Adherence to healthy diet	Feng 2017[179]	15	1182930	11537	1.7 - 14; 8.5	Age, sex, race/ethnicity, educational level, occupation, diabetes, BMI, smoking status, alcohol intake, physical activity, colorectal adenoma history, extent of colon resection, family history of CRC, energy intake, use of aspirin, use of HRT, multivitamin use, endoscopy
Adherence to unhealthy diet	Grosso 2017[180]	7	979243	9104	5 - 14; 9.5	Age, sex, race/ethnicity, BMI, energy intake, diabetes, educational level, smoking status, alcohol intake, occupation, physical activity, family history of CRC, use of aspirin or other NSAIDs, use of HRT
Adherence to Western diet	Feng 2017[179]	15	1182930	11537	1.7 - 14; 8.5	Age, sex, race/ethnicity, BMI, diabetes, smoking status, colorectal adenoma history, extent of colon resection, alcohol intake, educational level, occupation, physical activity, family history of CRC, energy intake, use of aspirin, menopausal status, use of HRT, multivitamin use, endoscopy
Adherence to alcohol drinking	Feng 2017[179]	9	718248	3965	5 - 16; 9.7	Age, sex, race/ethnicity, BMI, family history of CRC, educational level, smoking status, energy intake, physical activity, meat intake (red or processed meat), consumption of vegetables, fruit intake, use of aspirin, multivitamin use including dietary folate, total milk intake; intakes of fibre, fat, calcium
Vegetarian diet	Godos 2016[181]	3	149516	1506	7.3 - 20.3; 14.2	Age, sex, race/ethnicity, BMI, educational level, smoking status, alcohol intake, physical activity, family history of CRC, history of peptic ulcer, history of inflammatory bowel disease, treatment for diabetes mellitus within the past year, aspirin use, statin therapy, prior colonoscopy or flexible sigmoidoscopy, supplemental calcium consumption, supplemental vitamin D, energy intake, use of HRT, fibre intake
Pesco-vegetarian diet	Godos 2016[181]	3	149516	1506	7.3 - 20.3; 14.2	Age, sex, race/ethnicity, BMI, educational level, smoking status, alcohol intake, physical activity, family history of CRC, history of peptic ulcer, history of inflammatory bowel disease, treatment for diabetes mellitus within the past year, aspirin use, statin therapy,

© 2021 Veettil SK et al. *JAMA Network Open.*

						prior colonoscopy or flexible sigmoidoscopy, supplemental calcium consumption, supplemental vitamin D, energy intake, use of HRT, fibre intake
Semi-vegetarian diet	Godos 2016[181]	3	580175	4062	5 - 20.3; 10.9	Age, sex, race/ethnicity, total energy intake, smoking status, alcohol intake, BMI, physical activity, educational level, family history of CRC, history of peptic ulcer, history of inflammatory bowel disease, treatment for diabetes mellitus within the past year, aspirin use, statin therapy, prior colonoscopy or flexible sigmoidoscopy, supplemental calcium consumption, supplemental vitamin D, HRT use, intake of fibre
Dietary glycaemic index	Reynolds 2019[182]	10	941652	1121 9	6.9 - 15.7; 11.2	Age, sex, race/ethnicity, BMI, educational level, alcohol consumption, smoking status, BMI, use of NSAIDs, history of diabetes, colorectal screening, family history of any cancer, physical activity, energy intake, menopausal status, HRT use, multivitamin use, waist:hip ratio, calcium
Dietary glycaemic load	Reynolds 2019[182]	12	1181780	1421 4	6.9 - 16.5; 11.4	Age, sex, race/ethnicity, educational level, alcohol consumption, smoking status, BMI, history of diabetes, family history of any cancer, history of colorectal polyp, physical activity, colorectal screening, menopausal status, hormone therapy (OC or HRT), parity, energy intake, use of NSAIDs, multivitamin use including folic acid, waist:hip ratio, calcium, red meat
Eating frequency (3 vs <3 daily meals)	Liu 2014[183]	2	77641	550	5.8 - 10; 7.9	Age, sex, race/ethnicity, educational level, BMI, physical activity, smoking status, energy intake, calcium intake, vitamin D intake, alcohol intake, fruit intake, vegetable intake, red/processed meat intake, use of aspirin or other NSAIDs, family history of CRC, history of sigmoidoscopy/colonoscopy, total fat
Eating frequency (4 vs <3 daily meals)	Liu 2014[183]	3	112609	1133	5.8 - 14; 9.9	Age, sex, race/ethnicity, educational level, BMI, physical activity, smoking status, energy intake, calcium intake, alcohol intake, fruit intake, vegetable intake, meat intake (red or processed meat), use of aspirin or other NSAIDs, family history of CRC, history of sigmoidoscopy or colonoscopy, total fat, use of supplements containing antioxidants, vitamin intake (dietary folate, vitamin D), dietary approaches to stop hypertension (DASH) score
Eating frequency (≥5 vs <3 daily meals)	Liu 2014[183]	2	77641	550	5.8 - 10; 7.9	Age, sex, race/ethnicity, educational level, BMI, physical activity, smoking status, energy intake, calcium intake, vitamin D intake, alcohol intake, fruit intake, vegetable intake, meat intake (red or processed meat), use of aspirin or other NSAIDs, family history of CRC, history of sigmoidoscopy/ colonoscopy, total fat
**Food groups or foods**						

© 2021 Veettil SK et al. *JAMA Network Open.*

Red meat	Schwingshackl 2018[184]	21	2154027	21326	4.8 - 32; 11.3	Age, sex, race/ethnicity, energy intake, educational level, BMI, waist circumference, family history of CRC, history of colorectal polyps, diabetes, smoking status, alcohol intake, physical activity, screening and examinations, multivitamin use (vitamin B6, folate, vitamin D), use of aspirin or other NSAIDs, use of hormone therapy (OC or HRT), menopausal status, fruits, vegetables, grain foods including cereal, fibre intake, dietary calcium, dietary fat intake, tea consumption, intake of dried and salted fish
Processed meat	Schwingshackl 2018[184]	15	1910983	18646	4.8 - 20; 10.2	Age, sex, race/ethnicity, BMI, waist circumference, diabetes, family history of CRC, history of colorectal polyps, screening and examinations, educational level, smoking status, physical activity, energy intake, alcohol intake, vegetable intake, multivitamin use (vitamin B6, folate, vitamin D), use of aspirin or other NSAIDs, use of HRT, fruits, grain foods including cereal, intake of fibre, calcium, dietary fat intake, intake of dried and salted fish
Beef	Carr 2016[185]	4	654521	3937	4.8 - 13.4; 10.1	Age, sex, BMI, alcohol intake, smoking status, physical activity, energy intake, fibre intake, education, family history of cancer, consumption of fat, calcium, waist circumference, meat subtypes, HRT use, use of NSAIDs, diabetes and medication use, screening examinations, vitamin intake (vitamin B6, folate, vitamin D), dried and salted fish
Pork	Carr 2016[185]	4	654521	3937	4.8 - 13.4; 10.1	Age, sex, BMI, alcohol intake, smoking status, physical activity, energy intake, fibre intake, education, family history of cancer, consumption of fat, calcium, waist circumference, meat subtypes, HRT use, use of NSAIDs, diabetes and medication use, screening examinations, vitamin intake (vitamin B6, folate, vitamin D), dried and salted fish
Poultry	Carr 2016[185]	13	1492358	13716	4.8 - 32; 11.3	Age, sex, race/ethnicity, total energy intake, educational level, smoking status, BMI, alcohol intake, physical activity, calcium intake, occupation, geographical area; consumption of fruits, vegetables, cereals; use of NSAIDs, fat intake, family history of CRC or other cancers, history of colorectal polyps, HRT use, whole grain foods, fish intake including dried and salted fish, fibre intake, income, tea consumption, diabetes and medication use, vitamin intake (vitamin B6, folate, vitamin D), screening examinations, meat intake (red meat and other meat subtypes), waist circumference
Fish	Wu 2012[186]	18	1083264	6143	4.8 - 24; 11.5	Age, sex, race/ethnicity, BMI, family history of CRC or other cancers; history of stroke, hypertension, diabetes, myocardial infarction; smoking status, educational level, health screening, energy intake, parity; intakes of fish liver, fruits, vegetables, fibre, fats, sauces; multivitamin use including vitamin A, vitamin E, use of aspirin or other NSAIDs, physical activity, alcohol intake, meat intake including red meat and poultry, calcium intake, consumption of whole-grain foods and cereal, tea consumption, occupation, income, cholecystectomy

© 2021 Veettil SK et al. *JAMA Network Open.*

Fruits and vegetables	Aune 2011[187]	9	1522363	11543	4.3 - 16; 9.5	Age, sex, race/ethnicity, family history of CRC, history of colorectal polyps, menopausal status, HRT use, energy intake, BMI, physical activity, smoking status, alcohol consumption, meat intake (red and processed meat), fish intake, dietary fibre from cereal sources, educational level, dietary calcium, aspirin use, multivitamin use (including folate, vitamin D), consumption of dairy products, sigmoidoscopy
Fruits	Schwingshackl 2018[184]	17	1924385	19114	5 - 26; 11	Age, sex, race/ethnicity, diabetes, BMI, smoking status, alcohol intake, educational level, physical activity, energy intake, family history of CRC, multivitamin use (including folate, vitamin D), use of aspirin or other NSAIDs, intake of grains and cereal, meat intake including red and processed meat, calcium, screening and examinations, history of polyps or adenoma, menopausal status, HRT use, vegetable intake, intake of dairy products, fish intake, year of follow-up
Vegetables	Schwingshackl 2018[184]	17	1924385	19114	5 - 26; 11	Age, sex, race/ethnicity, diabetes, BMI, smoking status, alcohol intake, educational level, physical activity, energy intake, family history of CRC, multivitamin use (including folate, vitamin D), use of aspirin or other NSAIDs, intake of grains and cereal, meat intake including red and processed meat, calcium, screening and examinations, history of polyps or adenoma, menopausal status, HRT use, vegetable intake, intake of dairy products, fish intake, year of follow-up
Cruciferous vegetables	Wu 2013[188]	9	1117353	8021	4.3 - 20; 9	Age, race/ethnicity, family history of CRC, history of colorectal polyp, smoking status, BMI, physical activity, use of aspirin or other NSAIDs, multivitamin use (including folate, vitamin D), menopausal status, HRT use, energy intake, alcohol consumption, red meat, calcium, educational level, CRC screening, intake of fruits, consumption of grains
Broccoli	Wu 2013[188]	3	278338	2807	5 - 8.5; 6.9	Age, race/ethnicity, family history of CRC, history of colorectal polyp, BMI, smoking status, physical activity, use of aspirin or other NSAIDs, multivitamin use (including folate, vitamin D), HRT use, energy intake, alcohol consumption, red meat, calcium, intake of fruits, grain intake, educational level
Allium vegetables	Zhu 2014[189]	6	552180	5458	3.3 - 24; 14	Age, sex, energy intake, occupation, income, history of colorectal polyps, diabetes, BMI, physical activity, alcohol intake, smoking status, use of aspirin or other NSAIDs, HRT use, intake of calcium, fruits, vegetables, meat intake (including red or processed meat), family history of CRC or intestinal cancers, educational level, history of chronic intestinal disease or cholecystectomy, screening and examinations, fibre intake, multivitamin use (including folate, vitamin C, vitamin D)
Garlic	Chiavarini 2016[190]	5	330731	4141	5 - 24; 12.3	Age, sex, BMI, smoking status, family history, endoscopy, use of aspirin or other NSAIDs, physical activity, HRT use, meat intake (red or processed meat), alcohol consumption, calcium, energy intake, consumption of fruits, vegetable intake, previous polyps, vitamin intake (including folate, vitamin D)

© 2021 Veettil SK et al. *JAMA Network Open.*

Onion	Turati 2014 [191]	2	110524	1321	4 - 10; 7	Age, educational level, BMI, smoking status, alcohol intake, beta-carotene; history of cholecystectomy, chronic intestinal disease, colorectal polyps; family history of CRC, physical activity, energy intake, red meat, calcium, fibre, multivitamin use including vitamin C, aspirin use, sigmoidoscopy, HRT use
Legumes	Zhu 2015[192]	13	1782607	11351	5 - 16; 8.9	Age, sex, BMI, energy intake, history of colorectal polyps, physical activity, family history of CRC, smoking status, alcohol consumption, use of aspirin or other NSAIDs, sigmoidoscopy, menopausal status, HRT use, multivitamin use (including folate, vitamin D), meat intake (red or processed meat, pork), educational level; intakes of fruits, grains, calcium, dairy products, vegetables, fish, fibre; coffee intake, income, diabetes
Nuts	Schwingshackl 2018[184]	6	1152672	7283	4.8 - 30; 13.1	Age, sex, BMI, alcohol intake, family history of CRC, physical activity, aspirin use, history of colorectal polyps, smoking status, energy intake, multivitamin use, fruit intake, intake of dietary fibre, HRT use, screening and examinations, history of ulcerative colitis, cholesterol and triglyceride
Soy products	Lu 2017[193]	5	281425	5615	8 - 13; 10	Age, sex, race/ethnicity, educational level, household income, physical activity, BMI, menopausal status, HRT use, family history of CRC, energy intake; intakes of fruits, vegetables, non-soy calcium, non-soy fibre; vitamin use (non-soy folic acid, vitamin D), dairy products, meat intake including red meat, fish intake, smoking status, alcohol consumption, diabetes, coffee intake
Whole grains	Schwingshackl 2018[184]	9	970927	9223	5 - 26; 14.1	Age, sex, BMI, smoking status, educational level, alcohol consumption, fibres from foods other than whole-grain bread, calcium, energy intake, HRT use, family history of CRC, physical activity, aspirin use, colonoscopy, history of polyps, multivitamin use including folate; intakes of saturated fats, fruits, vegetables; meat intake (red or processed meat)
Refined grains	Schwingshackl 2018[184]	2	72431	900	14 - 14.8; 14.4	Age, race/ethnicity, BMI, physical activity, smoking status, educational level, energy intake; intakes of saturated fat, calcium, red meat, fruits, vegetables; family history of CRC, endoscopy, aspirin use
Eggs	Schwingshackl 2018[184]	3	94181	598	7.4 - 32; 18.8	Age, sex, BMI, educational level, occupation, smoking status, geographic region, energy intake; intakes of vegetables, fruits, cereals; tea consumption, use of NSAIDs, fibre intake, alcohol consumption
Dairy products	Schwingshackl 2018[184]	17	1629366	16910	3.3 - 26; 11.7	Age, sex, race/ethnicity, occupation, geographical area, diabetes at baseline, smoking status, BMI, alcohol intake, educational level, physical activity, family history of CRC, energy intake, use of aspirin or other NSAIDs, colonoscopy, history of polyps, multivitamin use (including vitamin B6, folate, vitamin D), history of gallbladder surgery, intake of fat, dietary fibre, meat intake (red or processed meat), intake of fruits, vegetable intake, tea consumption, menopausal status, use of hormone therapy (OC or HRT)
Cheese	Aune 2012[194]	7	234759	2121	3.3 - 19.6; 11.2	Age, sex, BMI, occupation, smoking status, geographical area, energy intake, family history of CRC, fat intake, dietary fibre, gallbladder surgery, alcohol intake, physical activity, educational level, red meat, history of colon polyps, multivitamin use (including vitamin B6, folate), HRT use, menopausal status, diabetes, aspirin use, intake of fruits, vegetable intake

© 2021 Veettil SK et al. *JAMA Network Open.*

Yogurt	Zhang 2019[195]	5	698366	5432	3.3 - 12; 7.7	Age, sex, family history of CRC or other cancers, previous polyp, screening, smoking status, alcohol intake, aspirin use, physical activity, BMI, meat intake (including red or processed meat), fat intake, dietary fibre, gallbladder surgery, energy intake, educational level, menopausal status, hormone therapy (OC or HRT), dietary calcium, simple sugars, history of diabetes, vegetables, fruits, nuts and legumes, cereals, fish
**Beverages**						
Tea	Chen 2017[196]	15	1208316	14582	1 - 15; 8.6	Age, sex, race/ethnicity, family history of CRC, BMI, intake of fibre, coffee intake, alcohol intake, diabetes, educational level, smoking status, physical activity, intake of fruits, vegetable intake, calcium, energy intake, meat intake (including red meat and pork), radiation exposure, use of aspirin or other NSAIDs, fat intake, vitamin supplement intake, menopausal status, HRT use, household income, history of colorectal polyps and chronic ulcerative colitis, occupation, colorectal screening
Green tea	Wang 2012[197]	5	352275	1675	6 - 15; 9.2	Age, sex, family history of CRC, smoking status, alcohol intake, BMI, meat consumption including red meat, intake of black tea, intake of fruits, vegetable intake, coffee consumption, radiation exposure, menopausal status, use of NSAIDs, vitamin supplement use, history of colorectal polyps and chronic ulcerative colitis, energy intake
Black tea	Sun 2006[198]	6	274975	2847	8 - 20; 13	Age, sex, race/ethnicity, educational level, family history of CRC, history of sigmoidoscopy or colonoscopy, BMI, smoking status, physical activity, aspirin use, vitamin supplement intake, alcohol consumption, red meat consumption, total energy intake, menopausal status, HRT use; intakes of fat, fibre, calcium; fruit intake, vegetable intake, waist/hip circumference ratio
Coffee	Gan 2017[199]	19	2046575	22629	4.5 - 18; 10.1	Age, sex, race/ethnicity, BMI, smoking status, alcohol intake, educational level, serum cholesterol, physical activity, calcium intake, tea consumption, energy intake, family history of CRC, use of aspirin or other NSAIDs, colorectal screening, vitamin intake (including vitamin B6, folic acid, vitamin C, vitamin D), fat intake, fibre intake, menopausal status, HRT use, diabetes, fruits, vegetables, meat intake including red or processed meat and pork, number of pregnancies and deliveries, age at menarche, age at first delivery, intake of dairy products
Non-fermented milk	Ralston 2014[200]	14	892569	5076	5 - 24; 11.3	Age, sex, race/ethnicity, occupation, smoking status, geographical area, BMI, total energy intake, family history of CRC, previous intestinal polyp, screening, use of aspirin or other NSAIDs, physical activity, saturated fat, dietary fibre intake, alcohol intake, red meat consumption, educational level, history of diabetes, fruits, vegetables, multivitamin use

© 2021 Veettil SK et al. *JAMA Network Open.*

						(including folic acid, vitamin C, vitamin D), income, tea consumption, menopausal status, HRT use
Fermented milk	Ralston 2014[200]	7	328750	1876	6 - 24; 10.4	Age, sex, occupation, smoking status, geographical area, BMI, total energy intake, family history of CRC, previous intestinal polyp, screening, aspirin use, physical activity, saturated fat, dietary fibre intake, alcohol intake, red meat consumption, educational level, history of diabetes, fruits, vegetables, multivitamin use (including folic acid, vitamin C, vitamin D), menopausal status, HRT use
Alcohol (Moderate)	Fedirko 2011[201]	22	2798092	1912 3	3.3 - 20; 10.2	Age, sex, race/ethnicity, smoking status, BMI, coffee intake, educational level, cholesterol, history of gall bladder surgery, energy intake; intakes of fats, protein, dietary fibre; family history of CRC, physical activity, history of polyps, multivitamin use (including folate and vitamin D), meat intake (including poultry/non-poultry meat, processed meat), seafood intake, calcium, occupation, intake of vegetables, fruit intake, diabetes, menopausal status, use of hormone therapy (OC or HRT), socioeconomic status, aspirin use, screening and examinations
Alcohol (Heavy)	Fedirko 2011[201]	7	738539	5078	4.8 - 16; 9.9	Age, sex, family history of CRC, BMI, smoking status, physical activity, educational level, sedentary work, consumption of vegetables, meat consumption (including red or processed meat), fruit intake, energy intake, aspirin use, screening and examinations, intake of calcium, multivitamins (including folate, vitamin D)
Beer	Zhang 2015[202]	7	805177	5149	4 - 24; 10.4	Age, sex, race/ethnicity, family history of CRC, smoking status, coffee intake, total serum cholesterol, educational level, BMI, non-contraceptive oestrogen use, physical activity, history of colorectal polyps, energy intake, intake of fats, dietary fibre, calcium, other types of alcohol
Wine	Xu 2019[203]	9	973286	7511	4 - 17.8; 9.1	Age, sex, race/ethnicity, smoking status, coffee intake, total serum cholesterol, educational level, BMI, non-contraceptive oestrogen use, history of colorectal polyps, physical activity, intake of other types of alcohol, meat consumption including poultry, seafood consumption, multivitamin use, energy intake, family history of CRC, intake of fat, dietary fibre, calcium, fruit intake, vegetable intake, diabetes
Wine (Light to moderate)	Xu 2019[203]	4	676331	4559	5.3 - 14.7; 9.9	Age, sex, race/ethnicity, smoking status, BMI, intake of other types of alcohol, physical activity, educational level, energy intake, family history of CRC, intake of fat, dietary fibre, calcium, fruit intake, vegetable intake, meat intake, multivitamin use, diabetes

© 2021 Veettil SK et al. *JAMA Network Open.*

Wine (Heavy)	Xu 2019[203]	5	686749	4670	5.3 - 14.7; 9.9	Age, sex, race/ethnicity, smoking status, BMI, intake of other types of alcohol, educational level, meat consumption including poultry, seafood consumption, multivitamin use, history of colonic polyps, physical activity, energy intake, family history of CRC, intake of fat, dietary fibre, calcium, fruit intake, vegetable intake, diabetes
**Macronutrients**						
Total dietary fat	Liu 2011[204]	13	459910	3635	3 - 32; 11.5	Age, sex, BMI, energy intake, parity, fibre intake, smoking status, educational level, alcohol intake, physical activity, calcium intake, geographical area, occupation; consumption of fruits, vegetables, cereals; family history of CRC, history of colorectal polyps, hormone therapy, diabetes, vitamin use (including vitamin A, vitamin E)
Saturated fatty acids	Liu 2011[204]	12	451956	3182	3 - 32; 9.9	Age, sex, total energy intake, parity, fibre intake, BMI, smoking status, education, alcohol consumption, physical activity, calcium intake, geographical area, occupation; intakes of fruits, vegetables, cereals; family history of CRC, history of colorectal polyps, hormone therapy, diabetes, vitamin use (vitamin A, vitamin E)
Monounsaturated fatty acids	Liu 2011[204]	11	399687	3048	3 - 32; 11.9	Age, sex, total energy intake, parity, fibre intake, BMI, smoking status, education, alcohol consumption, physical activity, calcium intake, geographical area, occupation; intakes of fruits, vegetables, cereals; family history of CRC, history of colorectal polyps, hormone therapy, diabetes, vitamin use (vitamin A, vitamin E)
Polyunsaturated fatty acids	Kim 2018[205]	14	933712	10036	3.3 - 32; 13	Age, sex, race/ethnicity, dietary fibre intake, Dutch Healthy Diet index, energy intake, BMI, educational level, family history of CRC, screening and examinations, use of aspirin or other NSAIDs, intake of alcohol, smoking status, physical activity, hormone therapy, calcium, multivitamin use (including folate, vitamin D), fruit intake, vegetable intake, meat intake (red or processed meat), history of polyps, cereal intake, cardiovascular disease, memory loss, use of cholesterol-lowering drugs, omega-6 (linoleic + arachidonic) intake, menopausal status, past history of or medication use for diabetes, intake of low-fat dairy products, geographical area, occupation
Total n-3 polyunsaturated fatty acids	Chen 2015[206]	8	579427	6807	4.8 - 22; 10.8	Age, sex, parity, total energy intake, BMI, smoking status, alcohol intake, educational level, physical activity, calcium intake, meat intake (red or processed meat), dietary fibre, intake of fat (saturated fat, monounsaturated fat, n-6 PUFA), diabetes, family history of CRC, menopausal status, use of hormone therapy, multivitamin use (including vitamin A, folate, vitamin C, vitamin D, vitamin E), use of aspirin or other NSAIDs, screening and examinations, low-fat dairy products, fruits, vegetables

© 2021 Veettil SK et al. *JAMA Network Open.*

Marine n-3 polyunsaturated fatty acids	Chen 2015[206]	10	666713	7813	6 - 22; 11.3	Age, sex, race/ethnicity, BMI, educational level, alcohol intake, energy intake, dietary fibre, calcium, fat intake (saturated fat, monounsaturated fat, n-6 PUFA), family history of CRC, history of colorectal polyps, physical activity, smoking status, hormone therapy, multivitamin use (including folate, vitamin C, vitamin D), diabetes, use of aspirin or other NSAIDs, menopausal status, low-fat dairy products, fruits, vegetables, cardiovascular disease, memory loss, use of cholesterol-lowering drugs, screening and examinations, meat intake (red or processed meat)
Cholesterol	Liu 2011[204]	7	261260	1163	3 - 32; 10.6	Age, sex, total energy intake, parity, educational level, BMI, smoking status, alcohol consumption, physical activity, calcium intake, family history of CRC, history of colorectal polyps, hormone therapy, occupation, geographical area; consumption of vegetables, fruits, cereals; vitamin intake (vitamin A, vitamin E)
Carbohydrate	Aune 2012[207]	11	806647	9799	7.1 - 22; 12.6	Age, race/ethnicity, educational level, income, BMI, physical activity, family history of CRC, hormone therapy (OC or HRT), total energy intake, colorectal polyps, smoking status, use of aspirin or other NSAIDs, multivitamin use (including folate, vitamin D), alcohol intake, meat intake (red or processed meat), calcium, dietary fibre, diabetes, colorectal screening, magnesium, total fat, parity
Sucrose	Aune 2012[207]	5	831687	9216	7.2 - 20; 12	Age, race/ethnicity, BMI, family history of CRC or other cancers, smoking status, educational level, physical activity, total energy intake, alcohol intake, colorectal polyps, use of aspirin or other NSAIDs, multivitamin use (including folate, vitamin D), hormone therapy (OC or HRT), meat intake (red or processed meat), calcium, dietary fibre, diabetes, prior endoscopy screening, total fat
Fructose	Aune 2012[207]	4	640683	6837	7.2 - 20; 12.8	Age, race/ethnicity, BMI, family history of CRC or other cancers, smoking status, educational level, physical activity, total energy intake, alcohol intake, multivitamin use (including folate, vitamin D), diabetes, prior endoscopy screening, use of aspirin or other NSAIDs, calcium, meat intake (red or processed meat), hormone therapy (OC or HRT), total fibre, total fat
Dietary protein	Lai 2017[208]	3	207068	680	3.3 - 12; 7.8	Age, sex, energy intake, dietary fibre intake, supplement intake, smoking status, BMI, alcohol intake, educational level, physical activity, calcium intake (except for milk protein and milk products)
Total dietary fibre	Reynolds 2019 [182]	21	2259486	2300 4	4.5 - 17; 10	Age, sex, race/ethnicity, physical activity, smoking status, meat intake (red and processed meat), total energy intake, calcium, BMI, educational level, alcohol intake, family history, colorectal polyp, use of multivitamin (including folate, vitamin C, vitamin D), aspirin or other anti-inflammatory use, hormone therapy (OC or HRT), menopause, colonoscopy, dietary assessment, fat
Cereal fibre	Aune 2011[209]	7	1471756	9487	4.5 - 16; 9.5	Age, sex, race/ethnicity, family history of CRC, history of colorectal polyps, smoking status, BMI, physical activity, use of aspirin or other NSAIDs, multivitamin use (including folate, vitamin D), meat intake (red and processed meat), menopausal status, HRT use, alcohol intake, calcium intake, energy intake, educational level, sigmoidoscopy or colonoscopy, glycaemic load, consumption of dairy products

© 2021 Veettil SK et al. *JAMA Network Open.*

Fruit fibre	Aune 2011[209]	8	1514871	9930	4.5 - 16; 9.3	Age, sex, race/ethnicity, family history of CRC, history of colorectal polyps, smoking status, BMI, physical activity, use of aspirin or other NSAIDs, multivitamin use (including folate, vitamin D), alcohol intake, menopausal status, HRT use, meat intake (red or processed meat, pork), calcium intake, energy intake, education, sigmoidoscopy or colonoscopy, glycaemic load, consumption of dairy products
Vegetable fibre	Aune 2011[209]	8	1514871	9930	4.5 - 16; 9.3	Age, sex, race/ethnicity, family history of CRC, history of colorectal polyps, smoking status, BMI, physical activity, use of aspirin or other NSAIDs, multivitamin use (including folate, vitamin D), alcohol intake, menopausal status, HRT use, meat intake (red or processed meat, pork), calcium intake, energy intake, education, sigmoidoscopy or colonoscopy, glycaemic load, consumption of dairy products
Legume fibre	Reynolds 2019[182]	4	1104339	5651	5 - 12.1; 8.3	Age, sex, physical activity, smoking status, menopause, HRT use, meat intake (red or processed meat), folate, calcium, energy intake, alcohol intake, educational level, BMI, family history, history of colon polyps, aspirin use
Soluble fibre	Reynolds 2019[182]	3	204243	2580	7.6 - 11.7; 9.1	Age, sex, energy intake, BMI, educational level, family history, colonoscopy, anti-inflammatory use, consumption of alcohol, smoking status, physical activity, HRT use, calcium, red meat, vitamin intake (folate, vitamin D)
Insoluble fibre	Reynolds 2019[182]	3	204243	2580	7.6 -11.7; 9.1	Age, sex, energy intake, BMI, educational level, family history, colonoscopy, anti-inflammatory use, consumption of alcohol, smoking status, physical activity, HRT use, calcium, red meat, vitamin intake (folate, vitamin D)
**Micronutrients**						
Flavonoids	Bo 2016[210]	6	188135	6609	6.1 - 28; 14.2	Age, race/ethnicity, BMI, occupation, geographic area, family history of CRC, history of colorectal polyps, prior sigmoidoscopy screening, physical activity, smoking status, alcohol consumption, intake of meat, intake of fruits, vegetables, intake of fibre, total fat, total energy, calcium, vitamin use (folate, vitamin C, vitamin E), aspirin use, HRT use
Flavonols	Chang 2018[211]	5	729461	9720	11 - 28; 18.9	Age, sex, geographic area, occupation, smoking, physical activity, education, BMI, history of CRC, history of endoscopy, alcohol consumption, total energy intake, total fat, intake of meat, fibre, calcium intake, menopausal status, hormone therapy (OC or HRT), regular aspirin use, vitamin intake (vitamin C, vitamin D, vitamin E)
Quercetin	Grosso 2017[212]	2	117266	463	10 - 28; 19	Age, sex, geographic area, occupation, smoking status, BMI, family history of CRC, history of colorectal polyps, prior sigmoidoscopy screening, physical activity, alcohol consumption, meat intake, total energy intake, total calcium intake, total fibre intake, aspirin use, vitamin intake, HRT use
Kaempferol	Grosso 2017[212]	2	117266	472	10 - 28; 19	Age, sex, geographic area, occupation, smoking status, BMI, family history of CRC, history of colorectal polyps, prior sigmoidoscopy screening, physical activity, alcohol consumption, meat intake, total energy intake, total calcium intake, total fibre intake, aspirin use, vitamin intake, HRT use

© 2021 Veettil SK et al. *JAMA Network Open.*

Myricetin	Grosso 2017[212]	2	117266	466	10 - 28; 19	Age, sex, geographic area, occupation, smoking status, BMI, family history of CRC, history of colorectal polyps, prior sigmoidoscopy screening, physical activity, alcohol consumption, meat intake, total energy intake, total calcium intake, total fibre intake, aspirin use, vitamin intake, HRT use
Flavones	Chang 2018[211]	3	598744	7091	11 - 26; 17.7	Age, sex, history of CRC, history of endoscopy, smoking status, physical activity, education, BMI, alcohol consumption, total energy, total fat, intake of meat, fibre intake, calcium intake, menopausal status, hormone therapy (OC or HRT), regular aspirin use, vitamin intake (vitamin C, vitamin D, vitamin E)
Flavanones	Chang 2018[211]	4	608609	7181	11 - 28; 20.3	Age, sex, geographic area, occupation, history of CRC, history of endoscopy, smoking, physical activity, education, BMI, alcohol consumption, total energy, total fat, intake of meat, fibre intake, calcium intake, menopausal status, hormone therapy (OC or HRT), regular aspirin use, vitamin intake (vitamin C, vitamin D, vitamin E)
Flavan-3-ols	Chang 2018[211]	4	719596	9576	11 - 26; 16.6	Age, sex, family history of CRC, history of CRC, history of endoscopy, smoking, physical activity, education, BMI, alcohol consumption, total energy, total fat, meat intake, fibre intake, calcium intake, menopausal status, hormone therapy (OC or HRT), regular aspirin use, vitamin intake (vitamin C, vitamin D, vitamin E)
Catechin	Grosso 2017[212]	2	155503	3249	13 - 13.3; 13.2	Age, occupation, BMI, family history of CRC, waist-to-hip ratio, physical activity, smoking, alcohol intake, total fruit intake, vegetable consumption, meat intake, total energy intake
Flavanols	He 2016[213]	3	242284	5059	13.3 - 26; 18.5	Age, BMI, family history of CRC, history of endoscopy, alcohol consumption, physical activity, smoking, fibre intake, meat intake, total energy intake, use of NSAIDs, dietary supplement (calcium, *n*-3 polyunsaturated fatty acids, manganum, riboflavin, vitamin C, vitamin E, folate)
Anthocyanins	He 2016[213]	2	121432	2574	16.2 - 26; 21.1	Age, BMI, family history of CRC, history of endoscopy, alcohol consumption, physical activity, smoking, fibre intake, meat intake, total energy intake, use of NSAIDs, dietary supplement (calcium, *n*-3 polyunsaturated fatty acids, manganum, riboflavin, folate, vitamin C, vitamin E)
Anthocyanidins	Chang 2018[212]	3	598744	7091	11 - 26; 17.7	Age, sex, history of CRC, history of endoscopy, smoking, physical activity, education, BMI, alcohol consumption, total energy, total fat, intake of meat, fibre intake, calcium intake, menopausal status, hormone therapy (OC or HRT), regular aspirin use, vitamin intake (vitamin C, vitamin D, vitamin E)
Phyto-oestrogens	Jiang 2017[214]	5	275443	2485	6.4 - 19; 10.2	Age, sex, household income, dialect group, diabetes at baseline, smoking, BMI, alcohol intake, education, physical activity, family history of CRC, daily energy intake, menopausal status, average intakes of fruits, vegetables, meat intake, fibre intake, non-soya calcium, fats, vitamin intake (non-soya folic acid, vitamin D), use of NSAIDs, hormone therapy
Isoflavones	Grosso 2017[212]	5	292616	2587	6.4 - 19; 10.2	Age, education, alcohol intake, smoking status, BMI, physical activity, household income, family history of CRC, history of diabetes mellitus, total energy intake, intakes of fruits, vegetables, meat, non-soy calcium, fibre, coffee intake, fats, dairy products, individual phytoestrogens, menopausal status, HRT use, vitamin intake (folic acid, vitamin D)

© 2021 Veettil SK et al. *JAMA Network Open.*

Combined carotenoids	Panic 2017[215]	2	196383	2673	8.2 - 11; 9.6	Age, education level, smoking status, BMI, physical activity, family history of CRC, history of intestinal polyps, alcohol intake, total energy intake, total fat intake, meat intake, dietary fibre intake, total calcium intake, hormone therapy (HRT), use of NSAIDs, vitamin intake (folate, vitamin D)
Alpha-carotene	Panic 2017[215]	3	223334	2857	8 - 11; 9.1	Age, education level, smoking status, BMI, physical activity, family history of CRC, history of intestinal polyps, serum cholesterol, alcohol consumption, total energy intake, total fat intake, meat intake, dietary fibre intake, total calcium intake, HRT use, use of NSAIDs, vitamin intake (folate, vitamin D)
Beta-carotene	Panic 2017[215]	4	279666	3605	8 - 11; 9.5	Age, education level, smoking status, BMI, physical activity, family history of CRC, history of intestinal polyps, serum cholesterol, alcohol intake, total energy intake, meat intake, dietary fibre intake, total calcium intake, hormone therapy (HRT), use of NSAIDs, vitamin intake (folate, vitamin D)
Lycopene	Panic 2017[215]	3	223334	2857	8 - 11; 9.1	Age, education level, smoking status, BMI, physical activity, family history of CRC, history of intestinal polyps, serum cholesterol, alcohol consumption, total energy intake, total fat intake, meat intake, dietary fibre intake, total calcium intake, hormone therapy (HRT), use of NSAIDs, multivitamin use (folate, vitamin D)
Beta-cryptoxanthin	Panic 2017[215]	2	196383	2673	8.2 - 11; 9.6	Age, education level, smoking status, BMI, physical activity, family history of CRC, history of intestinal polyps, alcohol intake, total energy intake, total fat intake, meat intake, dietary fibre intake, total calcium intake, hormone therapy (HRT), use of NSAIDs, vitamin intake (folate, vitamin D)
Lutein and zeaxanthin	Panic 2017[215]	3	223334	2857	8 - 11; 9.1	Age, education level, smoking status, BMI, physical activity, family history of CRC, history of intestinal polyps, serum cholesterol, alcohol consumption, total energy intake, total fat intake, meat intake, dietary fibre intake, total calcium intake, HRT use, use of NSAIDs, vitamin intake (folate, vitamin D)
Multivitamin	Heine-Bröring 2015[216]	7	670513	8737	5 - 24; 12.7	Age, sex, race/ethnicity, education, family history of CRC, BMI, physical activity, smoking status, alcohol consumption, energy intake, dietary factor, use of NSAIDs, menopausal status
Multivitamin	Liu 2015[217]	5	522507	3584	5 - 16; 9	Age, race/ethnicity, BMI, education level, alcohol consumption, smoking status, physical activity, family history of CRC, meat intake, total energy intake, fruit intake, vegetable intake, intake of saturated fat, dietary fibre, vitamin intake (vitamin B6, folate, vitamin C, vitamin E), calcium, menopausal status, hormone therapy, aspirin use
Vitamin A	Heine-Bröring 2015[216]	2	46796	443	8 - 10; 9	Age, smoking status, energy intake
Vitamin A	Liu 2015[217]	6	208536	1206	4.5 - 10; 7	Age, smoking status, education level, physical activity, alcohol consumption, family history of CRC, menopausal status, total energy intake, intake of meat, hormone therapy (HRT)
Vitamin B2	Liu 2015[217]	5	392184	4939	5.74 - 13.3; 10	Age, educational level, BMI, household income, smoking status, alcohol intake, physical activity, family history of CRC, diabetes history, energy intake, vegetables, fruits, meat, calcium, fibre, iron, fats, menopausal status, hormone therapy (HRT), use of NSAIDs

© 2021 Veettil SK et al. *JAMA Network Open.*

Vitamin B3	Liu 2015[217]	2	146175	677	5.7	Age, educational level, BMI, household income, smoking status, alcohol intake, physical activity, family history of CRC, diabetes history, energy intake, vegetables, fruits, meat, calcium, fibre, iron, fats, menopausal status, hormone therapy (HRT), use of NSAIDs
Vitamin B6	Liu 2015[217]	11	809074	9591	5.8 - 22; 12.8	Age, sex, BMI, education, household income, smoking status, alcohol consumption, physical activity, family history of CRC, diabetes history, total intake of energy, intake of vegetables, fruits, meat, fats, iron, calcium, use of aspirin or other NSAIDs, vitamin use (vitamin B, vitamin E), hormone therapy (HRT), menopausal status
Folic acid	Liu 2015[217]	19	1988974	2171 2	5 - 20; 14.6	Age, sex, BMI, education, household income, smoking status, alcohol consumption, physical activity, family history of CRC, diabetes history, past medical history of colonoscopy, total intake of energy, intake of vegetables, fibre, fruits, meat, fats, iron, calcium, use of aspirin or other NSAIDs, use of vitamin (vitamin B, vitamin C, vitamin E), hormone therapy (HRT) , menopausal status
Vitamin B12	Liu 2015[217]	5	321038	3554	5.8 - 13; 9.9	Age, BMI, race/ethnicity, past medical history of colonoscopy, smoking status, physical activity, alcohol consumption, diabetes history, energy intake, intake of vegetables, fruits, meat, calcium, use of NSAIDs, vitamin use, hormone therapy (HRT), menopausal status
Vitamin C	Heine-Bröring 2015[216]	3	103128	1191	8 - 10.6; 9.5	Age, BMI, educational level, physical activity, smoking status, alcohol consumption, energy intake, dietary factors, menopausal status
Vitamin C	Liu 2015[217]	9	1003710	6542	4.5 - 14; 8.3	Age, BMI, occupation, education level, smoking level, alcohol consumption, physical activity, family history of CRC, energy intake, intake of fibre, vegetables, multivitamin intake, menopausal status, serum cholesterol concentration, hormone therapy (HRT), use of aspirin
Vitamin D	Heine-Bröring 2015[216]	5	362610	3147	7.3 - 24; 12.7	Age, race/ethnicity, sex, family history of CRC, BMI, physical activity, smoking status, alcohol intake, energy intake, dietary factors, menopausal status, use of NSAIDs
Vitamin D	Liu 2015[217]	14	869730	1856 4	5 - 24; 9.5	Age, sex, geographical area, occupation, race/ethnicity, BMI, physical activity, smoking status, educational level, total energy intake, fruits, meat, vegetables, alcohol intake, fat intake, dietary fibre intake, calcium intake, CRC screening, menopausal status, family history of CRC, history of intestinal polyps, use of aspirin or other NSAIDs, hormone therapy (HRT), vitamin use
Vitamin E	Heine-Bröring 2015[216]	4	191126	2216	8 - 18; 10.9	Age, BMI, family history of CRC, physical activity, smoking status, educational level, energy intake, alcohol consumption, dietary factors, menopausal status, use of NSAIDs
Vitamin E	Liu 2015[217]	10	1139052	7567	4.5 - 10.6; 8.1	Age, sex, BMI, educational level, alcohol intake, smoking status, physical activity, family history of CRC, history of colorectal polyps, serum cholesterol, energy intake, intake of meat, vegetable consumption, intake of fibre, vitamin intake, hormone therapy (HRT), menopausal status, aspirin use

© 2021 Veettil SK et al. *JAMA Network Open.*

Dietary calcium	Meng 2019[218]	8	1449526	1364 0	7 - 16.4; 10	Age, sex, race/ethnicity, BMI, waist:hip ratio, education, smoking status, tea intake, alcohol consumption, physical activity, family history of CRC or other cancers, history of intestinal polyps, CRC screening, diabetes, total energy intake, fat intake, intake of meat, intake of fruits, vegetables, whole grains, fibre, intake of phosphorus, retinol, sodium, potassium, zinc, use of calcium supplement, multivitamin use, use of ginseng, menopausal status, hormone therapy (OC or HRT), statin use, use of aspirin or other NSAIDs
Supplemental calcium	Heine-Bröring 2015[216]	7	1064458	9862	3.3 - 16; 8.4	Age, race/ethnicity, BMI, physical activity, educational level, family history of CRC, smoking status, alcohol consumption, energy intake, dietary factors, menopausal status, use of NSAIDs
Supplemental calcium	Heine-Bröring 2015[216]	6	929116	8837	5 - 10; 8	Age, race/ethnicity, BMI, physical activity, educational level, family history of CRC, smoking status, alcohol consumption, energy intake, dietary factors, menopausal status, use of NSAIDs
Heme iron	Qiao 2013[219]	6	646901	8022	7.2 - 22; 15.7	Age, sex, BMI, education level, physical activity, smoking status, alcohol consumption, family history of CRC, history of endoscopy, diabetes, intake of total energy, fat, calcium, fibre, zinc, magnesium, hormone therapy (HRT), regular aspirin use, menopausal status, vitamin intake
Magnesium	Chen 2012[220]	7	336463	7435	7.9 - 28; 15.9	Age, sex, BMI, screening, waist:hip ratio, education, diabetes, physical activity, smoking status, alcohol intake, family history of CRC, history of colon polyps, history of CRC, energy intake, fat intake, meat intake, fruits, vegetables, whole grain, calcium, zinc, fibre, menopausal status, hormone therapy (HRT), vitamin intake (vitamin A, vitamin B6, folate, vitamin C, vitamin D, vitamin E, vitamin K2), aspirin use
Zinc	Li 2014[221]	5	350507	5676	9.5 - 22; 15.7	Age, BMI, geographic region, alcohol intake, physical activity, education, smoking status, diabetes, history of CRC, screening for CRC, magnesium, calcium, fibre intake, fat intake, total energy intake, vitamin intake (vitamin B6, folate, vitamin B12, vitamin D, vitamin E), HRT use, use of aspirin or other NSAIDs
Methionine	Zhou 2013[222]	7	431029	6331	5.8 - 22; 13.3	Age, income, waist:hip ratio, BMI, physical activity, smoking status, education, alcohol consumption, family history of CRC, history of CRC, history of colorectal polyps, screening, diabetes, energy intake, calcium, meat, fat intake, fibre intake, iron, vegetables, fruits, vitamin intake (vitamin B6, folate, vitamin D, vitamin E), menopausal status, hormone therapy (OC or HRT), use of aspirin or other NSAIDs
Garlic supplement	Chiavarini 2016[190]	4	304677	2703	3.3 - 24; 9.8	Age, sex, BMI, smoking status, educational level, family history, history of chronic intestinal disease or cholecystectomy, screening, physical activity, fruits, vegetables, total energy intake, alcohol intake, calcium, meat, hormone therapy (HRT), vitamin intake (folate, vitamin C, vitamin D), use of aspirin or other NSAIDs

Abbreviations: BMI, body mass index; CRC, colorectal cancer; HRT, hormone replacement therapy; NSAIDs, non-steroidal anti-inflammatory drugs; OC, oral contraceptive; PUFA, polyunsaturated fatty acid

© 2021 Veettil SK et al. *JAMA Network Open.*

eTable 4. Associations With Nonsignificant Evidence

Exposure	Author; year	Comparison	Summary metric	Credibility assessment									AMSTAR-2
				Random effect size (95% CI)	p-value	I^2	Largest study (95% CI)	Prediction interval (95% CI)	Egger's p-value	Excess significance test		Quality of evidence	
										O/E	p-value		
**Dietary behaviours or diet quality indices**													
Dietary glycaemic index	Reynolds 2019[182]	High vs low	RR	1.10 (0.99-1.22)	0.08955	56.0%	1.06-1.27	0.81-1.47	0.868	3/NA	NA	NS	High
Dietary glycaemic load	Reynolds 2019[182]	High vs low	RR	0.93 (0.85-1.01)	0.08095	32.0%	0.70-0.95	0.76-1.14	0.139	2/NA	NA	NS	High
Eating frequency (3 vs <3 daily meals)	Liu 2014[183]	High vs low	RR	0.85 (0.56-1.31)	0.47531	63.4%	0.78-1.36	NA	NA	0/NA	NA	NS	Moderate
Eating frequency (4 vs <3 daily meals)	Liu 2014[183]	High vs low	RR	0.88 (0.65-1.19)	0.40383	65.7%	0.91-1.32	0.03-24.69	0.069	0/NA	NA	NS	Moderate

© 2021 Veettil SK et al. *JAMA Network Open.*

Eating frequency (≥5 vs <3 daily meals)	Liu 2014 [183]	High vs low	RR	0.75 (0.54-1.05)	0.09413	0.0%	0.51-1.14	NA	NA	0/NA	NA	NS	Moderate
Vegetarian diet	Godos 2016[181]	Yes vs no	RR	0.88 (0.74-1.05)	0.14873	21.3%	0.64-0.98	0.21-3.67	0.919	1/NA	NA	NS	Low
**Food groups or foods**													
Nuts	Schwingshackl 2018[184]	High vs low	RR	0.96 (0.90-1.02)	0.15397	3.7%	0.85-1.02	0.87-1.05	0.478	0/NA	NA	NS	High
Refined grains	Schwingshackl 2018[184]	High vs low	RR	1.46 (0.80-2.67)	0.21776	71.4%	0.85-1.50	NA	NA	1/NA	NA	NS	High
Cruciferous vegetables	Wu 2013[188]	High vs low	RR	0.96 (0.86-1.08)	0.50786	39.6%	0.86-1.09	0.73-1.27	0.398	0/NA	NA	NS	Moderate
Broccoli	Wu 2013[188]	High vs low	RR	0.91 (0.80-1.03)	0.14492	0.0%	0.80-1.08	0.39-2.10	0.920	0/NA	NA	NS	Moderate
Beef	Carr 2016[185]	High vs low	RR	1.10 (0.98-1.23)	0.12048	13.7%	0.86-1.24	0.80-1.51	0.458	1/NA	NA	NS	Low
Poultry	Carr 2016[185]	High vs low	RR	0.97 (0.89-1.07)	0.57776	44.2%	0.86-1.01	0.75-1.26	0.302	2/NA	NA	NS	Low
Fish	Wu 2012[186]	High vs low	OR	0.93 (0.82-1.05)	0.22129	37.2%	0.54-0.88	0.64-1.34	0.817	3/NA	NA	NS	Low
Fruits and vegetables	Aune 2011[187]	High vs low	RR	0.93 (0.86-1.01)	0.08766	28.1%	0.85-1.09	0.77-1.12	0.492	1/NA	NA	NS	Low

© 2021 Veettil SK et al. *JAMA Network Open.*

Soy products	Lu 2017[193]	High vs low	RR	0.86 (0.72-1.03)	0.10126	44.7%	0.78-1.16	0.55-1.36	0.422	2/NA	NA	NS	Low
Cheese	Aune 2012[194]	High vs low	RR	0.94 (0.75-1.18)	0.59378	38.5%	0.56-1.12	0.54-1.65	0.122	1/NA	NA	NS	Low
**Beverages**													
Wine	Xu 2019[203]	Yes vs no	RR	1.01 (0.90-1.13)	0.89148	59.0%	0.93-1.11	0.75-1.35	0.257	2/NA	NA	NS	Moderate
Wine (Light to moderate)	Xu 2019[203]	<2 drinks/d vs non-drinkers	RR	0.94 (0.84-1.05)	0.27068	25.4%	0.83-1.08	0.67-1.32	0.226	0/NA	NA	NS	Moderate
Wine (Heavy)	Xu 2019[203]	≥2 drinks/d vs non-drinkers	RR	1.03 (0.85-1.24)	0.79103	41.8%	1.04-1.40	0.62-1.71	0.439	1/NA	NA	NS	Moderate
Coffee	Gan 2017[199]	High vs low	RR	0.98 (0.90-1.06)	0.63354	41.4%	0.95-1.18	0.77-1.24	0.764	3/NA	NA	NS	Moderate
Fermented milk	Ralston 2014[200]	High vs low	RR	1.01 (0.89-1.15)	0.83901	0.0%	0.86-1.34	0.86-1.19	0.351	0/NA	NA	NS	Moderate
Tea	Chen 2017[196]	High vs low	OR	0.94 (0.86-1.03)	0.17801	32.7%	0.90-1.05	0.74-1.20	0.328	3/NA	NA	NS	Moderate
Green tea	Wang 2012[197]	High vs low	RR	0.93 (0.77-1.12)	0.44432	59.2%	0.97-1.45	0.53-1.62	0.653	2/NA	NA	NS	Critically low
Black tea	Sun 2006[198]	High vs low	OR	1.05 (0.75-1.46)	0.77918	75.1%	0.83-1.22	0.37-2.99	0.704	2/NA	NA	NS	Critically low
**Macronutrients**													

© 2021 Veettil SK et al. *JAMA Network Open.*

Legume fibre	Reynolds 2019[182]	High vs low	RR	0.91 (0.81-1.02)	0.09443	38.0%	0.83-1.04	0.60-1.36	0.214	1/NA	NA	NS	High
Soluble fibre	Reynolds 2019[182]	High vs low	RR	0.84 (0.67-1.05)	0.12380	38.2%	0.69-1.03	0.10-7.29	0.904	1/NA	NA	NS	High
Insoluble fibre	Reynolds 2019[182]	High vs low	RR	0.86 (0.74-1.01)	0.06295	0.0%	0.72-1.06	0.31-2.39	0.893	0/NA	NA	NS	High
Dietary protein	Lai 2017[208]	High vs low	RR	0.94 (0.73-1.21)	0.62644	0.0%	0.64-1.44	0.54-1.63	0.454	0/NA	NA	NS	Moderate
Polyunsaturated fatty acids	Kim 2018[205]	High vs low	RR	0.99 (0.93-1.04)	0.60237	22.0%	0.98-1.03	0.85-1.14	0.965	2/NA	NA	NS	Moderate
Monounsaturated fatty acids	Liu 2011[204]	High vs low	RR	1.04 (0.93-1.16)	0.48534	0.0%	0.87-1.29	0.92-1.18	0.214	1/NA	NA	NS	Low
Saturated fatty acids	Liu 2011[204]	High vs low	RR	1.00 (0.90-1.12)	0.93894	0.0%	0.77-1.14	0.89-1.13	0.037	0/NA	NA	NS	Low
Total dietary fat	Liu 2011[204]	High vs low	RR	0.99 (0.89-1.11)	0.87206	6.9%	0.78-1.17	0.84-1.17	0.092	2/NA	NA	NS	Low
Total n-3 polyunsaturated fatty acids	Chen 2015[206]	High vs low	RR	1.00 (0.93-1.07)	0.96664	8.6%	0.92-1.17	0.89-1.13	0.773	1/NA	NA	NS	Low
Marine n-3 polyunsaturated fatty acids	Chen 2015[206]	High vs low	RR	1.00 (0.93-1.07)	0.96827	0.0%	0.89-1.20	0.92-1.08	0.728	2/NA	NA	NS	Low
Cholesterol	Liu 2011[204]	High vs low	RR	1.14 (0.88-1.47)	0.31702	49.8%	0.70-1.60	0.56-2.30	0.057	1/NA	NA	NS	Low
Carbohydrate	Aune 2012[207]	High vs low	RR	0.93 (0.84-1.04)	0.22270	39.8%	0.75-1.10	0.70-1.25	0.049	3/NA	NA	NS	Low
Sucrose	Aune 2012[207]	High vs low	RR	1.01 (0.87-1.17)	0.93281	63.5%	0.97-1.20	0.65-1.56	0.949	0/NA	NA	NS	Low

© 2021 Veettil SK et al. *JAMA Network Open.*

Fructose	Aune 2012[207]	High vs low	RR	1.06 (0.87-1.28)	0.57654	72.5%	0.90-1.13	0.56-1.99	0.353	2/NA	NA	NS	Low
Fruit fibre	Aune 2011[209]	High vs low	RR	0.94 (0.85-1.04)	0.20801	39.1%	0.95-1.23	0.73-1.20	0.913	2/NA	NA	NS	Low
Vegetable fibre	Aune 2011[209]	High vs low	RR	0.98 (0.91-1.06)	0.63176	0.0%	0.89-1.15	0.90-1.07	0.514	0/NA	NA	NS	Low
**Micronutrients**													
Garlic supplement	Chiavarini 2016[190]	Yes vs no	RR	1.07 (0.91-1.26)	0.41701	27.8%	1.01-1.81	0.74-1.55	0.634	1/NA	NA	NS	Moderate
Flavanols	He 2016[213]	High vs low	OR	1.00 (0.86-1.18)	0.95587	41.3%	0.95-1.21	0.22-4.68	0.924	0/NA	NA	NS	Moderate
Anthocyanins	He 2016[213]	High vs low	OR	0.92 (0.66-1.28)	0.62059	17.0%	0.81-1.91	NA	NA	0/NA	NA	NS	Moderate
Quercetin	Grosso 2017[212]	High vs low	OR	0.98 (0.75-1.29)	0.89412	29.6%	0.75-1.36	0.08-11.45	0.430	0/NA	NA	NS	Low
Kaempferol	Grosso 2017[212]	High vs low	OR	1.12 (0.91-1.38)	0.28931	0.0%	0.85-1.53	0.29-4.32	0.984	0/NA	NA	NS	Low
Myricetin	Grosso 2017[212]	High vs low	OR	1.10 (0.82-1.48)	0.50963	42.4%	0.67-1.18	0.06-19.82	0.541	0/NA	NA	NS	Low
Catechin	Grosso 2017[212]	High vs low	OR	0.89 (0.71-1.11)	0.30474	57.0%	0.77-1.21	0.36-2.17	0.271	1/NA	NA	NS	Low
Phyto-oestrogens	Jiang 2017[214]	High vs low	RR	0.93 (0.83-1.05)	0.23135	0.0%	0.79-1.14	0.78-1.12	0.993	0/NA	NA	NS	Low

Isoflavones	Grosso 2017[212]	High vs low	OR	0.92 (0.82-1.03)	0.15224	0.0%	0.79-1.14	0.78-1.08	0.668	0/NA	NA	NS	Low
Combined carotenoids	Panic 2017[215]	High vs low	RR	1.08 (0.93-1.26)	0.31711	0.0%	0.93-1.28	NA	NA	0/NA	NA	NS	Low
Alpha-carotene	Panic 2017[215]	High vs low	RR	1.05 (0.92-1.21)	0.46477	0.0%	0.88-1.20	0.43-2.59	0.190	0/NA	NA	NS	Low
Beta-carotene	Panic 2017[215]	High vs low	RR	0.98 (0.87-1.11)	0.78542	0.0%	0.78-1.08	0.74-1.30	0.090	0/NA	NA	NS	Low
Lycopene	Panic 2017[215]	High vs low	RR	1.08 (0.94-1.23)	0.26011	0.0%	0.94-1.26	0.45-2.56	0.309	0/NA	NA	NS	Low
Beta-cryptoxanthin	Panic 2017[215]	High vs low	RR	0.99 (0.74-1.34)	0.95857	38.8%	0.78-1.06	NA	NA	0/NA	NA	NS	Low
Lutein and zeaxanthin	Panic 2017[215]	High vs low	RR	1.05 (0.91-1.20)	0.52210	0.0%	0.88-1.20	0.43-2.57	0.585	0/NA	NA	NS	Low
Multivitamin	Liu 2015[217]	High vs low	RR	0.83 (0.65-1.05)	0.12116	68.5%	0.83-1.17	0.42-1.62	0.121	2/NA	NA	NS	Low
Vitamin A	Liu 2015[217]	High vs low	RR	0.89 (0.77-1.03)	0.13230	0.0%	0.70-1.50	0.75-1.06	0.304	1/NA	NA	NS	Low
Vitamin B2	Liu 2015[217]	High vs low	RR	0.89 (0.78-1.00)	0.05723	4.2%	0.66-0.99	0.72-1.08	0.159	1/NA	NA	NS	Low
Vitamin B3	Liu 2015[217]	High vs low	RR	1.18 (0.76-1.84)	0.45683	31.0%	0.70-1.60	NA	NA	0/NA	NA	NS	Low
Vitamin B12	Liu 2015[217]	High vs low	RR	1.10 (0.92-1.32)	0.31145	49.1%	0.72-1.08	0.67-1.80	0.018	0/NA	NA	NS	Low
Vitamin C	Heine-Bröring 2015[216]	Yes vs no	RR	0.92 (0.75-1.11)	0.37799	42.2%	0.73-1.49	0.55-1.53	0.231	1/NA	NA	NS	Low

© 2021 Veettil SK et al. *JAMA Network Open.*

Vitamin C	Liu 2015[217]	High vs low	RR	0.92 (0.80-1.07)	0.30685	40.0%	0.73-1.09	0.62-1.38	0.954	2/NA	NA	NS	Low
Vitamin D	Heine-Bröring 2015[216]	Yes vs no	RR	0.90 (0.81-1.02)	0.09394	46.3%	0.80-1.06	0.67-1.23	0.487	2/NA	NA	NS	Low
Vitamin E	Liu 2015[217]	High vs low	RR	0.88 (0.75-1.04)	0.12177	49.3%	0.85-1.38	0.54-1.43	0.264	1/NA	NA	NS	Low
Flavonoids	Bo 2016[210]	High vs low	OR	1.10 (0.95-1.28)	0.19337	5.0%	0.95-1.50	0.87-1.40	0.825	1/NA	NA	NS	Critically low
Flavonols	Chang 2018[212]	High vs low	RR	1.00 (0.92-1.08)	0.94977	6.6%	0.89-1.14	0.85-1.17	0.777	0/NA	NA	NS	Critically low
Flavones	Chang 2018[212]	High vs low	RR	1.02 (0.94-1.12)	0.62623	0.0%	0.92-1.17	0.58-1.80	0.202	0/NA	NA	NS	Critically low
Flavanones	Chang 2018[212]	High vs low	RR	0.99 (0.91-1.06)	0.71286	0.0%	0.91-1.10	0.83-1.17	0.485	0/NA	NA	NS	Critically low
Flavan-3-ols	Chang 2018[212]	High vs low	RR	1.02 (0.93-1.12)	0.62604	20.4%	0.95-1.21	0.78-1.34	0.908	0/NA	NA	NS	Critically low
Anthocyanidins	Chang 2018[212]	High vs low	RR	1.00 (0.91-1.09)	0.94571	0.0%	0.91-1.13	0.54-1.83	0.125	0/NA	NA	NS	Critically low
Methionine	Zhou 2013[222]	High vs low	RR	0.89 (0.77-1.03)	0.11180	29.1%	0.76-1.28	0.64-1.24	0.632	2/NA	NA	NS	Critically low
NA = not applicable because of non-significant effect estimate/data unavailability; NS= not significant; O/E = observed/expected number of studies with significant results; OR = odds ratio; RR = risk ratio													

© 2021 Veettil SK et al. *JAMA Network Open.*

eTable 5. Sensitivity Analyses for Associations With Class I, II, or III Evidence

Exposure	Author; year	No. of primary studies	No. of study participants	No. of cases	Comparison	Summary metric	Credibility assessment									AMSTAR-2
							Random effect size (95% CI)	p-value	I^2	Largest study (95% CI)	Predictive interval (95% CI)	Egger's p-value	Excess significance test		Quality of evidence	
													O/E	p-value		
**Exclusion of primary studies with number of study participants lower than 25th percentile** (applicable to those meta-analyses with evidence of small-study effects in primary analysis)																
Alcohol (Moderate)	Fedirko 2011[201]	17	2754534	18420	>1-3 drinks/d vs non-/occasional drinkers	RR	1.17 (1.08-1.26)	0.00006	37.3%	1.01-1.13	0.96-1.42	0.014	5/2.5	1.00	Class III	Moderate
Supplemental calcium	Heine-Bröring 2015[216]	6	1029242	9621	Yes vs no	RR	0.89 (0.84-0.95)	0.00051	47.5%	0.88-1.05	0.73-1.09	0.252	2/3.2	NP	Class III	Low
Whole grains	Schwingshackl 2018[184]	7	932818	8943	High vs low	RR	0.87 (0.82-0.94)	0.00011	48.3%	0.88-0.99	0.73-1.04	0.018	4/0.9	0.06	Class III	High

© 2021 Veettil SK et al. *JAMA Network Open.*

**Primary studies adjusted for confounding variables**															
Adherence to Mediterranean diet	Schwingshackl 2017[178]	6	1410030	16102	High vs low	RR	0.86 (0.80-0.92)	8.4 × 10^{-6}	29.7%	0.80-0.99	0.74-1.00	0.841	3/5.0	NP	Class III	Critically low
Adherence to Western diet	Feng 2017[179]	15	1182930	11537	High vs low	OR	1.28 (1.13-1.45)	0.00014	72.2%	1.09-1.44	0.79-2.07	0.173	8/6.5	1.00	Class III	Moderate
Adherence to healthy diet	Feng 2017[179]	15	1182930	11537	High vs low	OR	0.84 (0.76-0.92)	0.00034	56.2%	0.69-0.90	0.60-1.17	0.602	5/7.5	NP	Class III	Moderate
Pesco-vegetarian diet	Godos 2016[181]	3	149516	1506	Yes vs no	RR	0.67 (0.53-0.83)	0.00043	0.0%	0.48-0.94	0.15-2.89	0.437	2/1.7	0.94	Class III	Low
Semi-vegetarian diet	Godos 2016[181]	3	580175	4062	Yes vs no	RR	0.86 (0.79-0.94)	0.00072	0.0%	0.76-0.95	0.72-1.04	0.964	1/1.2	NP	Class III	Low
Red meat	Schwingshackl 2018[184]	21	2154027	21326	High vs low	RR	1.13 (1.08-1.19)	$<10^{-6}$	20.5%	1.15-1.19	1.02-1.26	0.175	3/6.0	NP	Class I	High
Processed meat	Schwingshackl 2018[184]	15	1910983	18646	High vs low	RR	1.14 (1.07-1.23)	0.00013	25.9%	1.09-1.32	0.97-1.35	0.981	4/6.9	NP	Class III	High
Whole grains	Schwingshackl 2018[184]	9	970927	9223	High vs low	RR	0.88 (0.83-0.94)	0.00006	34.9%	0.88-0.99	0.77-1.01	0.067	4/1.0	0.26	Class III	High
Dairy products	Schwingshackl 2018[184]	17	1629366	16910	High vs low	RR	0.83 (0.76-0.89)	$<10^{-6}$	60.3%	0.83-0.95	0.65-1.04	0.170	8/4.0	0.99	Class II	High
Yogurt	Zhang 2019[195]	5	698366	5432	High vs low	OR	0.81 (0.76-0.86)	$<10^{-6}$	0.0%	0.75-0.87	0.72-0.90	0.835	2/1.8	1.00	Class I	Low

© 2021 Veettil SK et al. *JAMA Network Open.*

Alcohol (Moderate)	Fedirko 2011[201]	22	2798092	19123	>1-3 drinks/d vs non-/occasional drinkers	RR	1.24 (1.14-1.34)	$<10^{-6}$	49.3%	1.01-1.13	0.95-1.61	<0.001	9/2.8	0.77	Class II	Moderate
Alcohol (Heavy)	Fedirko 2011[201]	7	738539	5078	≥4 drinks/d vs non-/occasional drinkers	RR	1.58 (1.38-1.80)	$<10^{-6}$	0.0%	1.27-2.16	1.33-1.87	0.802	5/5.8	NP	Class I	Moderate
Non-fermented milk	Ralston 2014[200]	14	892569	5076	High vs low	RR	0.85 (0.78-0.93)	0.00047	0.0%	0.78-1.18	0.77-0.94	0.956	3/0.9	0.96	Class III	Moderate
Total dietary fibre	Reynolds 2019[182]	21	2259486	23004	High vs low	RR	0.84 (0.78-0.89)	$<10^{-6}$	18.1%	0.65-0.85	0.72-0.97	0.529	6/12.1	NP	Class I	High
Dietary calcium	Meng 2019[218]	8	1449526	13640	High vs low	HR	0.77 (0.73-0.82)	$<10^{-6}$	0.0%	0.75-0.94	0.72-0.83	0.598	5/3.9	1.00	Class I	Moderate
Supplemental calcium	Heine-Bröring 2015[216]	7	1064458	9862	Yes vs no	RR	0.88 (0.82-0.94)	0.00009	51.7%	0.88-1.05	0.70-1.09	0.071	3/3.4	NP	Class III	Low
Supplemental calcium	Heine-Bröring 2015[216]	6	929116	8837	High vs low	RR	0.80 (0.72-0.89)	0.00002	30.9%	0.72-1.02	0.63-1.01	0.884	4/2.7	0.95	Class III	Low
**Primary studies with high quality**																
Adherence to Mediterranean diet*	Schwingshackl 2017[178]	6	1410030	16102	High vs low	RR	0.86 (0.80-0.92)	8.4×10^{-6}	29.7%	0.80-0.99	0.74-1.00	0.841	3/5.0	NP	Class III	Critically low

© 2021 Veettil SK et al. *JAMA Network Open.*

Adherence to Western diet	Feng 2017[179]	13	1181915	11449	High vs low	OR	1.23 (1.09-1.40)	0.00010	71.6%	1.09-1.44	0.78-1.95	0.457	6/6.9	NP	Class III	Moderate
Adherence to healthy diet	Feng 2017[179]	13	1181915	11449	High vs low	OR	0.83 (0.75-0.92)	0.00045	59.6%	0.69-0.90	0.59-1.18	0.625	5/6.9	NP	Class III	Moderate
Pesco-vegetarian diet#	Godos 2016[181]	3	149516	1506	Yes vs no	RR	0.67 (0.53-0.83)	0.00043	0.0%	0.48-0.94	0.15-2.89	0.437	2/1.7	0.94	Class III	Low
Semi-vegetarian diet#	Godos 2016[181]	3	580175	4062	Yes vs no	RR	0.86 (0.79-0.94)	0.00072	0.0%	0.76-0.95	0.72-1.04	0.964	1/1.2	NP	Class III	Low
Red meat*	Schwingshackl 2018[184]	21	2154027	21326	High vs low	RR	1.13 (1.08-1.19)	$<10^{-6}$	20.5%	1.15-1.19	1.02-1.26	0.175	3/6.0	NP	Class I	High
Processed meat*	Schwingshackl 2018[184]	15	1910983	18646	High vs low	RR	1.14 (1.07-1.23)	0.00013	25.9%	1.09-1.32	0.97-1.35	0.981	4/6.9	NP	Class III	High
Whole grains*	Schwingshackl 2018[184]	9	970927	9223	High vs low	RR	0.88 (0.83-0.94)	0.00006	34.9%	0.88-0.99	0.77-1.01	0.067	4/1.0	0.26	Class III	High
Dairy products*	Schwingshackl 2018[184]	17	1629366	16910	High vs low	RR	0.83 (0.76-0.89)	$<10^{-6}$	60.3%	0.83-0.95	0.65-1.04	0.170	8/4.0	0.99	Class II	High
Yogurt*	Zhang 2019[195]	5	698366	5432	High vs low	OR	0.81 (0.76-0.86)	$<10^{-6}$	0.0%	0.75-0.87	0.72-0.90	0.835	2/1.8	1.00	Class I	Low
Alcohol (Moderate)	Fedirko 2011[201]	10	1061631	7809	>1-3 drinks/d vs non-/occasional drinkers	RR	1.36 (1.16-1.58)	0.00013	55.3%	0.93-1.29	0.88-2.10	0.016	5/1.4	0.29	Class III	Moderate

© 2021 Veettil SK et al. *JAMA Network Open.*

Alcohol (Heavy)	Fedirko 2011[201]	4	637367	3724	≥4 drinks/d vs non-/occasional drinkers	RR	1.73 (1.47-2.04)	$<10^{-6}$	0.0%	1.27-2.16	1.21-2.49	0.248	4/3.5	0.90	Class I	Moderate
Non-fermented milk	Ralston 2014[200]	10	751312	4184	High vs low	RR	0.83 (0.74-0.94)	0.00343	27.9%	0.78-1.18	0.63-1.10	0.577	3/0.6	0.42	Class III	Moderate
Total dietary fibre	Reynolds 2019[182]	17	2071669	20961	High vs low	RR	0.85 (0.79-0.90)	$<10^{-6}$	10.5%	0.65-0.85	0.76-0.95	0.642	5/10.5	NP	Class I	High
Dietary calcium[#]	Meng 2019[218]	8	1449526	13640	High vs low	HR	0.77 (0.73-0.82)	$<10^{-6}$	0.0%	0.75-0.94	0.72-0.83	0.598	5/3.9	1.00	Class I	Moderate
Supplemental calcium*	Heine-Bröring 2015[216]	7	1064458	9862	Yes vs no	RR	0.88 (0.82-0.94)	0.00009	51.7%	0.88-1.05	0.70-1.09	0.071	3/3.4	NP	Class III	Low
Supplemental calcium*	Heine-Bröring 2015[216]	6	929116	8837	High vs low	RR	0.80 (0.72-0.89)	0.00002	30.9%	0.72-1.02	0.63-1.01	0.884	4/2.7	0.95	Class III	Low

§ Not performed due to limited number of primary studies

*Sensitivity analysis is not possible because no information on quality assessment of primary studies

Sensitivity analysis is not possible because meta-analysis only included good-quality studies

NP = not pertinent, because estimated number is larger than observed, and there is no evidence of excess significance based on assumption made for plausible effect size; O/E = observed/expected number of studies with significant results; OR = odds ratio; RR = risk ratio.

© 2021 Veettil SK et al. *JAMA Network Open.*

eTable 6: Evidence Criteria: Difference Between WCRF and Present Review

**WCRF**[223]	• Evidence from more than one study<br>• Evidence from at least two independent cohort studies<br>• No substantial heterogeneity<br>• Good quality studies to exclude with confidence the possibility that the observed association results from random or systematic error, including confounding, measurement error, and selection bias<br>• Dose-response relationship<br>• Strong and plausible experimental evidence
**Umbrella review**	• Number of cases >1,000<br>• p $<10^{-6}$<br>• I^2 <50%<br>• 95% prediction interval excluding the null<br>• No small-study effects<br>• No excess significance bias

eTable 6.1: Comparison with WCRF meta-analyses for associations with class I evidence in primary analysis

Association	WCRF[223]		Present review	
	Author (Year)	Number of studies reported	Author (Year)	Number of studies reported
Red meat	Alexander (2015) [168]; Chan (2011) [173]	17<br>8	Schwingshackl (2018) [184]	21
Alcohol beverages (heavy intake)	Only dose-response meta-analysis found	-	Fedirko (2011) [201]	7
Total dietary fibre	Aune (2011)	16	Reynolds (2019) [182]	21
Dietary calcium	Only dose-response meta-analysis found	-	Meng (2019) [218]	8
Yogurt	Only dose-response meta-analysis found	-	Zhang (2019) [195]	5

Explanation:

Although WCRF is the latest report, the meta-analyses they used for the intake of red meat and total dietary fibre are different from ours. According to the published methodology by WCRF, the search for articles was updated to April 2015. For our current review paper, a systematic literature search up to September 2019 was performed. Hence, the meta-analyses included for the intake of red meat, total dietary fibre, yogurt, and dietary calcium are from recent papers published between 2018 to 2019, except for heavy alcohol intake which was published in 2011. The meta-analyses included in our review are chosen based on specified selection criteria: meta-analysis with the largest number of primary studies and the one with the largest number of colorectal cancer cases. However, the selection criteria

© 2021 Veettil SK et al. *JAMA Network Open.*

in the WCRF report are unclear. We followed exactly the protocol as suggested by recent umbrella reviews for selection of meta-analysis for evidence grading. We excluded the meta-analyses used by WCRF and they are shown in the exclusion references in this supplementary material. For all of the associations, we used summary estimates for high versus low intake instead of dose-response meta-analysis.

© 2021 Veettil SK et al. *JAMA Network Open.*

eTable 7. Summary Estimates for Concordance in Meta-analyses: Red Meat Intake and Incidence of CRC

Author	Year	RR	95% CI	P-value	Class of evidence
Larsson[a(175)]	2006	1.20	1.11 - 1.31	>10^{-6}	Class III
Chan [(173)]	2011	1.17	1.09 - 1.25	>10^{-6}	Class III
Pham[(141)]	2014	1.18	0.92- 1.53	NS	Not significant (only Japanese population included): excluded in this comparison
Alexander[(168)]	2015	1.16	1.10 - 1.23	NA	Data not available to grade the evidence
Schwingshackl [(184)]	2018	1.14	1.07 - 1.23	>10^{-6}	Class III

Abbreviations: NA: not available; RR, relative risk; 95% CI, 95% confidence interval

*Please note: Dose-response meta-analyses were **not** included in our review.

© 2021 Veettil SK et al. *JAMA Network Open.*

eReferences

1. Godos J, Bella F, Torrisi A, Sciacca S, Galvano F, Grosso G. Dietary patterns and risk of colorectal adenoma: a systematic review and meta-analysis of observational studies. J Hum Nutr Diet [Internet]. 2016;29(6):757–67. Available from: http://ovidsp.ovid.com/ovidweb.cgi?T=JS&PAGE=reference&D=med12&NEWS=N&AN=27 412573

2. Yoon H, Benamouzig R, Little J, Francois-Collange M, Tome D. Systematic review of epidemiological studies on meat, dairy products and egg consumption and risk of colorectal adenomas. Eur J Cancer Prev [Internet]. 2000;9(3):151–64. Available from: http://ovidsp.ovid.com/ovidweb.cgi?T=JS&PAGE=reference&D=med4&NEWS=N&AN=109 54254

3. Wang Y-M, Zhou Q-Y, Zhu J-Z, Zhu K-F, Yu C-H, Li Y-M. Systematic Review with Meta- Analysis: Alcohol Consumption and Risk of Colorectal Serrated Polyp. Dig Dis Sci [Internet]. 2015;60(7):1889–902. Available from: http://ovidsp.ovid.com/ovidweb.cgi?T=JS&PAGE=reference&D=med11&NEWS=N&AN=25 618311

4. Zhu J-Z, Wang Y-M, Zhou Q-Y, Zhu K-F, Yu C-H, Li Y-M. Systematic review with meta- analysis: alcohol consumption and the risk of colorectal adenoma. Aliment Pharmacol Ther [Internet]. 2014;40(4):325–37. Available from: http://ovidsp.ovid.com/ovidweb.cgi?T=JS&PAGE=reference&D=med10&NEWS=N&AN=24 943329

5. Wang J, Wang H, Chen Y, Hao P, Zhang Y. Alcohol ingestion and colorectal neoplasia: a meta-analysis based on a Mendelian randomization approach. Colorectal Dis [Internet]. 2011;13(5):e71-8. Available from: http://ovidsp.ovid.com/ovidweb.cgi?T=JS&PAGE=reference&D=med7&NEWS=N&AN=211 14754

6. Bagnardi V, Blangiardo M, La Vecchia C, Corrao G. A meta-analysis of alcohol drinking and cancer risk. Br J Cancer [Internet]. 2001;85(11):1700–5. Available from: http://ovidsp.ovid.com/ovidweb.cgi?T=JS&PAGE=reference&D=med4&NEWS=N&AN=117 42491

7. Yao Y, Suo T, Andersson R, Cao Y, Wang C, Lu J, et al. Dietary fibre for the prevention of recurrent colorectal adenomas and carcinomas [Internet]. 2017. Available from: http://ovidsp.ovid.com/ovidweb.cgi?T=JS&PAGE=reference&D=coch&NEWS=N&AN=000 75320-100000000-02504

8. Ben Q, Sun Y, Chai R, Qian A, Xu B, Yuan Y. Dietary fiber intake reduces risk for colorectal adenoma: a meta-analysis. Gastroenterology [Internet]. 2014;146(3):689–699.e6. Available from: http://ovidsp.ovid.com/ovidweb.cgi?T=JS&PAGE=reference&D=med10&NEWS=N&AN=24 216326

9. Jacobs ET, Lanza E, Alberts DS, Hsu C-H, Jiang R, Schatzkin A, et al. Fiber, sex, and colorectal adenoma: results of a pooled analysis. Am J Clin Nutr [Internet]. 2006;83(2):343–9. Available from: http://ovidsp.ovid.com/ovidweb.cgi?T=JS&PAGE=reference&D=med5&NEWS=N&AN=164 69993

10. Veettil SK, Ching SM, Lim KG, Saokaew S, Phisalprapa P, Chaiyakunapruk N. Effects of calcium on the incidence of recurrent colorectal adenomas: A systematic review with meta- analysis and trial sequential analysis of randomized controlled trials. Medicine (Baltimore) [Internet]. 2017;96(32):e7661. Available from:

© 2021 Veettil SK et al. *JAMA Network Open.*

http://ovidsp.ovid.com/ovidweb.cgi?T=JS&PAGE=reference&D=med13&NEWS=N&AN=28 796047

11. Keum N, Lee DH, Greenwood DC, Zhang X, Giovannucci EL. Calcium intake and colorectal adenoma risk: dose-response meta-analysis of prospective observational studies. Int J cancer [Internet]. 2015;136(7):1680–7. Available from: http://ovidsp.ovid.com/ovidweb.cgi?T=JS&PAGE=reference&D=medc1&NEWS=N&AN=25 156950

12. Shaukat A, Scouras N, Schunemann HJ. Role of supplemental calcium in the recurrence of colorectal adenomas: a metaanalysis of randomized controlled trials. Am J Gastroenterol [Internet]. 2005;100(2):390–4. Available from: http://ovidsp.ovid.com/ovidweb.cgi?T=JS&PAGE=reference&D=med5&NEWS=N&AN=156 67497

13. Z. Z, Z. Y, Z. H, C. Z. Association between red and processed meat intake and colorectal adenoma incidence and recurrence: A systematic review and meta-analysis. Oncotarget [Internet]. 2018;9(64):32373–82. Available from: http://www.oncotarget.com/index.php?journal=oncotarget&page=article&op=download&path %5B%5D=23561&path%5B%5D=74195

14. Carroll C, Cooper K, Papaioannou D, Hind D, Pilgrim H, Tappenden P. Supplemental calcium in the chemoprevention of colorectal cancer: a systematic review and meta-analysis. Clin Ther [Internet]. 2010;32(5):789–803. Available from: http://ovidsp.ovid.com/ovidweb.cgi?T=JS&PAGE=reference&D=med7&NEWS=N&AN=206 85491

15. Cao H, Wang C, Chai R, Dong Q, Tu S. Iron intake, serum iron indices and risk of colorectal adenomas: a meta-analysis of observational studies. Eur J Cancer Care (Engl) [Internet]. 2017;26(5). Available from: http://ovidsp.ovid.com/ovidweb.cgi?T=JS&PAGE=reference&D=med13&NEWS=N&AN=26 956572

16. Q. D, S. T, H. C, Z. W. Meta-analysis of the association between cruciferous vegetables intake and colorectal adenoma risk. Int J Clin Exp Med [Internet]. 2017;10(6):9288–97. Available from: http://www.ijcem.com/files/ijcem0024790.pdf

17. Bjelakovic G, Nikolova D, Simonetti RG, Gluud C. Antioxidant supplements for preventing gastrointestinal cancers [Internet]. 2008. Available from: http://ovidsp.ovid.com/ovidweb.cgi?T=JS&PAGE=reference&D=coch&NEWS=N&AN=000 75320-100000000-03125

18. Figueiredo JC, Mott LA, Giovannucci E, Wu K, Cole B, Grainge MJ, et al. Folic acid and prevention of colorectal adenomas: a combined analysis of randomized clinical trials. Int J cancer [Internet]. 2011;129(1):192–203. Available from: http://ovidsp.ovid.com/ovidweb.cgi?T=JS&PAGE=reference&D=med7&NEWS=N&AN=211 70989

19. Fife J, Raniga S, Hider PN, Frizelle FA. Folic acid supplementation and colorectal cancer risk: a meta-analysis. Colorectal Dis [Internet]. 2011;13(2):132–7. Available from: http://ovidsp.ovid.com/ovidweb.cgi?T=JS&PAGE=reference&D=med7&NEWS=N&AN=198 63600

20. Kennedy DA, Stern SJ, Moretti M, Matok I, Sarkar M, Nickel C, et al. Folate intake and the risk of colorectal cancer: a systematic review and meta-analysis. Cancer Epidemiol [Internet]. 2011;35(1):2–10. Available from: http://ovidsp.ovid.com/ovidweb.cgi?T=JS&PAGE=reference&D=med7&NEWS=N&AN

=211 77150

21. Ibrahim EM, Zekri JM. Folic acid supplementation for the prevention of recurrence of colorectal adenomas: metaanalysis of interventional trials. Med Oncol [Internet]. 2010;27(3):915–8. Available from: http://ovidsp.ovid.com/ovidweb.cgi?T=JS&PAGE=reference&D=med7&NEWS=N&AN=197 57214

22. S.-X. D, S.-Y. H, W. A, J. G. Folic acid supplementation in prevention of colorectal adenoma recurrence: A meta-analysis. Acad J Second Mil Med Univ [Internet]. 2010;31(9):984–9. Available from: http://ovidsp.ovid.com/ovidweb.cgi?T=JS&PAGE=reference&D=emed11&NEWS=N&AN=3 59717118

23. Xu X, Yu E, Liu L, Zhang W, Wei X, Gao X, et al. Dietary intake of vitamins A, C, and E and the risk of colorectal adenoma: a meta-analysis of observational studies. Eur J Cancer Prev [Internet]. 2013;22(6):529–39. Available from: http://ovidsp.ovid.com/ovidweb.cgi?T=JS&PAGE=reference&D=med9&NEWS=N&AN=240 64545

24. Woo HD, Park S, Oh K, Kim HJ, Shin HR, Moon HK, et al. Diet and cancer risk in the Korean population: a meta- analysis. Asian Pac J Cancer Prev [Internet]. 2014;15(19):8509–19. Available from: http://ovidsp.ovid.com/ovidweb.cgi?T=JS&PAGE=reference&D=medc&NEWS=N&AN=253 39056

25. Wei MY, Garland CF, Gorham ED, Mohr SB, Giovannucci E. Vitamin D and prevention of colorectal adenoma: a meta-analysis. Cancer Epidemiol Biomarkers Prev [Internet]. 2008;17(11):2958–69. Available from: http://ovidsp.ovid.com/ovidweb.cgi?T=JS&PAGE=reference&D=med6&NEWS=N&AN=189 90737

26. Weingarten MA, Zalmanovici A, Yaphe J, Weingarten AM, Zalmanovici Trestioreanu A, Yaphe J, et al. Dietary calcium supplementation for preventing colorectal cancer and adenomatous polyps. Cochrane database Syst Rev [Internet]. 2008;(1):CD003548. Available from: http://ovidsp.ovid.com/ovidweb.cgi?T=JS&PAGE=reference&D=med6&NEWS=N&AN=182 54022

27. Asano T, McLeod RS. Dietary fibre for the prevention of colorectal adenomas and carcinomas. Cochrane database Syst Rev [Internet]. 2002;(2):CD003430. Available from: http://ovidsp.ovid.com/ovidweb.cgi?T=JS&PAGE=reference&D=med4&NEWS=N&AN=120 76480

28. Bailie L, Loughrey MB, Coleman HG. Lifestyle Risk Factors for Serrated Colorectal Polyps: A Systematic Review and Meta-analysis. Gastroenterology [Internet]. 2017;152(1):92–104. Available from: http://ovidsp.ovid.com/ovidweb.cgi?T=JS&PAGE=reference&D=med13&NEWS=N&AN=27 639804

29. Jin H, Leng Q, Li C. Dietary flavonoid for preventing colorectal neoplasms [Internet]. 2012. Available from: http://ovidsp.ovid.com/ovidweb.cgi?T=JS&PAGE=reference&D=coch&NEWS=N&AN=000 75320-100000000-07736

30. Bergsma-Kadijk JA, van 't Veer P, Kampman E, Burema J. Calcium does not protect against colorectal neoplasia. Epidemiology [Internet]. 1996;7(6):590–7. Available from: http://ovidsp.ovid.com/ovidweb.cgi?T=JS&PAGE=reference&D=med4&NEWS=N&AN=889 9384

© 2021 Veettil SK et al. *JAMA Network Open.*

31. Singh S, Singh PP, Murad MH, Singh H, Samadder NJ. Prevalence, risk factors, and outcomes of interval colorectal cancers: a systematic review and meta-analysis. Am J Gastroenterol [Internet]. 2014;109(9):1375–89. Available from: http://ovidsp.ovid.com/ovidweb.cgi?T=JS&PAGE=reference&D=med10&NEWS=N&AN=24 957158

32. Aune D, Chan DSM, Vieira AR, Navarro Rosenblatt DA, Vieira R, Greenwood DC, et al. Red and processed meat intake and risk of colorectal adenomas: a systematic review and meta- analysis of epidemiological studies. Cancer Causes Control [Internet]. 2013;24(4):611–27. Available from: http://ovidsp.ovid.com/ovidweb.cgi?T=JS&PAGE=reference&D=med9&NEWS=N&AN=233 80943

33. Xu B, Sun J, Sun Y, Huang L, Tang Y, Yuan Y. No evidence of decreased risk of colorectal adenomas with white meat, poultry, and fish intake: a meta-analysis of observational studies. Ann Epidemiol [Internet]. 2013;23(4):215–22. Available from: http://ovidsp.ovid.com/ovidweb.cgi?T=JS&PAGE=reference&D=med9&NEWS=N&AN=233 75344

34. Jacobs DRJ, Marquart L, Slavin J, Kushi LH. Whole-grain intake and cancer: an expanded review and meta-analysis. Nutr Cancer [Internet]. 1998;30(2):85–96. Available from: http://ovidsp.ovid.com/ovidweb.cgi?T=JS&PAGE=reference&D=med4&NEWS=N&AN=958 9426

35. Ben Q, Zhong J, Liu J, Wang L, Sun Y, Yv L, et al. Association Between Consumption of Fruits and Vegetables and Risk of Colorectal Adenoma: A PRISMA-Compliant Meta-Analysis of Observational Studies. Medicine (Baltimore) [Internet]. 2015;94(42):e1599. Available from: http://ovidsp.ovid.com/ovidweb.cgi?T=JS&PAGE=reference&D=med11&NEWS=N&AN=26 496264

36. Wang Y, Wang Z, Fu L, Chen Y, Fang J. Legume consumption and colorectal adenoma risk: a meta-analysis of observational studies. PLoS One [Internet]. 2013;8(6):e67335. Available from: http://ovidsp.ovid.com/ovidweb.cgi?T=JS&PAGE=reference&D=med9&NEWS=N&AN=238 26270

37. Ben Q, Wang L, Liu J, Qian A, Wang Q, Yuan Y. Alcohol drinking and the risk of colorectal adenoma: a dose-response meta-analysis. Eur J Cancer Prev [Internet]. 2015;24(4):286–95. Available from: http://ovidsp.ovid.com/ovidweb.cgi?T=JS&PAGE=reference&D=med11&NEWS=N&AN=25 162969

38. Anonymous. Antioxidants don't prevent colorectal cancer. South African Fam Pract [Internet]. 2006;48(8):12. Available from: http://www.tandfonline.com/loi/ojfp20

39. Galeone C, Pelucchi C, La Vecchia C. Added sugar, glycemic index and load in colon cancer risk. Curr Opin Clin Nutr Metab Care [Internet]. 2012;15(4):368–73. Available from: http://ovidsp.ovid.com/ovidweb.cgi?T=JS&PAGE=reference&D=med8&NEWS=N&AN=225 10682

40. Dahm CC, Keogh RH, Spencer EA, Greenwood DC, Key TJ, Fentiman IS, et al. Dietary fiber and colorectal cancer risk: a nested case-control study using food diaries. J Natl Cancer Inst [Internet]. 2010;102(9):614–26. Available from: http://ovidsp.ovid.com/ovidweb.cgi?T=JS&PAGE=reference&D=med7&NEWS=N&AN=204 07088

© 2021 Veettil SK et al. *JAMA Network Open.*

41. Bristow SM, Bolland MJ, MacLennan GS, Avenell A, Grey A, Gamble GD, et al. Calcium supplements and cancer risk: a meta-analysis of randomised controlled trials. Br J Nutr [Internet]. 2013;110(8):1384–93. Available from: http://ovidsp.ovid.com/ovidweb.cgi?T=JS&PAGE=reference&D=med9&NEWS=N&AN=23 6 01861

42. Qin X, Cui Y, Shen L, Sun N, Zhang Y, Li J, et al. Folic acid supplementation and cancer risk: a meta-analysis of randomized controlled trials. Int J cancer [Internet]. 2013;133(5):1033–41. Available from: http://ovidsp.ovid.com/ovidweb.cgi?T=JS&PAGE=reference&D=med9&NEWS=N&AN =233 38728

43. Qin T, Du M, Du H, Shu Y, Wang M, Zhu L. Folic acid supplements and colorectal cancer risk: meta-analysis of randomized controlled trials. Sci Rep [Internet]. 2015;5:12044. Available from: http://ovidsp.ovid.com/ovidweb.cgi?T=JS&PAGE=reference&D=med11&NEWS=N&AN =26 131763

44. Morita M, Yin G, Yoshimitsu S, Ohnaka K, Toyomura K, Kono S, et al. Folate-related nutrients, genetic polymorphisms, and colorectal cancer risk: the fukuoka colorectal cancer study. Asian Pac J Cancer Prev [Internet]. 2013;14(11):6249–56. Available from: http://ovidsp.ovid.com/ovidweb.cgi?T=JS&PAGE=reference&D=medc&NEWS=N&AN =243 77513

45. Bjelakovic G, Gluud LL, Nikolova D, Whitfield K, Krstic G, Wetterslev J, et al. Vitamin D supplementation for prevention of cancer in adults [Internet]. 2014. Available from: http://ovidsp.ovid.com/ovidweb.cgi?T=JS&PAGE=reference&D=coch&NEWS=N&AN =000 75320-100000000-06085

46. Arain MA, Abdul Qadeer A. Systematic review on "vitamin E and prevention of colorectal cancer". Pak J Pharm Sci [Internet]. 2010;23(2):125–30. Available from: http://ovidsp.ovid.com/ovidweb.cgi?T=JS&PAGE=reference&D=med7&NEWS=N&AN =203 63687

47. Cai X, Wang C, Yu W, Fan W, Wang S, Shen N, et al. Selenium Exposure and Cancer Risk: an Updated Meta-analysis and Meta-regression using a comprehensive list of selenium/ selenium supplement/ serum/plasma selenium/ toenail selenium/ anti-oxidant/ minerals And cancer/ breast cancer/ lung cancer/ esophageal. Sci Cit Index, Web Sci [Internet]. 2015 [cited 2019 Nov 11]; Available from: www.nature.com/scientificreports

48. Petimar J, Smith-Warner SA, Fung TT, Rosner B, Chan AT, Hu FB, et al. Recommendation- based dietary indexes and risk of colorectal cancer in the Nurses' Health Study and Health Professionals Follow-up Study. Am J Clin Nutr [Internet]. 2018 Nov 1 [cited 2019 Nov 11];108(5):1092–103. Available from: https://academic.oup.com/ajcn/article/108/5/1092/5115702

49. Ibanez-Sanz G, Diez-Villanueva A, Vilorio-Marques L, Gracia E, Aragones N, Olmedo-Requena R, et al. Possible role of chondroitin sulphate and glucosamine for primary prevention of colorectal cancer. Results from the MCC-Spain study. Sci Rep [Internet]. 2018;8(1):2040. Available from: http://ovidsp.ovid.com/ovidweb.cgi?T=JS&PAGE=reference&D=medc1&NEWS=N&AN =29 391578

50. Vollset SE, Clarke R, Lewington S, Ebbing M, Halsey J, Lonn E, et al. Effects of folic acid supplementation on overall and site-specific cancer incidence during the randomised trials: meta-analyses of data on 50,000 individuals. Armitage J Clarke R, Parish S, Peto R, Collins R, Lonn E, Yusuf S, Manson JE, Glynn R, Grodstein F, Albert CM, Cook NR, Hankey G, Eikelboom JW, Toole J, Malinow MR, Chambless LE, Spence JD, Pettigrew

LC, Howard VJ, Sides EG, Wang CH, Stampfer M, Galan P, BL, editor. Lancet (London, England) [Internet]. 2013;381(9871):1029–36. Available from: http://ovidsp.ovid.com/ovidweb.cgi?T=JS&PAGE=reference&D=med9&NEWS=N&AN=233 52552

51. Shi Y, Yu P-W, Zeng D-Z. Dose-response meta-analysis of poultry intake and colorectal cancer incidence and mortality. Eur J Nutr [Internet]. 2015;54(2):243–50. Available from: http://ovidsp.ovid.com/ovidweb.cgi?T=JS&PAGE=reference&D=med11&NEWS=N&AN=24 788671

52. Tian C, Wang W, Hong Z, Zhang X. Coffee consumption and risk of colorectal cancer: a dose- response analysis of observational studies. Cancer Causes Control [Internet]. 2013;24(6):1265–8. Available from: http://ovidsp.ovid.com/ovidweb.cgi?T=JS&PAGE=reference&D=med9&NEWS=N&AN=235 46611

53. Li G, Ma D, Zhang Y, Zheng W, Wang P. Coffee consumption and risk of colorectal cancer: a meta-analysis of observational studies. Public Health Nutr [Internet]. 2013;16(2):346–57. Available from: http://ovidsp.ovid.com/ovidweb.cgi?T=JS&PAGE=reference&D=med9&NEWS=N&AN=226 94939

54. Galeone C, Turati F, La Vecchia C, Tavani A. Coffee consumption and risk of colorectal cancer: a meta-analysis of case-control studies. Cancer Causes Control [Internet]. 2010;21(11):1949–59. Available from: http://ovidsp.ovid.com/ovidweb.cgi?T=JS&PAGE=reference&D=med7&NEWS=N&AN=206 80435

55. Sun N-H, Huang X-Z, Wang S-B, Li Y, Wang L-Y, Wang H-C, et al. A dose-response meta- analysis reveals an association between vitamin B12 and colorectal cancer risk. Public Health Nutr [Internet]. 2016;19(8):1446–56. Available from: http://ovidsp.ovid.com/ovidweb.cgi?T=JS&PAGE=reference&D=med12&NEWS=N&AN=26 373257

56. Keum N, Aune D, Greenwood DC, Ju W, Giovannucci EL. Calcium intake and colorectal cancer risk: dose-response meta-analysis of prospective observational studies. Int J cancer [Internet]. 2014;135(8):1940–8. Available from: http://ovidsp.ovid.com/ovidweb.cgi?T=JS&PAGE=reference&D=med10&NEWS=N&AN=24 623471

57. Zhao G-H, Jiang S, Yan L-H, Xie L-F, Yang H, Wang Y. Is poultry intake associated with colorectal cancer?. Int J Colorectal Dis [Internet]. 2016;31(5):1087–8. Available from: http://ovidsp.ovid.com/ovidweb.cgi?T=JS&PAGE=reference&D=med12&NEWS=N&AN=26 519150

58. Mizoue T, Tanaka K, Tsuji I, Wakai K, Nagata C, Otani T, et al. Alcohol drinking and colorectal cancer risk: an evaluation based on a systematic review of epidemiologic evidence among the Japanese population. Jpn J Clin Oncol [Internet]. 2006;36(1):25–39. Available from: http://ovidsp.ovid.com/ovidweb.cgi?T=JS&PAGE=reference&D=med5&NEWS=N&AN=168 70695

59. Gorham ED, Garland CF, Garland FC, Grant WB, Mohr SB, Lipkin M, et al. Vitamin D and prevention of colorectal cancer. J Steroid Biochem Mol Biol [Internet]. 2005;97(1–2):179–94. Available from: http://ovidsp.ovid.com/ovidweb.cgi?T=JS&PAGE=reference&D=med5&NEWS=N&AN=162 36494

© 2021 Veettil SK et al. *JAMA Network Open.*

60. Papaioannou D, Cooper KL, Carroll C, Hind D, Squires H, Tappenden P, et al. Antioxidants in the chemoprevention of colorectal cancer and colorectal adenomas in the general population: a systematic review and meta-analysis. Colorectal Dis [Internet]. 2011;13(10):1085–99. Available from: http://ovidsp.ovid.com/ovidweb.cgi?T=JS&PAGE=reference&D=med7&NEWS=N&AN=204 12095

61. Pais R, Dumitrascu DL. Do antioxidants prevent colorectal cancer? A meta-analysis. Rom J Intern Med [Internet]. 2013;51(3–4):152–63. Available from: http://ovidsp.ovid.com/ovidweb.cgi?T=JS&PAGE=reference&D=med9&NEWS=N&AN=246 20628

62. Richardson A, Hayes J, Frampton C, Potter J. Modifiable lifestyle factors that could reduce the incidence of colorectal cancer in New Zealand. N Z Med J [Internet]. 2016;129(1447):13–20. Available from: http://ovidsp.ovid.com/ovidweb.cgi?T=JS&PAGE=reference&D=med12&NEWS=N&AN=27 977648

63. Thies F, Masson LF, Boffetta P, Kris-Etherton P. Oats and bowel disease: a systematic literature review. Br J Nutr [Internet]. 2014;112 Suppl:S31-43. Available from: http://ovidsp.ovid.com/ovidweb.cgi?T=JS&PAGE=reference&D=med10&NEWS=N&AN=25 267242

64. Yip CSC, Lam W, Fielding R. A summary of meat intakes and health burdens. Eur J Clin Nutr [Internet]. 2018;72(1):18–29. Available from: http://ovidsp.ovid.com/ovidweb.cgi?T=JS&PAGE=reference&D=medl&NEWS=N&AN=287 92013

65. T. M. Nutrition in the chemoprevention of gastrointestinal cancer: Where we are in the new millennium. Pract Gastroenterol [Internet]. 2004;28(3):52–64. Available from: http://ovidsp.ovid.com/ovidweb.cgi?T=JS&PAGE=reference&D=emed8&NEWS=N&AN=39 163072

66. Nuri Faruk Aykan. Red meat and colorectal cancer.

67. de Menezes RF, Bergmann A, Thuler LCS. Alcohol consumption and risk of cancer: a systematic literature review. Asian Pac J Cancer Prev [Internet]. 2013;14(9):4965–72. Available from: http://ovidsp.ovid.com/ovidweb.cgi?T=JS&PAGE=reference&D=medc&NEWS=N&AN=241 75760

68. Tavani A, La Vecchia C. Coffee, decaffeinated coffee, tea and cancer of the colon and rectum: a review of epidemiological studies, 1990-2003. Cancer Causes Control [Internet]. 2004;15(8):743–57. Available from: http://ovidsp.ovid.com/ovidweb.cgi?T=JS&PAGE=reference&D=med5&NEWS=N&AN=154 56988

69. Gerber M. Omega-3 fatty acids and cancers: a systematic update review of epidemiological studies. Br J Nutr [Internet]. 2012;107 Suppl:S228-39. Available from: http://ovidsp.ovid.com/ovidweb.cgi?T=JS&PAGE=reference&D=med8&NEWS=N&AN=225 91896

70. Yusof AS, Isa ZM, Shah SA. Dietary patterns and risk of colorectal cancer: a systematic review of cohort studies (2000-2011). Asian Pac J Cancer Prev [Internet]. 2012;13(9):4713–7. Available from: http://ovidsp.ovid.com/ovidweb.cgi?T=JS&PAGE=reference&D=medc&NEWS=N&AN=231 67408

71. Sakai M, Kakutani S, Horikawa C, Tokuda H, Kawashima H, Shibata H, et al.

Arachidonic acid and cancer risk: a systematic review of observational studies. BMC Cancer [Internet]. 2012;12:606. Available from: http://ovidsp.ovid.com/ovidweb.cgi?T=JS&PAGE=reference&D=med8&NEWS=N&AN=232 49186

72. Lampe JW. Dairy products and cancer. J Am Coll Nutr [Internet]. 2011;30(5 Suppl 1):464S– 70S. Available from: http://ovidsp.ovid.com/ovidweb.cgi?T=JS&PAGE=reference&D=med7&NEWS=N&AN=220 81693

73. Grant WB, Garland CF. A critical review of studies on vitamin D in relation to colorectal cancer. Vol. 48, Nutrition and Cancer. Lawrence Erlbaum Associates Inc.; 2004. p. 115–23.

74. Tabung FK. Inaccurate data in meta-analysis "Dietary patterns and colorectal cancer risk: a meta-analysis". Eur J Cancer Prev [Internet]. 2019;28(1):58–9. Available from: http://ovidsp.ovid.com/ovidweb.cgi?T=JS&PAGE=reference&D=medl&NEWS=N&AN=294 24754

75. Asano KT, McLeod RS. Vitamins and minerals for the prevention of colorectal adenomas and carcinomas [Internet]. 2012. Available from: http://ovidsp.ovid.com/ovidweb.cgi?T=JS&PAGE=reference&D=coch&NEWS=N&AN=000 75320-100000000-03322

76. Le NT. Meat Mutagens and Colorectal Adenoma and Cancer: A Problem with a Recently Published Systematic Review and Meta-Analysis. Nutrients [Internet]. 2018;10(3). Available from: http://ovidsp.ovid.com/ovidweb.cgi?T=JS&PAGE=reference&D=medl&NEWS=N&AN=295 09700

77. Giammanco M, Di Majo D, La Guardia M, Aiello S, Crescimannno M, Flandina C, et al. Vitamin D in cancer chemoprevention. Pharm Biol [Internet]. 2015;53(10):1399–434. Available from: http://ovidsp.ovid.com/ovidweb.cgi?T=JS&PAGE=reference&D=med11&NEWS=N&AN=25 856702

78. Farinetti A, Zurlo V, Manenti A, Coppi F, Mattioli AV. Mediterranean diet and colorectal cancer: A systematic review. Nutrition [Internet]. 2017;43–44:83–8. Available from: http://ovidsp.ovid.com/ovidweb.cgi?T=JS&PAGE=reference&D=med13&NEWS=N&AN=28 935150

79. Alicandro G, Tavani A, La Vecchia C. Coffee and cancer risk. Eur J Cancer Prev [Internet]. 2017 Sep [cited 2019 Nov 11];26(5):424–32. Available from: http://insights.ovid.com/crossref?an=00008469-201709000-00010

80. Li D. Omega-3 polyunsaturated fatty acids and non-communicable diseases: meta-analysis based systematic review. Asia Pac J Clin Nutr [Internet]. 2015;24(1):10–5. Available from: http://ovidsp.ovid.com/ovidweb.cgi?T=JS&PAGE=reference&D=med11&NEWS=N&AN=25 740737

81. Boehm K HM. Cochrane Database of Systematic Reviews Green tea (Camellia sinensis) for the prevention of cancer (Review). 2016 [cited 2019 Nov 11]; Available from: www.cochranelibrary.com

82. El Kinany K, Deoula M, Hatime Z, Bennani B, El Rhazi K. Dairy products and colorectal cancer in middle eastern and north African countries: a systematic review. BMC Cancer [Internet]. 2018;18(1):233. Available from:

© 2021 Veettil SK et al. *JAMA Network Open.*

http://ovidsp.ovid.com/ovidweb.cgi?T=JS&PAGE=reference&D=med13&NEWS=N&AN=29 490616

83. Kantor ED, Zhang X, Wu K, Signorello LB, Chan AT, Fuchs CS, et al. Use of glucosamine and chondroitin supplements in relation to risk of colorectal cancer: Results from the Nurses' Health Study and Health Professionals follow-up study. Int J cancer [Internet]. 2016;139(9):1949–57. Available from: http://ovidsp.ovid.com/ovidweb.cgi?T=JS&PAGE=reference&D=med12&NEWS=N&AN=27 357024

84. Vieira AR, Abar L, Chan DSM, Vingeliene S, Polemiti E, Stevens C, et al. Foods and beverages and colorectal cancer risk: a systematic review and meta-analysis of cohort studies, an update of the evidence of the WCRF-AICR Continuous Update Project. Ann Oncol Off J Eur Soc Med Oncol [Internet]. 2017;28(8):1788–802. Available from: http://ovidsp.ovid.com/ovidweb.cgi?T=JS&PAGE=reference&D=med13&NEWS=N&AN=28 407090

85. Choi Y, Giovannucci E, Lee JE. Glycaemic index and glycaemic load in relation to risk of diabetes-related cancers: a meta-analysis. Br J Nutr [Internet]. 2012;108(11):1934–47. Available from: http://ovidsp.ovid.com/ovidweb.cgi?T=JS&PAGE=reference&D=med8&NEWS=N&AN=231 67978

86. Jayedi A, Emadi A, Shab-Bidar S. Dietary Inflammatory Index and Site-Specific Cancer Risk: A Systematic Review and Dose-Response Meta-Analysis. Adv Nutr [Internet]. 2018;9(4):388–403. Available from: http://ovidsp.ovid.com/ovidweb.cgi?T=JS&PAGE=reference&D=medc&NEWS=N&AN=300 32224

87. Y. F, X. J, C. M, Z. G. Meta-analysis of the association between the inflammatory potential of diet and colorectal cancer risk. Oncotarget [Internet]. 2017;8(35):59592–600. Available from: http://www.impactjournals.com/oncotarget/index.php?journal=oncotarget&page=article&op=d ownload&path%5B%5D=19233&path%5B%5D=61578

88. Chiavarini M, Bertarelli G, Minelli L, Fabiani R. Dietary Intake of Meat Cooking-Related Mutagens (HCAs) and Risk of Colorectal Adenoma and Cancer: A Systematic Review and Meta-Analysis. 2017 [cited 2019 Nov 11]; Available from: www.mdpi.com/journal/nutrients

89. Shivappa N, Godos J, Hebert JR, Wirth MD, Piuri G, Speciani AF, et al. Dietary Inflammatory Index and Colorectal Cancer Risk-A Meta-Analysis. Nutrients [Internet]. 2017;9(9). Available from: http://ovidsp.ovid.com/ovidweb.cgi?T=JS&PAGE=reference&D=medc1&NEWS=N&AN=28 930191

90. Sardo Molmenti CL, Steck SE, Thomson CA, Hibler EA, Yang J, Shivappa N, et al. Dietary Inflammatory Index and Risk of Colorectal Adenoma Recurrence: A Pooled Analysis. Nutr Cancer. 2017 Feb 17;69(2):238–47.

91. Fan Y, Jin X, Man C, Gao Z, Wang X. Meta-analysis of the association between the inflammatory potential of diet and colorectal cancer risk [Internet]. Vol. 8. 2017 [cited 2019 Nov 11]. Available from: www.impactjournals.com/oncotarget

92. Berrino F. Life style prevention of cancer recurrence: the yin and the yang. Cancer Treat Res [Internet]. 2014;159:341–51. Available from: http://ovidsp.ovid.com/ovidweb.cgi?T=JS&PAGE=reference&D=med10&NEWS=N&AN

© 2021 Veettil SK et al. *JAMA Network Open.*

=24 114490

93. Badger TM, Ronis MJJ, Simmen RCM, Simmen FA. Soy protein isolate and protection against cancer. J Am Coll Nutr [Internet]. 2005;24(2):146S–149S. Available from: http://ovidsp.ovid.com/ovidweb.cgi?T=JS&PAGE=reference&D=med5&NEWS=N&AN=157 98082

94. Zock PL, Katan MB. Linoleic acid intake and cancer risk: a review and meta-analysis. Am J Clin Nutr [Internet]. 1998;68(1):142–53. Available from: http://ovidsp.ovid.com/ovidweb.cgi?T=JS&PAGE=reference&D=med4&NEWS=N&AN=966 5108

95. Xie L, Mo M, Jia H-X, Liang F, Yuan J, Zhu J. Association between dietary nitrate and nitrite intake and site-specific cancer risk: evidence from observational studies [Internet]. Vol. 7. 2016 [cited 2019 Nov 11]. Available from: www.impactjournals.com/oncotarget

96. Yang B, Wang F-L, Ren X-L, Li D. Biospecimen long-chain N-3 PUFA and risk of colorectal cancer: a meta-analysis of data from 60,627 individuals. PLoS One [Internet]. 2014;9(11):e110574. Available from: http://ovidsp.ovid.com/ovidweb.cgi?T=JS&PAGE=reference&D=med10&NEWS=N&AN=25 375637

97. Kohler LN, Garcia DO, Harris RB, Oren E, Roe DJ, Jacobs ET. Adherence to Diet and Physical Activity Cancer Prevention Guidelines and Cancer Outcomes: A Systematic Review. Cancer Epidemiol Biomarkers Prev [Internet]. 2016;25(7):1018–28. Available from: http://ovidsp.ovid.com/ovidweb.cgi?T=JS&PAGE=reference&D=med12&NEWS=N&AN=27 340121

98. Andersen V, Holst R, Vogel U. Systematic review: diet-gene interactions and the risk of colorectal cancer. Aliment Pharmacol Ther [Internet]. 2013;37(4):383–91. Available from: http://ovidsp.ovid.com/ovidweb.cgi?T=JS&PAGE=reference&D=med9&NEWS=N&AN=232 16531

99. Schwingshackl L, Bogensberger B, Hoffmann G. Diet Quality as Assessed by the Healthy Eating Index, Alternate Healthy Eating Index, Dietary Approaches to Stop Hypertension Score, and Health Outcomes: An Updated Systematic Review and Meta-Analysis of Cohort Studies. J Acad Nutr Diet. 2018 Jan 1;118(1):74–100.e11.

100. Steck SE, Guinter M, Zheng J, Thomson CA. Index-Based Dietary Patterns and Colorectal Cancer Risk: A Systematic Review. Adv Nutr. 2015 Nov 1;6(6):763–73.

101. Zhang C, Wang W, Zhang D. Association between dietary inflammation index and the risk of colorectal cancer: A meta-analysis. Vol. 70, Nutrition and Cancer. Routledge; 2018. p. 14–22.

102. Namazi N, Larijani B, Azadbakht L. Association between the dietary inflammatory index and the incidence of cancer: a systematic review and meta-analysis of prospective studies. Public Health [Internet]. 2018;164:148–56. Available from: http://ovidsp.ovid.com/ovidweb.cgi?T=JS&PAGE=reference&D=medl&NEWS=N&AN=303 21762

103. Castillo-Lancellotti C, Tur Mari JA, Uauy Dagach R. [Folic acid supplementation and colorrectal adenoma recurrence: systematic review]. Supl con acido folico y Prev recurrencia adenomas Color Revis Sist [Internet]. 2012;27(1):13–21. Available from: http://ovidsp.ovid.com/ovidweb.cgi?T=JS&PAGE=reference&D=med8&NEWS=N&AN=225 66300

© 2021 Veettil SK et al. *JAMA Network Open.*

104. Ryan-Harshman Walid Aldoori MB BCh MPA ScD MR. Vol 53: noVember • noVembre 2007 Canadian Family Physician • Le Médecin de famille canadien 1913 Diet and colorectal cancer Review of the evidence. Vol. 53, Can Fam Physician. 2007.

105. Azeem S, Wasif G, Siddiqui AA, Jandrajupalli SB. 16.xx.XXXX Diet and Colorectal Cancer Risk in Asia: A Systematic Review Asian Pac. Asian Pacific J Cancer Prev [Internet]. 2015 [cited 2019 Nov 11];16(xx):XXXX–XXXX. Available from: http://dx.doi.org/10.7314/

106. Aranceta Bartrina J, Pérez Rodrigo C. [Association between sucrose intake and cancer: a review of the evidence]. Nutr Hosp [Internet]. 2013 Jul [cited 2019 Nov 11];28 Suppl 4:95–105. Available from: http://www.ncbi.nlm.nih.gov/pubmed/23834098

107. Schwingshackl L, Hoffmann G. Adherence to Mediterranean diet and risk of cancer: a systematic review and meta-analysis of observational studies. Int J cancer [Internet]. 2014;135(8):1884–97. Available from: http://ovidsp.ovid.com/ovidweb.cgi?T=JS&PAGE=reference&D=med10&NEWS=N&AN=24 599882

108. Schwingshackl L, Hoffmann G. Adherence to Mediterranean diet and risk of cancer: an updated systematic review and meta-analysis of observational studies. Cancer Med [Internet]. 2015;4(12):1933–47. Available from: http://ovidsp.ovid.com/ovidweb.cgi?T=JS&PAGE=reference&D=med11&NEWS=N&AN=26 471010

109. G.H. S. Meat consumption is a risk factor for colorectal cancer: Meta-analysis of case-control studies. Pakistan J Nutr [Internet]. 2006;5(3):230–3. Available from: http://pjbs.org/pjnonline/fin425.pdf

110. Norat T, Lukanova A, Ferrari P, Riboli E. Meat consumption and colorectal cancer risk: dose- response meta-analysis of epidemiological studies. Int J cancer [Internet]. 2002;98(2):241–56. Available from: http://ovidsp.ovid.com/ovidweb.cgi?T=JS&PAGE=reference&D=medc&NEWS=N&AN=118 57415

111. Norat T, Lukanova A, Ferrari P, Riboli E. Meat consumption and colorectal cancer risk: an estimate of attributable and preventable fractions. IARC Sci Publ [Internet]. 2002;156:223–5. Available from: http://ovidsp.ovid.com/ovidweb.cgi?T=JS&PAGE=reference&D=med4&NEWS=N&AN=124 84173

112. Sandhu MS, White IR, McPherson K. Systematic review of the prospective cohort studies on meat consumption and colorectal cancer risk: a meta-analytical approach. Cancer Epidemiol Biomarkers Prev [Internet]. 2001;10(5):439–46. Available from: http://ovidsp.ovid.com/ovidweb.cgi?T=JS&PAGE=reference&D=med4&NEWS=N&AN=113 52852

113. Geelen A, Schouten JM, Kamphuis C, Stam BE, Burema J, Renkema JMS, et al. Fish consumption, n-3 fatty acids, and colorectal cancer: a meta-analysis of prospective cohort studies. Am J Epidemiol [Internet]. 2007;166(10):1116–25. Available from: http://ovidsp.ovid.com/ovidweb.cgi?T=JS&PAGE=reference&D=med5&NEWS=N&AN=178 23383

114. Haas P, Machado MJ, Anton AA, Silva ASS, de Francisco A. Effectiveness of whole grain consumption in the prevention of colorectal cancer: meta-analysis of cohort studies. Int J Food Sci Nutr [Internet]. 2009;60 Suppl 6:1–13. Available from: http://ovidsp.ovid.com/ovidweb.cgi?T=JS&PAGE=reference&D=med6&NEWS=N&AN

=193 06224

115. Koushik A, Hunter DJ, Spiegelman D, Beeson WL, van den Brandt PA, Buring JE, et al. Fruits, vegetables, and colon cancer risk in a pooled analysis of 14 cohort studies. J Natl Cancer Inst [Internet]. 2007;99(19):1471–83. Available from: http://ovidsp.ovid.com/ovidweb.cgi?T=JS&PAGE=reference&D=med5&NEWS=N&AN=178 95473

116. Kashino I, Mizoue T, Tanaka K, Tsuji I, Tamakoshi A, Matsuo K, et al. Vegetable consumption and colorectal cancer risk: an evaluation based on a systematic review and meta- analysis among the Japanese population. Sasazuki S Inoue M, Iwasaki M, Otani T, Sawada N, Shimazu T, Yamaji T, Tsuji I, Tsubono Y, Nishino Y, Tamakoshi A, Matsuo K, Ito H, Wakai K, Nagata C, Mizoue T, Tanaka K TS, editor. Jpn J Clin Oncol [Internet]. 2015;45(10):973–9. Available from: http://ovidsp.ovid.com/ovidweb.cgi?T=JS&PAGE=reference&D=med11&NEWS=N&AN=26 450957

117. Tse G, Eslick GD. Cruciferous vegetables and risk of colorectal neoplasms: a systematic review and meta-analysis. Nutr Cancer [Internet]. 2014;66(1):128–39. Available from: http://ovidsp.ovid.com/ovidweb.cgi?T=JS&PAGE=reference&D=med10&NEWS=N&AN=24 341734

118. Hu J-Y, Hu Y-W, Zhou J-J, Zhang M-W, Li D, Zheng S. Consumption of garlic and risk of colorectal cancer: an updated meta-analysis of prospective studies. World J Gastroenterol [Internet]. 2014;20(41):15413–22. Available from: http://ovidsp.ovid.com/ovidweb.cgi?T=JS&PAGE=reference&D=med10&NEWS=N&AN=25 386091

119. Magalhaes B, Peleteiro B, Lunet N. Dietary patterns and colorectal cancer: systematic review and meta-analysis. Eur J Cancer Prev [Internet]. 2012;21(1):15–23. Available from: http://ovidsp.ovid.com/ovidweb.cgi?T=JS&PAGE=reference&D=med8&NEWS=N&AN=219 46864

120. Fleischauer AT, Poole C, Arab L. Garlic consumption and cancer prevention: meta-analyses of colorectal and stomach cancers. Am J Clin Nutr [Internet]. 2000;72(4):1047–52. Available from: http://ovidsp.ovid.com/ovidweb.cgi?T=JS&PAGE=reference&D=med4&NEWS=N&AN=110 10950

121. Wu L, Wang Z, Zhu J, Murad AL, Prokop LJ, Murad MH. Nut consumption and risk of cancer and type 2 diabetes: a systematic review and meta-analysis. Nutr Rev [Internet]. 2015;73(7):409–25. Available from: http://ovidsp.ovid.com/ovidweb.cgi?T=JS&PAGE=reference&D=med11&NEWS=N&AN=26 081452

122. Yu Y, Jing X, Li H, Zhao X, Wang D. Soy isoflavone consumption and colorectal cancer risk: a systematic review and meta-analysis. Sci Rep [Internet]. 2016;6:25939. Available from: http://ovidsp.ovid.com/ovidweb.cgi?T=JS&PAGE=reference&D=med12&NEWS=N&AN=27 170217

123. Yan L, Spitznagel EL, Bosland MC. Soy consumption and colorectal cancer risk in humans: a meta-analysis. Cancer Epidemiol Biomarkers Prev [Internet]. 2010;19(1):148–58. Available from: http://ovidsp.ovid.com/ovidweb.cgi?T=JS&PAGE=reference&D=med7&NEWS=N&AN=200 56634

124. Huncharek M, Muscat J, Kupelnick B. Colorectal cancer risk and dietary intake of calcium, vitamin D, and dairy products: a meta-analysis of 26,335 cases from 60 observational studies. Nutr Cancer [Internet]. 2009;61(1):47–69. Available from: http://ovidsp.ovid.com/ovidweb.cgi?T=JS&PAGE=reference&D=med6&NEWS=N&AN=191 16875

125. Cho E, Smith-Warner SA, Spiegelman D, Beeson WL, van den Brandt PA, Colditz GA, et al. Dairy foods, calcium, and colorectal cancer: a pooled analysis of 10 cohort studies. J Natl Cancer Inst [Internet]. 2004;96(13):1015–22. Available from: http://ovidsp.ovid.com/ovidweb.cgi?T=JS&PAGE=reference&D=medc2&NEWS=N&AN=15 240785

126. Zhang X, Albanes D, Beeson WL, van den Brandt PA, Buring JE, Flood A, et al. Risk of colon cancer and coffee, tea, and sugar-sweetened soft drink intake: pooled analysis of prospective cohort studies. J Natl Cancer Inst [Internet]. 2010;102(11):771–83. Available from: http://ovidsp.ovid.com/ovidweb.cgi?T=JS&PAGE=reference&D=med7&NEWS=N&AN=204 53203

127. Yu F, Jin Z, Jiang H, Xiang C, Tang J, Li T, et al. Tea consumption and the risk of five major cancers: a dose-response meta-analysis of prospective studies. BMC Cancer [Internet]. 2014;14:197. Available from: http://ovidsp.ovid.com/ovidweb.cgi?T=JS&PAGE=reference&D=med10&NEWS=N&AN=24 636229

128. Sartini M, Bragazzi NL, Spagnolo AM, Schinca E, Ottria G, Dupont C, et al. Coffee Consumption and Risk of Colorectal Cancer: A Systematic Review and Meta-Analysis of Prospective Studies. Nutrients [Internet]. 2019;11(3). Available from: http://ovidsp.ovid.com/ovidweb.cgi?T=JS&PAGE=reference&D=medl&NEWS=N&AN=309 09640

129. Kashino I, Akter S, Mizoue T, Sawada N, Kotemori A, Matsuo K, et al. Coffee drinking and colorectal cancer and its subsites: A pooled analysis of 8 cohort studies in Japan. Int J cancer [Internet]. 2018;143(2):307–16. Available from: http://ovidsp.ovid.com/ovidweb.cgi?T=JS&PAGE=reference&D=medl&NEWS=N&AN=294 46077

130. Gnagnarella P, Gandini S, La Vecchia C, Maisonneuve P. Glycemic index, glycemic load, and cancer risk: a meta-analysis. Am J Clin Nutr [Internet]. 2008;87(6):1793–801. Available from: http://ovidsp.ovid.com/ovidweb.cgi?T=JS&PAGE=reference&D=med6&NEWS=N&AN=185 41570

131. Nakagawa-Senda H, Ito H, Hosono S, Oze I, Tanaka H, Matsuo K. Coffee consumption and the risk of colorectal cancer by anatomical subsite in Japan: Results from the HERPACC studies. Int J cancer [Internet]. 2017;141(2):298–308. Available from: http://ovidsp.ovid.com/ovidweb.cgi?T=JS&PAGE=reference&D=med13&NEWS=N&AN=28 425092

132. Akter S, Kashino I, Mizoue T, Matsuo K, Ito H, Wakai K, et al. Coffee drinking and colorectal cancer risk: an evaluation based on a systematic review and meta-analysis among the Japanese population. Jpn J Clin Oncol [Internet]. 2016;46(8):781–7. Available from: http://ovidsp.ovid.com/ovidweb.cgi?T=JS&PAGE=reference&D=med12&NEWS=N&AN=27 174958

133. Je Y, Liu W, Giovannucci E. Coffee consumption and risk of colorectal cancer: a systematic review and meta-analysis of prospective cohort studies. Int J cancer [Internet].

2009;124(7):1662–8. Available from:
http://ovidsp.ovid.com/ovidweb.cgi?T=JS&PAGE=reference&D=med6&NEWS=N&AN=191 15212

134. Choi Y-J, Myung S-K, Lee J-H. Light Alcohol Drinking and Risk of Cancer: A Meta-Analysis of Cohort Studies. Cancer Res Treat [Internet]. 2018;50(2):474–87. Available from:
http://ovidsp.ovid.com/ovidweb.cgi?T=JS&PAGE=reference&D=med13&NEWS=N&AN=28 546524

135. Jayasekara H, MacInnis RJ, Room R, English DR. Long-Term Alcohol Consumption and Breast, Upper Aero-Digestive Tract and Colorectal Cancer Risk: A Systematic Review and Meta-Analysis. Alcohol Alcohol [Internet]. 2016;51(3):315–30. Available from:
http://ovidsp.ovid.com/ovidweb.cgi?T=JS&PAGE=reference&D=med12&NEWS=N&AN=26 400678

136. Y. W, H. D, H. Y. A pooled analysis of alcohol intake and colorectal cancer. Int J Clin Exp Med [Internet]. 2015;8(5):6878–89. Available from:
http://www.ijcem.com/files/ijcem0006962.pdf

137. Cai S, Li Y, Ding Y, Chen K, Jin M. Alcohol drinking and the risk of colorectal cancer death: a meta-analysis. Eur J Cancer Prev [Internet]. 2014;23(6):532–9. Available from:
http://ovidsp.ovid.com/ovidweb.cgi?T=JS&PAGE=reference&D=med10&NEWS=N&AN=25 170915

138. Mizoue T, Inoue M, Wakai K, Nagata C, Shimazu T, Tsuji I, et al. Alcohol drinking and colorectal cancer in Japanese: a pooled analysis of results from five cohort studies. Tsugane S Sasazuki S, Iwasaki M, Otani T, Kurahashi N, Shimazu T, Tsuji I, Tsubono Y, Nishino Y, Wakai K, Matsuo K, Nagata C, Mizoue T, Tanaka K IM, editor. Am J Epidemiol [Internet]. 2008;167(12):1397–406. Available from:
http://ovidsp.ovid.com/ovidweb.cgi?T=JS&PAGE=reference&D=med6&NEWS=N&AN=184 20544

139. Moskal A, Norat T, Ferrari P, Riboli E. Alcohol intake and colorectal cancer risk: a dose- response meta-analysis of published cohort studies. Int J cancer [Internet]. 2007;120(3):664–
71. Available from:
http://ovidsp.ovid.com/ovidweb.cgi?T=JS&PAGE=reference&D=med5&NEWS=N&AN=17 0 96321

140. Cho E, Smith-Warner SA, Ritz J, van den Brandt PA, Colditz GA, Folsom AR, et al. Alcohol intake and colorectal cancer: a pooled analysis of 8 cohort studies. Ann Intern Med [Internet]. 2004;140(8):603–13. Available from:
http://ovidsp.ovid.com/ovidweb.cgi?T=JS&PAGE=reference&D=medc&NEWS=N&AN=150 96331

141. Pham NM, Mizoue T, Tanaka K, Tsuji I, Tamakoshi A, Matsuo K, et al. Meat consumption and colorectal cancer risk: an evaluation based on a systematic review of epidemiologic evidence among the Japanese population. Sasazuki S Inoue M, Iwasaki M, Otani T, Sawada N, Shimazu T, Yamaji T, Tsuji I, Tsubono Y, Nishino Y, Tamakoshi A, Matsuo K, Ito H, Wakai K, Nagata C, Mizoue T, Tanaka K TS, editor. Jpn J Clin Oncol [Internet]. 2014;44(7):641–50. Available from:
http://ovidsp.ovid.com/ovidweb.cgi?T=JS&PAGE=reference&D=med10&NEWS=N&AN=24 842864

142. Longnecker MP, Orza MJ, Adams ME, Vioque J, Chalmers TC. A meta-analysis of alcoholic beverage consumption in relation to risk of colorectal cancer. Cancer Causes Control [Internet]. 1990;1(1):59–68. Available from:

http://ovidsp.ovid.com/ovidweb.cgi?T=JS&PAGE=reference&D=med3&NEWS=N&AN=215 1680

143. Alexander DD, Cushing CA, Lowe KA, Sceurman B, Roberts MA. Meta-analysis of animal fat or animal protein intake and colorectal cancer. Am J Clin Nutr [Internet]. 2009;89(5):1402–9. Available from: http://ovidsp.ovid.com/ovidweb.cgi?T=JS&PAGE=reference&D=med6&NEWS=N&AN=19 2 61724

144. Shen X-J, Zhou J-D, Dong J-Y, Ding W-Q, Wu J-C. Dietary intake of n-3 fatty acids and colorectal cancer risk: a meta-analysis of data from 489 000 individuals. Br J Nutr [Internet]. 2012;108(9):1550–6. Available from: http://ovidsp.ovid.com/ovidweb.cgi?T=JS&PAGE=reference&D=med8&NEWS=N&AN=229 06228

145. Huang J, Pan G, Jiang H, Li W, Dong J, Zhang H, et al. A meta-analysis between dietary carbohydrate intake and colorectal cancer risk: evidence from 17 observational studies. Biosci Rep [Internet]. 2017;37(2). Available from: http://ovidsp.ovid.com/ovidweb.cgi?T=JS&PAGE=reference&D=med13&NEWS=N&AN=28 298476

146. M. M. Dietary fibre protective against colorectal cancer patients in Asia: A meta-analysis. Open Access Maced J Med Sci [Internet]. 2019;7(10):1723–7. Available from: http://www.id- press.eu/mjms/article/download/2827/3427

147. Ma Y, Hu M, Zhou L, Ling S, Li Y, Kong B, et al. Dietary fiber intake and risks of proximal and distal colon cancers: A meta-analysis. Medicine (Baltimore) [Internet]. 2018;97(36):e11678. Available from: http://ovidsp.ovid.com/ovidweb.cgi?T=JS&PAGE=reference&D=med13&NEWS=N&AN=30 200062

148. Gianfredi V, Salvatori T, Villarini M, Moretti M, Nucci D, Realdon S. Is dietary fibre truly protective against colon cancer? A systematic review and meta-analysis. Int J Food Sci Nutr [Internet]. 2018;69(8):904–15. Available from: http://ovidsp.ovid.com/ovidweb.cgi?T=JS&PAGE=reference&D=medc1&NEWS=N&AN=29 516760

149. Park Y, Hunter DJ, Spiegelman D, Bergkvist L, Berrino F, van den Brandt PA, et al. Dietary fiber intake and risk of colorectal cancer: a pooled analysis of prospective cohort studies. JAMA [Internet]. 2005;294(22):2849–57. Available from: http://ovidsp.ovid.com/ovidweb.cgi?T=JS&PAGE=reference&D=med5&NEWS=N&AN=163 52792

150. Woo HD, Kim J. Dietary flavonoid intake and risk of stomach and colorectal cancer. World J Gastroenterol [Internet]. 2013;19(7):1011–9. Available from: http://ovidsp.ovid.com/ovidweb.cgi?T=JS&PAGE=reference&D=med9&NEWS=N&AN=234 67443

151. L. L, Y. Y, H. H, E. C, L. D, J. D. Flavan-3-ols consumption and cancer risk: A meta-analysis of epidemiologic studies. Oncotarget [Internet]. 2016;7(45):73573–92. Available from: http://www.impactjournals.com/oncotarget/index.php?journal=oncotarget&page=article&op=d ownload&path%5B%5D=12017&path%5B%5D=39641

152. Johnson CM, Wei C, Ensor JE, Smolenski DJ, Amos CI, Levin B, et al. Meta-analyses of colorectal cancer risk factors. Cancer Causes Control [Internet]. 2013;24(6):1207–22. Available from:

http://ovidsp.ovid.com/ovidweb.cgi?T=JS&PAGE=reference&D=med9&NEWS=N&AN=235 63998

153. Keum N, Kim H, Giovannucci EL. Calcium as a chemopreventive agent against colorectal neoplasm: does obesity play a role?. Cancer Causes Control [Internet]. 2017;28(8):853–6. Available from: http://ovidsp.ovid.com/ovidweb.cgi?T=JS&PAGE=reference&D=med13&NEWS=N&AN=28 677025

154. Bastide NM, Pierre FHF, Corpet DE. Heme iron from meat and risk of colorectal cancer: a meta-analysis and a review of the mechanisms involved. Cancer Prev Res (Phila) [Internet]. 2011;4(2):177–84. Available from: http://ovidsp.ovid.com/ovidweb.cgi?T=JS&PAGE=reference&D=med7&NEWS=N&AN=212 09396

155. Ko HJ, Youn CH, Kim HM, Cho YJ, Lee GH, Lee WK. Dietary magnesium intake and risk of cancer: a meta-analysis of epidemiologic studies. Nutr Cancer [Internet]. 2014;66(6):915–23. Available from: http://ovidsp.ovid.com/ovidweb.cgi?T=JS&PAGE=reference&D=med10&NEWS=N&AN=24 910891

156. Qu X, Jin F, Hao Y, Zhu Z, Li H, Tang T, et al. Nonlinear association between magnesium intake and the risk of colorectal cancer. Eur J Gastroenterol Hepatol [Internet]. 2013;25(3):309–18. Available from: http://ovidsp.ovid.com/ovidweb.cgi?T=JS&PAGE=reference&D=med9&NEWS=N&AN=232 22473

157. Wark PA, Lau R, Norat T, Kampman E. Magnesium intake and colorectal tumor risk: a case- control study and meta-analysis. Am J Clin Nutr [Internet]. 2012;96(3):622–31. Available from: http://ovidsp.ovid.com/ovidweb.cgi?T=JS&PAGE=reference&D=med8&NEWS=N&AN=228 54408

158. Mannisto S, Yaun S-S, Hunter DJ, Spiegelman D, Adami H-O, Albanes D, et al. Dietary carotenoids and risk of colorectal cancer in a pooled analysis of 11 cohort studies. Am J Epidemiol [Internet]. 2007;165(3):246–55. Available from: http://ovidsp.ovid.com/ovidweb.cgi?T=JS&PAGE=reference&D=med5&NEWS=N&AN=171 58857

159. Druesne-Pecollo N, Latino-Martel P, Norat T, Barrandon E, Bertrais S, Galan P, et al. Beta- carotene supplementation and cancer risk: a systematic review and metaanalysis of randomized controlled trials. Int J cancer [Internet]. 2010;127(1):172–84. Available from: http://ovidsp.ovid.com/ovidweb.cgi?T=JS&PAGE=reference&D=med7&NEWS=N&AN=198 76916

160. Sanjoaquin MA, Allen N, Couto E, Roddam AW, Key TJ. Folate intake and colorectal cancer risk: a meta-analytical approach. Int J cancer [Internet]. 2005;113(5):825–8. Available from: http://ovidsp.ovid.com/ovidweb.cgi?T=JS&PAGE=reference&D=med5&NEWS=N&AN=154 99620

161. Burr NE, Hull MA, Subramanian V. Folic Acid Supplementation May Reduce Colorectal Cancer Risk in Patients With Inflammatory Bowel Disease: A Systematic Review and Meta- Analysis. J Clin Gastroenterol [Internet]. 2017;51(3):247–53. Available from: http://ovidsp.ovid.com/ovidweb.cgi?T=JS&PAGE=reference&D=med13&NEWS=N&AN=26 905603

162. Larsson SC, Orsini N, Wolk A. Vitamin B6 and risk of colorectal cancer: a meta-analysis

© 2021 Veettil SK et al. *JAMA Network Open.*

of prospective studies. JAMA [Internet]. 2010;303(11):1077–83. Available from: http://ovidsp.ovid.com/ovidweb.cgi?T=JS&PAGE=reference&D=med7&NEWS=N&AN=202 33826

163. Jia K, Wang R, Tian J. Vitamin B6 Intake and the Risk of Colorectal Cancer: A Meta-Analysis of Prospective Cohort Studies. Nutr Cancer [Internet]. 2017;69(5):723–31. Available from: http://ovidsp.ovid.com/ovidweb.cgi?T=JS&PAGE=reference&D=med13&NEWS=N&AN=28 569561

164. Alexander DD, Miller AJ, Cushing CA, Lowe KA. Processed meat and colorectal cancer: a quantitative review of prospective epidemiologic studies. Eur J Cancer Prev [Internet]. 2010;19(5):328–41. Available from: http://ovidsp.ovid.com/ovidweb.cgi?T=JS&PAGE=reference&D=med7&NEWS=N&AN=204 95462

165. Park Y, Spiegelman D, Hunter DJ, Albanes D, Bergkvist L, Buring JE, et al. Intakes of vitamins A, C, and E and use of multiple vitamin supplements and risk of colon cancer: a pooled analysis of prospective cohort studies. Cancer Causes Control [Internet]. 2010;21(11):1745–57. Available from: http://ovidsp.ovid.com/ovidweb.cgi?T=JS&PAGE=reference&D=med7&NEWS=N&AN=208 20901

166. Li Y, Yang H, Cao J. Association between alcohol consumption and cancers in the Chinese population--a systematic review and meta-analysis. PLoS One [Internet]. 2011;6(4):e18776. Available from: http://ovidsp.ovid.com/ovidweb.cgi?T=JS&PAGE=reference&D=med7&NEWS=N&AN=215 26212

167. Ma Y, Zhang P, Wang F, Yang J, Liu Z, Qin H. Association between vitamin D and risk of colorectal cancer: a systematic review of prospective studies. J Clin Oncol [Internet]. 2011;29(28):3775–82. Available from: http://ovidsp.ovid.com/ovidweb.cgi?T=JS&PAGE=reference&D=med7&NEWS=N&AN=218 76081

168. Alexander DD, Weed DL, Miller PE, Mohamed MA. Red Meat and Colorectal Cancer: A Quantitative Update on the State of the Epidemiologic Science. J Am Coll Nutr [Internet]. 2015;34(6):521–43. Available from: http://ovidsp.ovid.com/ovidweb.cgi?T=JS&PAGE=reference&D=med11&NEWS=N&AN=25 941850

169. Wang X, Yang HH, Liu Y, Zhou Q, Chen ZH. Lycopene Consumption and Risk of Colorectal Cancer: A Meta-Analysis of Observational Studies. Nutr Cancer. 2016 Oct 2;68(7):1083–96.

170. Kim D-H, Smith-Warner SA, Spiegelman D, Yaun S-S, Colditz GA, Freudenheim JL, et al. Cancer Causes Control. 2010;21(11):1919–30.

171. Tse G, Eslick GD. Soy and isoflavone consumption and risk of gastrointestinal cancer: a systematic review and meta-analysis. Eur J Nutr [Internet]. 2016;55(1):63–73. Available from: http://ovidsp.ovid.com/ovidweb.cgi?T=JS&PAGE=reference&D=med12&NEWS=N&AN=25 547973

172. Barrubés L, Babio N, Becerra-Tomás N, Rosique-Esteban N, Salas-Salvadó J. Association Between Dairy Product Consumption and Colorectal Cancer Risk in Adults: A Systematic Review and Meta-Analysis of Epidemiologic Studies. Adv Nutr [Internet]. 2019 May 1 [cited 2019 Nov 12];10(suppl_2):S190–211. Available from:

https://academic.oup.com/advances/article/10/suppl_2/S190/5489434

173. Chan DSM, Lau R, Aune D, Vieira R, Greenwood DC, Kampman E, et al. Red and processed meat and colorectal cancer incidence: meta-analysis of prospective studies. PLoS One [Internet]. 2011;6(6):e20456. Available from: http://ovidsp.ovid.com/ovidweb.cgi?T=JS&PAGE=reference&D=med7&NEWS=N&AN=216 74008

174. K. S. Risk of colorectal cancer in relation to frequency and total amount of red meat consumption. Systematic review and meta-analysis. Arch Med Sci [Internet]. 2010;6(4):605–10. Available from: http://www.termedia.pl/Journal/-19/pdf-15186- 10?filename=Riskofcolorectal.pdf

175. Larsson SC, Wolk A. Meat consumption and risk of colorectal cancer: a meta-analysis of prospective studies. Int J cancer [Internet]. 2006;119(11):2657–64. Available from: http://ovidsp.ovid.com/ovidweb.cgi?T=JS&PAGE=reference&D=med5&NEWS=N&AN=169 91129

176. Touvier M, Chan DSM, Lau R, Aune D, Vieira R, Greenwood DC, et al. Meta-analyses of vitamin D intake, 25-hydroxyvitamin D status, vitamin D receptor polymorphisms, and colorectal cancer risk. Cancer Epidemiol Biomarkers Prev [Internet]. 2011;20(5):1003–16. Available from: http://ovidsp.ovid.com/ovidweb.cgi?T=JS&PAGE=reference&D=med7&NEWS=N&AN=213 78269

177. Yu XF, Wang YQ, Zou J, Dong J. A meta-analysis of the effects of energy intake on risk of digestive cancers. World J Gastroenterol. 2012;18(48):7362–70.

178. Schwingshackl L, Schwedhelm C, Galbete C, Hoffmann G. Adherence to Mediterranean Diet and Risk of Cancer: An Updated Systematic Review and Meta-Analysis. Nutrients [Internet]. 2017;9(10). Available from: http://ovidsp.ovid.com/ovidweb.cgi?T=JS&PAGE=reference&D=med13&NEWS=N&AN=28 954418

179. Feng Y-L, Shu L, Zheng P-F, Zhang X-Y, Si C-J, Yu X-L, et al. Dietary patterns and colorectal cancer risk: a meta-analysis. Eur J Cancer Prev [Internet]. 2017;26(3):201–11. Available from: http://ovidsp.ovid.com/ovidweb.cgi?T=JS&PAGE=reference&D=medc1&NEWS=N&AN=26 945285

180. Grosso G, Bella F, Godos J, Sciacca S, Del Rio D, Ray S, et al. Possible role of diet in cancer: systematic review and multiple meta-analyses of dietary patterns, lifestyle factors, and cancer risk. Nutr Rev [Internet]. 2017;75(6):405–19. Available from: http://ovidsp.ovid.com/ovidweb.cgi?T=JS&PAGE=reference&D=med13&NEWS=N&AN=28 969358

181. Godos J, Bella F, Sciacca S, Galvano F, Grosso G. Vegetarianism and breast, colorectal and prostate cancer risk: an overview and meta-analysis of cohort studies. J Hum Nutr Diet [Internet]. 2017;30(3):349–59. Available from: http://ovidsp.ovid.com/ovidweb.cgi?T=JS&PAGE=reference&D=med13&NEWS=N&AN=27 709695

182. Reynolds A, Mann J, Cummings J, Winter N, Mete E, Te Morenga L. Carbohydrate quality and human health: a series of systematic reviews and meta-analyses. Lancet (London, England) [Internet]. 2019;393(10170):434–45. Available from: http://ovidsp.ovid.com/ovidweb.cgi?T=JS&PAGE=reference&D=medc&NEWS=N&AN=306 38909

183. Liu Y, Tang W, Zhai L, Yang S, Wu J, Xie L, et al. Meta-analysis: eating frequency and risk of colorectal cancer. Tumour Biol [Internet]. 2014;35(4):3617–25. Available from: http://ovidsp.ovid.com/ovidweb.cgi?T=JS&PAGE=reference&D=med10&NEWS=N&AN=24 307626

184. Schwingshackl L, Schwedhelm C, Hoffmann G, Knuppel S, Laure Preterre A, Iqbal K, et al. Food groups and risk of colorectal cancer. Int J cancer [Internet]. 2018;142(9):1748–58. Available from: http://ovidsp.ovid.com/ovidweb.cgi?T=JS&PAGE=reference&D=medl&NEWS=N&AN=292 10053

185. Carr PR, Walter V, Brenner H, Hoffmeister M. Meat subtypes and their association with colorectal cancer: Systematic review and meta-analysis. Vol. 138, International Journal of Cancer. Wiley-Liss Inc.; 2016. p. 293–302.

186. Wu S, Feng B, Li K, Zhu X, Liang S, Liu X, et al. Fish consumption and colorectal cancer risk in humans: a systematic review and meta-analysis. Am J Med [Internet]. 2012;125(6):551– 9.e5. Available from: http://ovidsp.ovid.com/ovidweb.cgi?T=JS&PAGE=reference&D=med8&NEWS=N&AN=225 13196

187. Aune D, Lau R, Chan DSM, Vieira R, Greenwood DC, Kampman E, et al. Nonlinear reduction in risk for colorectal cancer by fruit and vegetable intake based on meta-analysis of prospective studies. Gastroenterology [Internet]. 2011;141(1):106–18. Available from: http://ovidsp.ovid.com/ovidweb.cgi?T=JS&PAGE=reference&D=med7&NEWS=N&AN=216 00207

188. Wu QJ, Yang Y, Vogtmann E, Wang J, Han LH, Li HL, et al. Cruciferous vegetables intake and the risk of colorectal cancer: a meta-analysis of observational studies. Ann Oncol Off J Eur Soc Med Oncol [Internet]. 2013;24(4):1079–87. Available from: http://ovidsp.ovid.com/ovidweb.cgi?T=JS&PAGE=reference&D=med9&NEWS=N&AN=232 11939

189. Zhu B, Zou L, Qi L, Zhong R, Miao X. Allium vegetables and garlic supplements do not reduce risk of colorectal cancer, based on meta-analysis of prospective studies. Clin Gastroenterol Hepatol [Internet]. 2014;12(12):1991-e121. Available from: http://ovidsp.ovid.com/ovidweb.cgi?T=JS&PAGE=reference&D=med10&NEWS=N&AN=24 681077

190. Chiavarini M, Minelli L, Fabiani R. Garlic consumption and colorectal cancer risk in man: a systematic review and meta-analysis. Public Health Nutr [Internet]. 2016;19(2):308–17. Available from: http://ovidsp.ovid.com/ovidweb.cgi?T=JS&PAGE=reference&D=med12&NEWS=N&AN=25 945653

191. Turati F, Guercio V, Pelucchi C, La Vecchia C, Galeone C. Colorectal cancer and adenomatous polyps in relation to allium vegetables intake: a meta-analysis of observational studies. Mol Nutr Food Res [Internet]. 2014;58(9):1907–14. Available from: http://ovidsp.ovid.com/ovidweb.cgi?T=JS&PAGE=reference&D=med10&NEWS=N&AN=24 976533

192. Zhu B, Sun Y, Qi L, Zhong R, Miao X. Dietary legume consumption reduces risk of colorectal cancer: evidence from a meta-analysis of cohort studies. Sci Rep [Internet]. 2015;5:8797. Available from: http://ovidsp.ovid.com/ovidweb.cgi?T=JS&PAGE=reference&D=med11&NEWS=N&AN=25 739376

© 2021 Veettil SK et al. *JAMA Network Open.*

193. Lu D, Pan C, Ye C, Duan H, Xu F, Yin L, et al. Meta-analysis of Soy Consumption and Gastrointestinal Cancer Risk. Sci Rep [Internet]. 2017;7(1):4048. Available from: http://ovidsp.ovid.com/ovidweb.cgi?T=JS&PAGE=reference&D=medl&NEWS=N&AN=286 42459

194. Aune D, Lau R, Chan DSM, Vieira R, Greenwood DC, Kampman E, et al. Dairy products and colorectal cancer risk: a systematic review and meta-analysis of cohort studies. Ann Oncol Off J Eur Soc Med Oncol [Internet]. 2012;23(1):37–45. Available from: http://ovidsp.ovid.com/ovidweb.cgi?T=JS&PAGE=reference&D=med8&NEWS=N&AN=21 6 17020

195. Zhang K, Dai H, Liang W, Zhang L, Deng Z. Fermented dairy foods intake and risk of cancer. Int J cancer [Internet]. 2019;144(9):2099–108. Available from: http://ovidsp.ovid.com/ovidweb.cgi?T=JS&PAGE=reference&D=medl&NEWS=N&AN=303 74967

196. Chen Y, Wu Y, Du M, Chu H, Zhu L, Tong N, et al. An inverse association between tea consumption and colorectal cancer risk. Oncotarget [Internet]. 2017;8(23):37367–76. Available from: http://www.impactjournals.com/oncotarget/index.php?journal=oncotarget&page=article&op=d ownload&path%5B%5D=16959&path%5B%5D=54265

197. Wang Z-H, Gao Q-Y, Fang J-Y. Green tea and incidence of colorectal cancer: evidence from prospective cohort studies. Nutr Cancer [Internet]. 2012;64(8):1143–52. Available from: http://ovidsp.ovid.com/ovidweb.cgi?T=JS&PAGE=reference&D=med8&NEWS=N&AN=231 63842

198. Sun C-L, Yuan J-M, Koh W-P, Yu MC. Green tea, black tea and colorectal cancer risk: a meta-analysis of epidemiologic studies. Carcinogenesis [Internet]. 2006;27(7):1301–9. Available from: http://ovidsp.ovid.com/ovidweb.cgi?T=JS&PAGE=reference&D=med5&NEWS=N&AN=166 38787

199. Y. G, J. W, S. Z, L. L, S. C, N. M, et al. Association of coffee consumption with risk of colorectal cancer: A meta-analysis of prospective cohort studies. Oncotarget [Internet]. 2017;8(12):18699–711. Available from: http://www.impactjournals.com/oncotarget/index.php?journal=oncotarget&page=article&op=d ownload&path%5B%5D=8627&path%5B%5D=25797

200. Ralston RA, Truby H, Palermo CE, Walker KZ. Colorectal cancer and nonfermented milk, solid cheese, and fermented milk consumption: a systematic review and meta-analysis of prospective studies. Crit Rev Food Sci Nutr [Internet]. 2014;54(9):1167–79. Available from: http://ovidsp.ovid.com/ovidweb.cgi?T=JS&PAGE=reference&D=medc1&NEWS=N&AN=24 499149

201. Fedirko V, Tramacere I, Bagnardi V, Rota M, Scotti L, Islami F, et al. Alcohol drinking and colorectal cancer risk: an overall and dose-response meta-analysis of published studies. Ann Oncol Off J Eur Soc Med Oncol [Internet]. 2011;22(9):1958–72. Available from: http://ovidsp.ovid.com/ovidweb.cgi?T=JS&PAGE=reference&D=med7&NEWS=N&AN=213 07158

202. Zhang C, Zhong M. Consumption of beer and colorectal cancer incidence: a meta-analysis of observational studies. Cancer Causes Control [Internet]. 2015;26(4):549–60. Available from:

http://ovidsp.ovid.com/ovidweb.cgi?T=JS&PAGE=reference&D=med11&NEWS=N&AN=25 677844

203. Xu W, Fan H, Han Z, Liu Y, Wang Y, Ge Z. Wine consumption and colorectal cancer risk: a meta-analysis of observational studies. Eur J Cancer Prev [Internet]. 2019;28(3):151–8. Available from: http://ovidsp.ovid.com/ovidweb.cgi?T=JS&PAGE=reference&D=medl&NEWS=N&AN=302 47171

204. Liu L, Zhuang W, Wang R-Q, Mukherjee R, Xiao S-M, Chen Z, et al. Is dietary fat associated with the risk of colorectal cancer? A meta-analysis of 13 prospective cohort studies. Eur J Nutr [Internet]. 2011;50(3):173–84. Available from: http://ovidsp.ovid.com/ovidweb.cgi?T=JS&PAGE=reference&D=med7&NEWS=N&AN=206 97723

205. Kim M, Park K. Dietary Fat Intake and Risk of Colorectal Cancer: A Systematic Review and Meta-Analysis of Prospective Studies. Nutrients [Internet]. 2018;10(12). Available from: http://ovidsp.ovid.com/ovidweb.cgi?T=JS&PAGE=reference&D=medl&NEWS=N&AN=305 45042

206. Chen G-C, Qin L-Q, Lu D-B, Han T-M, Zheng Y, Xu G-Z, et al. N-3 polyunsaturated fatty acids intake and risk of colorectal cancer: meta-analysis of prospective studies. Cancer Causes Control [Internet]. 2015;26(1):133–41. Available from: http://ovidsp.ovid.com/ovidweb.cgi?T=JS&PAGE=reference&D=med11&NEWS=N&AN=25 416450

207. Aune D, Chan DSM, Lau R, Vieira R, Greenwood DC, Kampman E, et al. Carbohydrates, glycemic index, glycemic load, and colorectal cancer risk: a systematic review and meta- analysis of cohort studies. Cancer Causes Control [Internet]. 2012;23(4):521–35. Available from: http://ovidsp.ovid.com/ovidweb.cgi?T=JS&PAGE=reference&D=med8&NEWS=N&AN=224 18776

208. Lai R, Bian Z, Lin H, Ren J, Zhou H, Guo H. The association between dietary protein intake and colorectal cancer risk: a meta-analysis. World J Surg Oncol [Internet]. 2017;15(1):169. Available from: http://ovidsp.ovid.com/ovidweb.cgi?T=JS&PAGE=reference&D=med13&NEWS=N&AN=28 886717

209. Aune D, Chan DSM, Lau R, Vieira R, Greenwood DC, Kampman E, et al. Dietary fibre, whole grains, and risk of colorectal cancer: systematic review and dose-response meta-analysis of prospective studies. BMJ [Internet]. 2011;343:d6617. Available from: http://ovidsp.ovid.com/ovidweb.cgi?T=JS&PAGE=reference&D=med7&NEWS=N&AN=220 74852

210. Bo Y, Sun J, Wang M, Ding J, Lu Q, Yuan L. Dietary flavonoid intake and the risk of digestive tract cancers: a systematic review and meta-analysis. Sci Rep [Internet]. 2016;6:24836. Available from: http://ovidsp.ovid.com/ovidweb.cgi?T=JS&PAGE=reference&D=med12&NEWS=N&AN=27 112267

211. Chang H, Lei L, Zhou Y, Ye F, Zhao G. Dietary Flavonoids and the Risk of Colorectal Cancer: An Updated Meta-Analysis of Epidemiological Studies. Nutrients [Internet]. 2018;10(7). Available from: http://ovidsp.ovid.com/ovidweb.cgi?T=JS&PAGE=reference&D=med13&NEWS=N&AN=30 041489

© 2021 Veettil SK et al. *JAMA Network Open.*

212. Grosso G, Godos J, Lamuela-Raventos R, Ray S, Micek A, Pajak A, et al. A comprehensive meta-analysis on dietary flavonoid and lignan intake and cancer risk: Level of evidence and limitations. Mol Nutr Food Res [Internet]. 2017;61(4). Available from: http://ovidsp.ovid.com/ovidweb.cgi?T=JS&PAGE=reference&D=med13&NEWS=N&AN =27 943649

213. He X, Sun L-M, X. H, He X, Sun L-M, X. H. Dietary intake of flavonoid subclasses and risk of colorectal cancer: Evidence from population studies. Oncotarget [Internet]. 2016;7(18):26617–27. Available from: http://www.impactjournals.com/oncotarget/index.php?journal=oncotarget&page=article&op=d ownload&path%5B%5D=8562&path%5B%5D=25493

214. Jiang R, Botma A, Rudolph A, Husing A, Chang-Claude J. Phyto-oestrogens and colorectal cancer risk: a systematic review and dose-response meta-analysis of observational studies. Br J Nutr [Internet]. 2016;116(12):2115–28. Available from: http://ovidsp.ovid.com/ovidweb.cgi?T=JS&PAGE=reference&D=med12&NEWS=N&AN =28 091359

215. Panic N, Nedovic D, Pastorino R, Boccia S, Leoncini E. Carotenoid intake from natural sources and colorectal cancer: a systematic review and meta-analysis of epidemiological studies. Eur J Cancer Prev [Internet]. 2017;26(1):27–37. Available from: http://ovidsp.ovid.com/ovidweb.cgi?T=JS&PAGE=reference&D=med13&NEWS=N&AN =26 960163

216. Heine-Broring RC, Winkels RM, Renkema JMS, Kragt L, van Orten-Luiten A-CB, Tigchelaar EF, et al. Dietary supplement use and colorectal cancer risk: a systematic review and meta- analyses of prospective cohort studies. Int J cancer [Internet]. 2015;136(10):2388–401. Available from: http://ovidsp.ovid.com/ovidweb.cgi?T=JS&PAGE=reference&D=med11&NEWS=N&AN =25 335850

217. Liu Y, Yu Q, Zhu Z, Zhang J, Chen M, Tang P, et al. Vitamin and multiple-vitamin supplement intake and incidence of colorectal cancer: a meta-analysis of cohort studies. Med Oncol [Internet]. 2015;32(1):434. Available from: http://ovidsp.ovid.com/ovidweb.cgi?T=JS&PAGE=reference&D=med11&NEWS=N&AN =25 491145

218. Meng Y, Sun J, Yu J, Wang C, Su J. Dietary Intakes of Calcium, Iron, Magnesium, and Potassium Elements and the Risk of Colorectal Cancer: a Meta-Analysis. Biol Trace Elem Res [Internet]. 2019;189(2):325–35. Available from: http://ovidsp.ovid.com/ovidweb.cgi?T=JS&PAGE=reference&D=medl&NEWS=N&AN= 301 71595

219. Qiao L, Feng Y. Intakes of heme iron and zinc and colorectal cancer incidence: a meta-analysis of prospective studies. Cancer Causes Control [Internet]. 2013;24(6):1175–83. Available from: http://ovidsp.ovid.com/ovidweb.cgi?T=JS&PAGE=reference&D=med9&NEWS=N&AN =235 68532

220. Chen G-C, Pang Z, Liu Q-F. Magnesium intake and risk of colorectal cancer: a meta-analysis of prospective studies. Eur J Clin Nutr [Internet]. 2012;66(11):1182–6. Available from: http://ovidsp.ovid.com/ovidweb.cgi?T=JS&PAGE=reference&D=med8&NEWS=N&AN =230 31849

221. Li P, Xu J, Shi Y, Ye Y, Chen K, Yang J, et al. Association between zinc intake and risk of digestive tract cancers: a systematic review and meta-analysis. Clin Nutr [Internet].

2014;33(3):415–20. Available from: http://ovidsp.ovid.com/ovidweb.cgi?T=JS&PAGE=reference&D=med10&NEWS=N&AN=24 148607

222. Zhou Z-Y, Wan X-Y, Cao J-W. Dietary methionine intake and risk of incident colorectal cancer: a meta-analysis of 8 prospective studies involving 431,029 participants. PLoS One [Internet]. 2013;8(12):e83588. Available from: http://ovidsp.ovid.com/ovidweb.cgi?T=JS&PAGE=reference&D=med9&NEWS=N&AN=243 40103

223. Aicr, WCRF. Diet, nutrition, physical activity and colorectal cancer.

224. Kato I, Akhmedkhanov A, Koenig K, Toniolo PG, Shore RE, Riboli E. Prospective study of diet and female colorectal cancer: The New York University Women's Health Study. Nutr Cancer [Internet]. 1997 [cited 2020 Jul 21];28(3):276–81. Available from: https://www.tandfonline.com/doi/abs/10.1080/01635589709514588

225. Pietinen P, Malila N, Virtanen M, Hartman TJ, Tangrea JA, Albanes D, et al. Diet and risk of colorectal cancer in a cohort of Finnish men. Cancer Causes Control [Internet]. 1999 [cited 2020 Jul 21];10(5):387–96. Available from: https://link.springer.com/article/10.1023/A:1008962219408

226. Järvinen R, Knekt P, Hakulinen T, Rissanen H, Heliövaara M. Dietary fat, cholesterol and colorectal cancer in a prospective study. Br J Cancer [Internet]. 2001 Aug 3 [cited 2020 Jul 21];85(3):357–61. Available from: http://www.bjcancer.com

227. Wie GA, Cho YA, Kang HH, Ryu KA, Yoo MK, Kim YA, et al. Red meat consumption is associated with an increased overall cancer risk: A prospective cohort study in Korea. Br J Nutr [Internet]. 2014 Jul 28 [cited 2020 Jul 21];112(2):238–47. Available from: https://doi.org/10.1017/S0007114514000683

228. Chen J, Stampfer MJ, Hough HL, Garcia-Closas M, Willett WC, Hennekens CH, et al. A Prospective Study of N-Acetyltransferase Genotype, Red Meat Intake, and Risk of Colorectal Cancer. Cancer Res. 1998;58(15).
